# Gut microbiota dynamics and functionality in *Reticulitermes grassei* after a 7-day dietary shift and ciprofloxacin treatment

**DOI:** 10.1371/journal.pone.0209789

**Published:** 2018-12-27

**Authors:** Mercedes Berlanga, Montserrat Palau, Ricardo Guerrero

**Affiliations:** 1 Department of Biology, Environment and Health, Section Microbiology, Faculty of Pharmacy and Food Sciences, University of Barcelona, Barcelona, Spain; 2 Laboratory of Molecular Microbiology and Antimicrobials, Department of Pathology and Experimental Therapeutics, Faculty of Medicine, University of Barcelona, IDIBELL, Barcelona, Spain; 3 Academia Europaea-Barcelona Knowledge Hub, Barcelona, Spain; University of Illinois at Urbana-Champaign, UNITED STATES

## Abstract

Gut microbial structure in animals depends on the host, dietary habits and local environment. A random event, dietary change or antibiotic treatment may alter the gut environment with possible repercussions for the bacterial community composition and functionality and ultimately host fitness. The present study was focused on the composition, structure and functionality of gut microbiota in *Reticulitermes grassei* and the data obtained was compared with sequence surveys of three other *Reticulitermes* species. Each *Reticulitermes* species had a significantly different bacterial gut microbiota (pairwise significance tests using the Kolmogorov-Smirnov test), but a similar pattern of distribution (*P*-test in weighted Unifrac). The core gut microbiota from the analyzed *Reticulitermes* species contained 16 bacterial operational taxonomic units. Enzymes (KO) were detected from 14 pathways related to carbohydrate metabolism. *R*. *grassei* and *R*. *hesperus*, based on relative abundance of KO, had the most similar carbohydrate pathway patterns. In addition, we described the gut microbiota and functionality pathways in *R*. *grassei* after a 7-day dietary shift and antibiotic (ciprofloxacin) treatment. Both factors, but above all the antibiotic, altered the relative abundance of certain microbial groups, although the changes were not statistically significant (*P*-test in weighted Unifrac). The cellulose diet enhanced the carbohydrate pathways related to propanoate, butanoate, ascorbate, and glyoxylate metabolism. The antibiotic treatment affected galactose metabolism, the citrate cycle and inositol phosphate metabolism. Those functional changes may be related to changes in the abundance of several bacterial groups. Our findings provide insights into the stability of the gut microbiota in *R*. *grassei* and a resilience response to dietary shift or antibiotic treatment disturbance after 7 days.

## Introduction

Termites are unique among social insects because they undergo incomplete metamorphosis and display a diversified caste polyphenism [1]. The castes of workers, soldiers, reproductives, and undifferentiated immature forms cooperate in an integrated manner for the thriving of termite society [2–5]. Termites (Isoptera), cockroaches, and mantids form a well-established lineage of insects, the Dictyoptera. Termites evolved within the cockroaches around 150 Myr ago (*ca*. at the end of the Jurassic period) [6]. Six families of termites (collectively called “lower termites”, *Mastotermitidae*, *Kalotermitidae*, *Hodotermitidae*, *Termopsidae*, *Rhinotermitidae* and *Serritermitidae*) and the cockroach *Cryptocercus* hold a characteristic community of gut microbiota (mainly bacteria and protists). Higher termites, the family *Termitidae*, have lost their gut protists, having only bacteria [7]. Termites feed almost exclusively on lignocellulose in various stages of decomposition. Wood-feeding is a successful feeding mode observed in several animals, but only after key hurdles have been overcome, such as the low nutrient content, indigestibility or toxicity of many plant tissues [8]. Gut microbiota in wood-feeding insects provide their host with nutrients such as nitrogen and vitamins in appropriate quantities and balance [9, 10].

The present study focused on the microbial composition, structure and functionality of gut microbiota in *Reticulitermes grassei* (a subterranean termite native to the Iberian Peninsula and southwest France) [11]. *Reticulitermes* (*Rhinotermitidae*) is a Holarctic genus of subterranean termites widespread in Western Europe, North America (in the USA), and in Eastern Asia (mostly in Japan and China). Recent analysis (mitochondrial and nuclear sequences) showed that diversification of the genus occurred during the early Miocene, about 18.4 Myr ago, later than the *Reticulitermes* fossils found in Baltic amber deposits with an estimated age of ~38 Myr (late Eocene) [12]. The genus *Reticulitermes* presents traits that can vary among the species, especially in two aspects: (i) social organization, such as reproductive systems and colony breeding structures [2, 13] and (ii) gut microbiota [14, 15].

The data obtained from *R*. *grassei* were compared with sequence surveys of other *Reticulitermes* species [16, 17] in order to define the bacterial composition, the possible core microbiota, and metabolic pathway functions. In addition, we described gut microbiota and functionality pathways in *R*. *grassei* after a 7-day dietary shift and antibiotic (ciprofloxacin) treatment, after which the changes in microbiota were not significiant enough to cause dysbiosis.

Diet is considered one of the most important environmental factors that influences the assembly of gut microbiota [18–20]. Our knowledge about the diet-microbiota relationship comes from studies using artificial diets to assess the effects of single nutrient components [16, 21–23]. These controlled studies provide insight into how a specific aspect of an organism’s diet influences the gut microbiota. In termites, the use of antibiotics can induce different degrees of dysbiosis that significantly alters: (i) fitness and lifespan (treatment with rifampin at 0, 14 and 34 days) (24); (ii) termite reproduction and colony establishment (treatment with rifampin at 0, 14 and 34 days) [24]; and (iii) changes in lignocellulolytic capacity after a 7-day treatment with ampicillin, kanamycin, metronidazole and tetracycline [25]. However, no previous studies show the microbial composition and functionality in *R*. *grassei* during a 7-day ciprofloxacin (antibiotic) treatment. The findings reported herein provide insights into the dynamic stability of the gut microbiota in *R*. *grassei* and resilience response to diet and antibiotic perturbation.

## Material and methods

### Sample collection and DNA extraction from *Reticulitermes grassei*

*Reticulitermes grassei* workers were obtained from their natural environment, a forest composed mostly of pines and, to a lesser degree, holm oaks in the Serra del Corredor (Barcelona, Spain). Serra del Corredor is not a protected area. It is not necessary specific permission for work in the area, and the field studies did not involve endangered species. Samples were collected from a colony in a pine stump. In the laboratory, three groups of *ca*. 60 workers from the same colony were placed in 3.5-cm Petri dishes containing the respective diet and antibiotic treatment: (i) natural diet (a piece of sterilized pine wood from the original wood where the termites were collected in the field) (Rg_1); (ii) pieces of sterilized filter paper, size of 10 mm^2^ (Whatmann grade 4) (Rg_2); and (iii) pieces of sterilized wood + ciprofloxacin (antibiotic) (Rg_3). The antibiotic treatment consisted of moistening the wood pieces with 200 μl of ciprofloxacin at a concentration of 1 mg/ml, on alternate days. Termites in treatments 1 to 3 were maintained at room temperature for 7 days, after which 50 individuals from each treatment were dissected. Termite survival after the cellulose diet and ciprofloxacin treatment was similar to that of the control samples, although we have previously observed that a longer stay in the Petri dish causes termite death, probably due to the low humidity environment.

For each group, DNA extraction was performed in triplicate on samples each containing 16 termite guts. Guts were removed using sterilized forceps and placed on TE buffer in Eppendorf tubes for DNA extraction. The whole guts were homogenized using a FastPrep system (MP Biomedicals Europe) with 0.1-mm glass beads. Bulk DNA was extracted by several washings with phenol-chloroform. All material and solutions were sterile. Dissection and extraction were carried out in an aseptic environment (laminar flow cabinet) under a laminar hood to avoid contamination [26]. We prepared three amplicon libraries for each group, and then we mixed equimolarly three of them to obtained for each group one amplicon library for pyrosequencing. A representative microbiota was obtained for each analysis: the control (wood diet), cellulose diet and antibiotic treatment. The aim was to ensure as far as possible that the difference in gut microbiota was due to the treatment and not potential interindividual variation, although interindividual variation in termites may be low. Microbial communities sampled from the same colony (biological replicate) after different diet treatments were found to show high similarity [21], possibly because the termite worker caste transfers food stomodeally (by regurgitation) and/or proctodeally (by excretion of the hindgut contents) to maintain a uniform microbiome throughout the colony [21, 26, 27].

### Amplicon library preparation from *R*. *grassei*

We performed amplicon sequencing of the bacterial 16S rRNA gene. The primers used for multiplex Roche 454 GS FLX pyrosequencing were the universal bacterial nucleotide sequences for the region V1–V2, 8F-338R (5´-GAGTTTGATCCTGGCTCAG-3´ and 5´-TGCTGCCTCCCGTAGGAGT-3´). PCR conditions and purification were performed as in Berlanga et al. (2017). Pyrosequencing coverage (depth sequencing) resulted in 170,134 total raw reads that after quality control processing resulted in 30,751 reads (see bioinformatics analyses section) for the *R*. *grassei* samples. Data deposition: Bioproject PRJNA482509.

### Bioinformatics analyses of *R*. *grassei* and other *Reticulitermes* species

For 16S rRNA amplicons, the raw data of each sample was preprocessed for demultiplex and quality control using a pipeline implemented in GPRO version 1.1 [28]. Raw reads that contained < 150 nucleotides in size, ambiguities > 1, homopolymer > 8, as well as redundant sequences were removed from each amplicon dataset using screen.seqs and unique.seqs by Mothur1.31.2 [29].

Taxonomy was assigned by the Silva database (http://www.arb-silva.de) [30]. Alpha and beta diversity analyses of all samples were performed at a 97% distance level of operational taxonomic units (OTU). For diversity, samples were rarified (normalized) so all the samples could be compared. Weighted UniFrac metrics were used to measure beta-diversity and to generate principal coordinate analysis plots, using the normalized OTU table. For the heatmap analysis Pearson’s correlation was used for similarity, and for the clustering algorithms, Ward’s linkage. Stamp v2.1.1 software was used to statistically analyze (visualize) taxonomic and metabolic profiles [31]. Core microbiota were determined using compute_core_microbiome.py in qiime (http://qiime.org/scripts/compute_core_microbiome.html) [32]. Core OTUs are defined as the OTUs present in at least 90% of the samples.

Functionality was predicted from the 16S rRNA data using PICRUSt and Tax4Fun. PICRUSt analysis was performed by the predict_metagenomes.py script run against the functional database of KEGG Orthology [33]. Functional contributions of various taxa to different KOs were computed with the script metagenome_contributions.py [34]. For the Tax4Fun analysis, the web-based tool MicrobiomeAnalyst (http://www.microbiomeanalyst.ca) was used [35].

Diversity and functionality analyses were performed in *R*. *grassei* (Rg_1, pine diet; Rg_2, cellulose diet; Rg_3, ciprofloxacin treatment) (this work). In addition, previously published data were used to determine microbiota diversity and functionality of the intestinal tract of several species of *Reticulitermes*: *R*. *flavipes*_1 (Juden Creek Nature Area, Missouri, Bioproject PRJNA172449) [16], *R*. *flavipes*_2 (Iowa, USA), *R*. *hesperus* (Galiano Island, Canada) and *R*. *virginicus* (Florida) (Bioproject PRJNA238270) [17]. In all those samples, taxonomic characterization of the gut bacterial composition was carried out using the variable regions (V1–V3) of the 16S rRNA and sequenced by the 454 GSFLX titanium sequencing platform. Representative Asian *Reticulitermes* species (e.g., *R*. *speratus*) were not included in the comparative study because no database using the variable region V1-V3 and the same sequencing platform was found.

For *R*. *grassei* (Rg_1) and *R*. *flavipes*_1, the diet consisted of pine wood (*Pinus* sp.), although in *R*. *flavipes*_1 sterile wood diet experiments were also performed for six weeks in the laboratory. For the others, the diet was also wood from the pine family or other conifers, e.g., *R*. *hesperus* was sampled in Galiano Island (Canada), where forests of cedars (*Cedrus* sp.) and Douglas firs (*Pseudotsuga menziesii*) are predominant.

## Results

### Microbiota composition in *Reticulitermes grassei* and *R*. *flavipes*, *R*. *heperus* and *R*. *virginicus*

**Fig 1:**
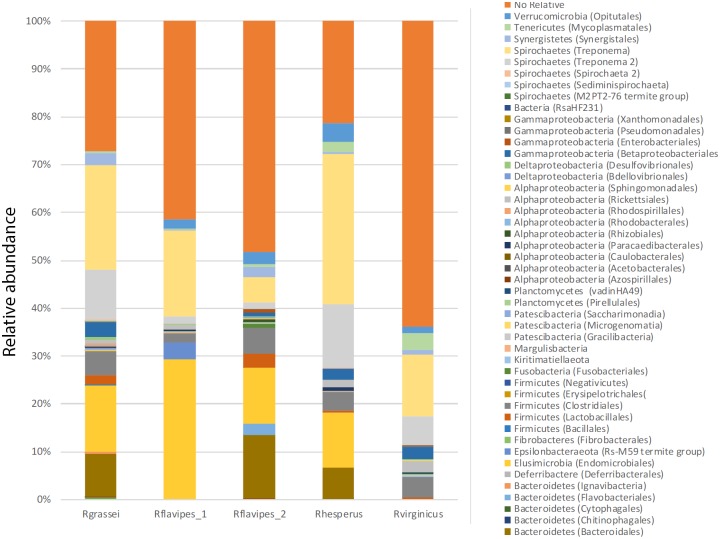
Relative abundances of bacterial phyla in gut microbiota from four *Reticulitermes* species. *R*. *grassei* (Spain), *R*. *flavipes* (USA), *R*. *hesperus* (Canada) and *R*. *virginicus* (USA).

In the four wood-feeding *Reticulitermes* species (*R*. *grassei*, *R*. *flavipes*, *R*. *heperus* and *R*. *virginicus*), the most abundant bacterial phyla were Spirochaetes (32.4%; 19.5–6.62%; 44.5% and 18.9%), Elusimicrobia (13.9%; 29.2–11.6%; 11,5%; 10,2%), Bacteroidetes (9.3%; 1.8–15.5%; 6.5%; 4.5%), Firmicutes (7%; 2–8.3%; 4.2%; 4.7%) and Proteobacteria (5.2%; 1.4–2.7%; 4.4%; 5.9%) (Fig 1). Sequences that could not be placed into any recognized phylum ranged from 20% to 61%. Heatmaps of the most abundant OTUs identified in the gut of several *Reticulitermes* species were shown in S1 Fig. Clustering analysis revealed that the gut bacterial pattern composition of *R*. *grassei* is similar to that of *R*. *hesperus* (S1 Fig).

Pairwise significance tests using the Kolmogorov-Smirnov test based on representative OTUs showed significant differences between *Reticulitermes* species, including the two studied samples of *R*. *flavipes*. To determine which taxa groups differ from each pairwise *Reticulitermes* species, extended error bar plots were generated to indicate statistically significant features (*P* < 0.05) [31]. Relative abundances of families detected in *R*. *grassei* that were significantly different with respect to *R*. *flavipes* were *Spirochaetaceae*, *Dethiosulfovibrionaceae*, *Porphyromonadaceae*, unclassified *Clostridiales*, unclassified *Bacteroidales* and *Synergistaceae*, the first two being more abundant in *R*. *grassei* than *R*. *flavipes*. Differences in family proportion between *R*. *grassei* and *R*. *hesperus* were in *Spirochaetaceae*, *Dethiosulfovibrionaceae*, *Ruminococcaceae*. The comparison between *R*. *grassei* and *R*. *virginicus* showed similar microbiotas to *R*. *hesperus* with the addition of Endomicrobia. In the case of *R*. *flavipes*_1 and *R*. *flavipes*_2, the differences were significant due to the unassigned sequences of Endomicrobia, *Porphyromonadaceae*. *Mycoplasmataceae* and unclassified *Rickettsiales* (S2 Fig).

**Fig 2:**
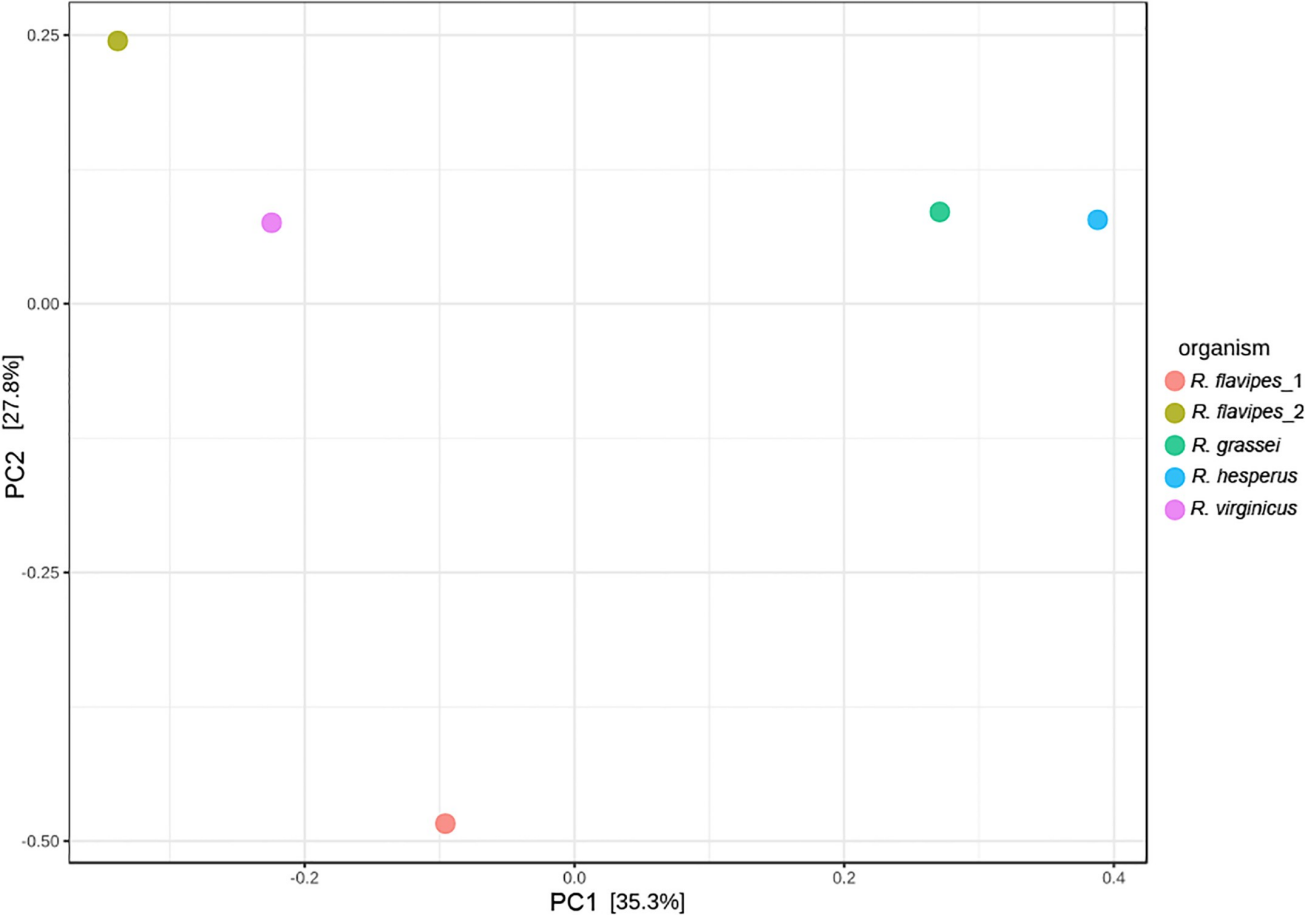
Principal component analysis of the community distribution. β-Diversity coupled with principal coordinates analysis was used to compare the bacterial composition in *Reticulitermes* species.

Shannon’s diversity index showed that the intestinal tracts of *Reticulitermes* species support a highly diverse community of bacteria, comparable with other wood- or herbivorous-feeding insects [26, 36]. Simpson’s evenness and Berger–Parker dominance indexes showed similar values among the analyzed *Reticulitermes* species except for *R*. *flavipes*_1 (Table 1), probably because the latter underwent a six-week sterilized pine wood food treatment in laboratory conditions. Results showed that every *Reticulitermes* species contained different gut bacterial community patterns, clustering into two groups: *R*. *grassei*/*R*. *hesperus* and *R*. *flavipes*/*R*. *virginicus*. Nevertheless, principal coordinate analysis and *P*-test in weighted Unifrac indicated that microbial communities were not significantly different (*P* > 0.05) (Fig 2).

**Table 1:** Alpha diversity at 0.03 distance in four *Reticulitermes* species.

	*R*. *flavipes*_1	*R*. *flavipes*_2	*R*. *hesperus*	*R*. *virginicus*	*R*.*grassei* (Rg_1)	Rg_2	Rg_3
Shannon	3.41	4.89	4.29	4.43	4.34	4.25	4.12
Simpson	0.823	0.951	0.909	0.93	0.924	0.935	0.896
Bergerparker	0.349	0.104	0.194	0.161	0.178	0.160	0.201

*R*. *grassei*: Rg_1 (pine wood diet), Rg_2 (cellulose diet); Rg_3 (ciprofloxacin treatment).

**Fig 3:**
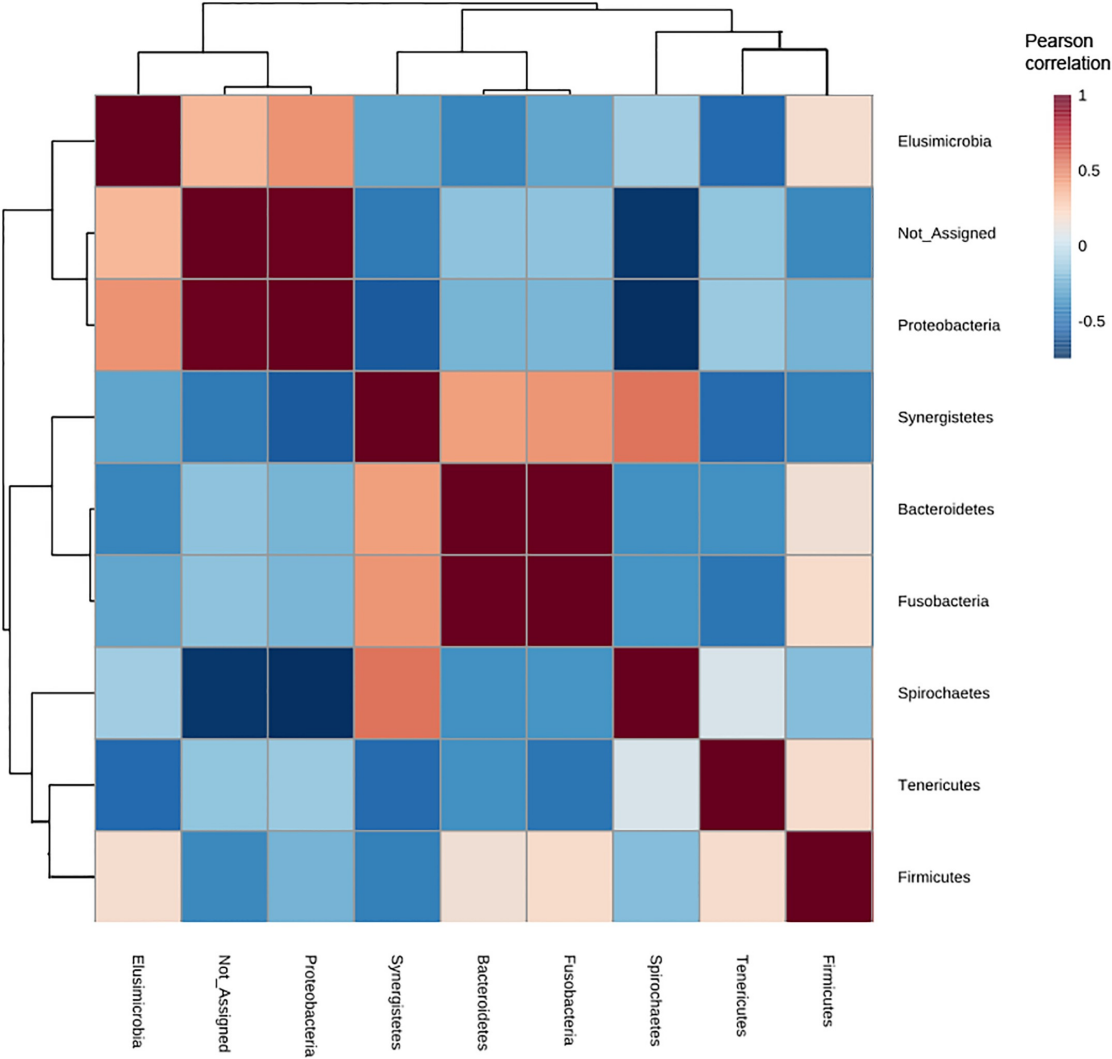
Pearson’s linear correlation matrix of the selected taxa. Strong positive correlations are indicated by dark red (positive) and strong negative correlations by dark blue.

Fig 3 showed the correlation matrix of phyla associated with gut microbiota of several *Reticulitermes* species, indicating meaningful relationships or associations between taxa. Elusimicrobia presented positive interactions with Firmicutes, Proteobacteria and several bacteria from the unclassified group. Bacteroidetes showed strong positive correlation with Fusobacteria. Spirochaetes showed strong negative correlation with Proteobacteria, negative correlation with Firmicutes, but positive correlation with Synergistetes.

As mentioned, every *Reticulitermes* species had different bacterial gut microbiota, but a similar pattern of distribution (beta-diversity). We were interested in finding the conserved or permanent microbiota in *Reticulitermes* by characterizing the core microbiota, which could reflect which microbiota coevolved with the *Reticulitermes* host before the species diversification. Core OTUs were determined by the shared OTUs at 90% in all samples. Core gut microbiota from *Reticulitermes* species contained 16 bacterial OTUs: unassigned bacteria (1 OTU), Verrucomicrobia (1 OTU), Plantomycetes (1 OTU), Tenericutes–*Mycoplasmataceae* (1 OTU), Betaproteobacteria (1 OTU), Bacteroidetes (1 OTU), Firmicutes–Clostridia (2 OTU), Alphaproteobacteria (2 OTU), Synergistetes (2 OTU), and Spirochaetes (4 OTU).

### Microbiota functionality from *Reticulitermes grassei* and *R*. *flavipes*, *R*. *hesperus* and *R*. *virginicus*

**Fig 4:**
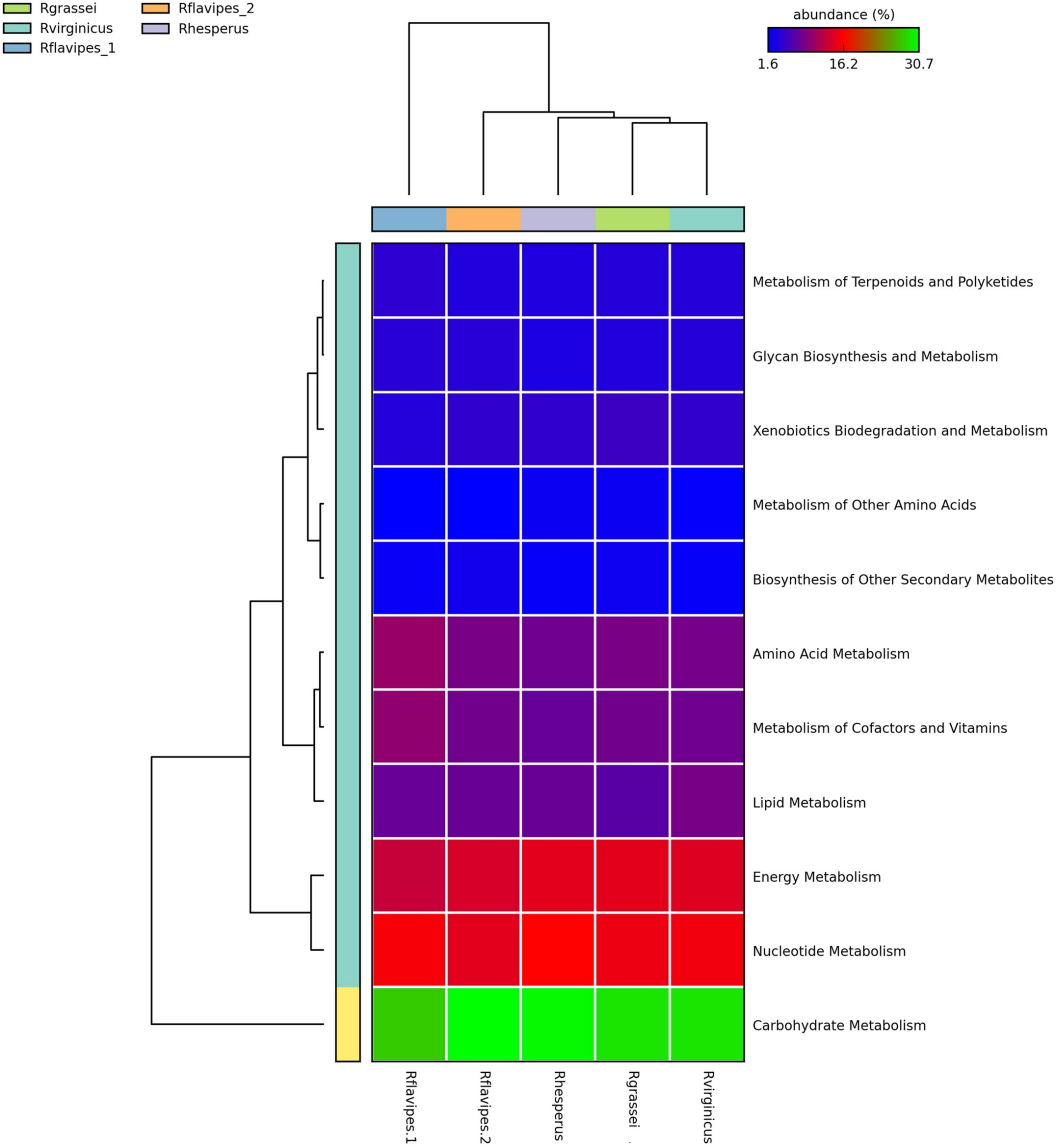
Heatmap and dendogram of the relative abundance of metabolic pathways from *Reticulitermes* species gut bacterial communities.

The nearest sequence taxon index (NSTI) value is a measure of how closely related the OTUs in each sample are to the reference genomes in the database. In our case, NSTI values per sample were in the range of 0.070–0.181. The taxonomic classification was accurate at the family level, and in several cases at genus level, but few OTUs could be classified at the species level. This result could explain the NSTI values obtained. The identified biological processes are essential for sustaining prokaryotic life in the environment, and they include genetic information functions (14.9–19.1% based on the total number of genes detected in the sample), cellular processes such as cell motility (5.8–6.9%), genes related to membrane transport (16.45–18.8%) and metabolic functions (55.4–59.9%). Of all the metabolic process genes detected, those associated with carbohydrate metabolism were the most abundant (Fig 4). We focused on carbohydrate metabolism in order to study the functional metabolism associated with diet.

To determine differences in carbohydrate metabolism pathways from each pairwise comparison of *Reticulitermes* species, extended error bar plots were generated to show statistically significant differences *P* < 0.05 (31) (S3A Fig). We detected enzymes (KO) from 14 pathways related to carbohydrate metabolism. *R*. *grassei* and *R*. *hesperus*, based on relative abundance of KO, had the most similar carbohydrate pathway pattern (S3A Fig). Principal coordinates analysis of carbohydrate metabolism suggested two groups: *R*. *grassei*/*R*. *hesperus* and *R*. *flavipes*/*R*. *virginicus* (S3B Fig). These results may be linked to the bacterial gut taxonomic composition.

### Gut microbiota composition in *Reticulitermes grassei* after a 7-day dietary shift and ciprofloxacin treatment

**Fig 5:**
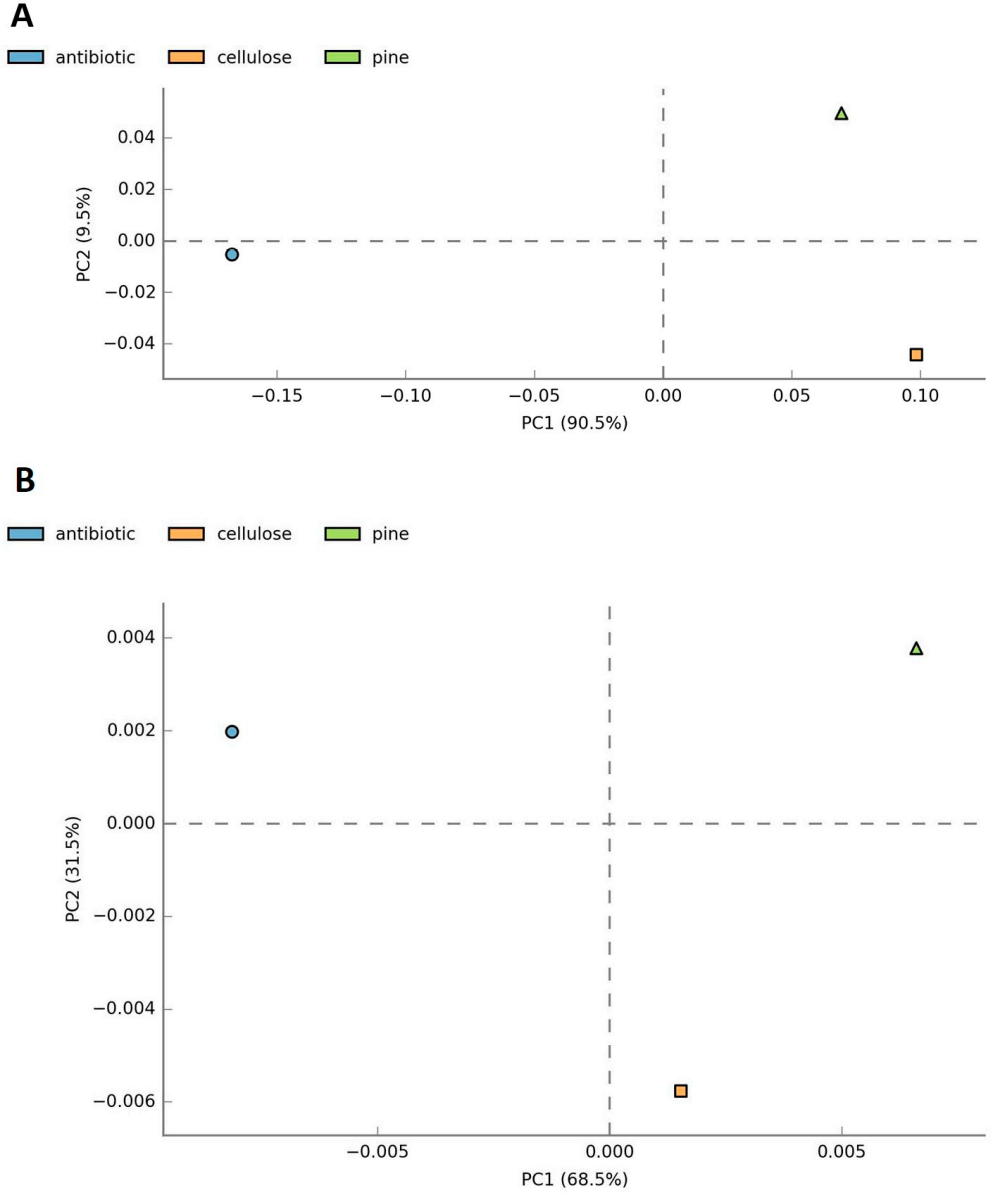
Principal component analysis of the community distribution. (A) β-Diversity coupled with principal coordinates analysis was used to compare the bacterial composition in *Reticulitermes grassei* treatments, wood-diet, cellulose-diet and ciprofloxacin treatment. (B) β-Diversity coupled with principal coordinates of functional annotation from PICRUSt analysis in *R*. *grassei* treatments, wood-diet, cellulose-diet and ciprofloxacin treatment.

Alpha diversity (Shannon index) in the wood diet was slightly higher than in the cellulose diet and antibiotic treatment (Table 1). Cellulose is a less complex food than wood, and presumably requires less complex microbiota for its digestion. Antibiotic treatment diminished diversity (due to the biocide effect of ciprofloxacin) and enhanced the dominance of several OTUs. Disturbance (diet and antibiotic) altered the relative abundance of certain microbial groups but did not induce significant differences in microbiota composition (*P*-test in weighted Unifrac *P* > 0.05). (Fig 5).

When comparing the pine and cellulose diet in *R*. *grassei*, we observed that the cellulose diet rendered an increased proportion of *Actinobacteria*, Firmicutes, Bacteroidetes, and Synergistetes and a reduced abundance of Spirochaetes (e.g. *Treponema primitia*), Endomicrobia and Delta protoeobacteria (e.g., *Desulfovibrio*). The antibiotic treatment was associated with decreased proportions of Synergistetes (TG5) and Bacteroidetes (e.g. *Candidatus* Azobacteroides, *Dysgonomonas*), with a variable effect on Spirochaetes depending on the OTUs. For example, the proportion of *Treponema primitia* decreased, whereas other Treponemes increased. A significantly higher relative abundance was observed in Endomicrobia and Deltaproteobacteria (e.g. *Desulfovibrio*) (S4 Fig).

Core microbiota in *R*. *grassei* consisted of 46 OTUs across 100% of samples (control, cellulose diet and antibiotic treatment) belonging to: Actinobacteria (*Propionibacteriaceae*, *Coriobacteriaceae*), Bacteroidetes (*Candidatus* Azobacteroides and *Dysgonomonas*), Elusimicrobia (*Endomicrobia*), Firmicutes (*Lactococcus*), Proteobacteria (*Eikenella*, *Desulfovibrio*), Spirochaetes (*Treponema*), Synergistetes (TG5), Tenericutes (*Mycoplasmataeae*), and unclassified bacteria. Of these 46 OTUs, seven belonging to Spirochaetes, Synergistetes, Tenericutes, and unclassified bacteria were conserved in other *Reticulitermes* species.

### Gut microbiota functionality in *Reticulitermes grassei* after a 7-day dietary shift and ciprofloxacin treatment

The NSTI values per sample calculated by PICRUSt were in the range of 0.070–0.112. Results of the two functional assignment strategies showed differences in functional categories as determined by KO genes. Tax4Fun detected 15.3% more KO than PICRUSt. However, when we discarded the accounts contribution by sample < 0.0001, Tax4Fun identified 4.7% more KO than PICRUSt (S1 Table). It is important to note that PICRUSt detected certain KO (23.3%) that were not identified with Tax4Fun and vice-versa (31.3%) (S1 Table). These functions were mainly related to membrane transport, translation and replication pathways.

We focused on carbohydrate metabolism (pathways associated with lignocellulose and cellulose feeding) (S5 Fig), diet-related detoxification (S6 Fig), energy metabolism (S7 Fig) and cofactors and vitamins that could affect the fitness of the host (S8 Fig). The cellulose diet enhanced the carbohydrate pathways related to propanoate, butanoate, ascorbate, and glyoxylate metabolism. Antibiotic exposition affected galactose metabolism, the citrate cycle and inositol phosphate metabolism. As an example, the contribution by phyla to K01784 (galactose pathway) particularly affected the Spirochaeta phylum (S9A Fig). Xenobiotic metabolism by cytochrome P450 was significantly affected by the cellulose diet, and the antibiotic seemed to affect the xylene degradation and the generic pathway for xenobiotic biodegradation (S9B Fig). Ciprofloxacin also appeared to affect respiring bacteria (oxidative phosphorylation) and the cellulose diet affected the metabolism of nitrogen and vitamins such as pantothenate, biotin, thiamine and riboflavin.

## Discussion

### Microbiota composition and functionality in *Reticulitermes grassei* and *R*. *flavipes*, *R*. *hesperus* and *R*. *virginicus*

The host (phylogenetics of the animal and gut environment), diet and local environment shape the gut microbial structure [17, 37]. The gut of wood-feeding “lower” termites (e.g., *Reticulitermes*) harbors a complex microbial community consisting of protists and bacteria. Many protist species are not necessarily restricted to one termite species and their abundance can vary [38]. They may also be associated with different bacterial ectosymbionts such as Spirochaetes, Bacteroidetes, Synergistetes, and Deltaproteobacteria [39], and endosymbionts such as Bacteroidetes, Elusimicrobia, methanogens (genus *Metanobrevibacter*) [40] and spirochetes [41]. Therefore, any changes in the flagellate community may give rise to shifts in the bacterial community as well [15, 17]. The natural food of *Reticulitermes* is wood, a complex material composed mainly of cellulose, hemicellulose and lignin (collectively called lignocellulose) along with other complex carbohydrates [42]. The lignocellulose composition of the diet (lignin-high, wood, or lignin-poor, cellulose) may be an important factor causing shifts in the gut microbiota [43], and could determine a preference for a particular food source [11]. Wood digestion is performed by mechanical processing by the host (termite), endogenous cellulases, the flagellate protists [44], and bacteria [25]. *Reticulitermes* genus has a “foraging lifestyle”, leaving the nest to search for food [15], so exposure to environmental microbes is also expected.

Gut bacterial composition differed in each *Reticulitermes* species (*R*. *grassei*, *R*. *flavipes*, *R*. *hesperus*, and *R*. *virginicus*). Nevertheless, we observed that the four *Reticulitermes* species analyzed shared a similar distribution pattern (beta-diversity), and principal coordinates analysis and *P*-test in weighted Unifrac indicated that the microbial communities were not significantly different (*P* > 0.05) (Figs 1 and 2, S1 Fig). The resident microbiota is thought to form long-term evolutionary significant relationships with the host [15, 17, 26]. Patterns of microbial co-occurrence and segregation could be explained by their evolutionary relatedness and functionality [45–47] (Fig 3). As an example, spirochetes are specific symbionts that have coevolved with their respective species of termites, which are stably harbored and closely related to members of the same termite family [48]. The first divergence in the ancestral lineage of *Reticulitermes* occurred in the early Miocene and separated the Nearctic lineages (i.e., the North America lineages) from the Palearctic lineages (i.e., Western Europe, Eastern Europe and Western Asia) [12]. Among the analyzed *Reticulitermes* species from North America, *R*. *virginicus* is the most distant lineage from *R*. *grassei* (a representative *Reticulitermes* species from Western Europe), followed by *R*. *flavipes*. *R*. *hesperus* is the most related lineage to *R*. *grassei* [12], which is supported by the gut bacterial composition reported here, the pattern observed for *R*. *grassei* being most closely related to that of *R*. *hesperus* (Fig 2, S1 Fig).

Of all the metabolic process genes detected by PICRUSt and Tax4Fun analyses, those associated with carbohydrate metabolism were the most abundant, reflecting their functional importance for the natural wood diet of *Reticulitermes* (Fig 4, S3 Fig, S1 Table). The recalcitrance to biodegradation of various substrates may be important for sustaining a diverse gut microbiota and microbiome in *Reticulitermes* termites [16, 49]. *R*. *grassei* and *R*. *hesperus* shared a similar pattern of KO associated with carbohydrate pathways (S3 Fig), reflecting similar microbial diversity and a wood-feeding diet.

The results indicate that the gut bacterial community profiles in *Reticulitermes* (at least in the four studied species) were related to the host phylogeny and the lignin contents of the diet (pine or coniferous tree wood) collected from their natural habitat [50]. The transient environmental microorganisms in termites collected from different geographical areas did not seem to be an important factor in the configuration of the bacterial gut community (Fig 2).

### Gut microbiota composition and function in *Reticulitermes grassei* after a 7-day dietary shift and ciprofloxacin treatment

Different diets may change the gut environment, affecting factors such as the C/N ratio, O_2_–H_2_ gradient, and intermediate metabolites, so shifts in the bacterial community are also expected [43]. Less complex food, such as a cellulose diet, can be selected by more adapted taxa able to degrade it, leading to the exclusion of other bacteria competing for carbon resources [16]. An increased abundance of several phyla was observed with the cellulose diet, such as Actinobacteria, Firmicutes, and Bacteroidetes, which appear to play important roles in carbohydrate degradation (S4 Fig) [43, 51]. Changes also occurred in the relative abundances of certain microbial groups but these did not induce significant shifts in microbiota composition after the 7-day cellulose treatment (Table 1, Fig 5A).

Our results confirm previous reports that microbial communities are sensitive to diet [16, 21], and that a significant change in the community is a feeding time-dependent event [43]. Duarte et al. [52] showed that flagellate protist communities differed between cellulose-fed and wood-feeding *R*. *grassei*, which in turn could affect the abundance of protist-associated bacteria. We observed a reduced abundance of Endomicrobia and *Desulfovibrio* (symbiont microorganisms associated with protists). The *Endomicrobia* class provides protists and the insect host with vitamins and amino acids [53]. *Desulfovibrio* retains biosynthetic pathways for various amino acids and cofactors [39]. When *R*. *grassei* was fed with cellulose, the protists *Microjoenia hexamitoides* and *Pyrsonympha* sp. increased in abundance, suggesting they intervene in cellulose degradation and displace other protists populations such as *Trichonympha agilis* [52]. The protist *Trichonympha agilis* permanently hosts two symbiotic bacteria, ‘*Candidatus* Endomicrobium trichonymphae’ and ‘*Candidatus* Desulfovibrio trichonymphae’, which have a mutualistic relationship [39]. Therefore, changes in the proportion of a protist could modify the proportion of several bacterial taxa (those associated with their protist). We also observed a decrease in several OTUs of Spirochaetes (*Treponema*), especially *Treponema primitia*. *Treponema* is responsible for the transformation of H_2_ and CO_2_ into acetate, the main source of carbon and energy for termites. Treponemes encode glycoside hydrolases, which are important enzymes in hemicellulose and lignin degradation [54].

Ciprofloxacin is a fluoroquinolone with a broad antibacterial spectrum, although fluoroquinolones show limited activity against anaerobic bacteria [55]. They inhibit the bacterial DNA gyrase (Gram-negative) and the topoisomerase IV (Gram-positive) [56]. Broad-spectrum antibiotics can affect the abundances of 30% of bacteria in the gut community, causing rapid and significant diminution in taxonomic diversity [57–59]. Gut microbial diversity is considered as a biomarker of health and metabolic capacity [60, 61]. Our results showed that 7-days of ciprofloxacin treatment affected the bacterial diversity in *R*. *grassei* (Table 1) and the relative abundances of several bacterial taxa (Fig 5A). However, this treatment produced no significant differences in microbiota composition, structure and functionality (*P*-test in weighted Unifrac *P* > 0.05), (Fig 5B). Treatment with ciprofloxacin caused a decreasing relative abundance of the free-swimming *Treponema primitia*, TG5 (Synergistetes) and Bacteroidetes such as *Azobacteroides* and *Dysgonomonas*, and an increasing relative abundance of Elusimicrobia (Endomicrobia) and *Desulfovibrio* (S4 Fig). A higher proportion of Elusimicrobia has been reported after tetracycline treatment in *R*. *flavipes* [62]. We did not analyze the effect of ciprofloxacin on the protist population in *R*. *grassei*, but protists are described as affected by antimicrobial treatment with kanamycin and tetracycline (bacterial inhibitors of protein synthesis) and metronidazole (antiprotozoan) [62]. Again, protists abundance modification could affect several associated bacterial symbionts.

The functional effect of ciprofloxacin treatment may have induced changes in several bacterial groups, such as a decreased proportion of Bacteroidetes, Synergistetes and Spirochaetes. The antibiotic seemed to affect the xylene degradation, nitrogen fixation and the generic pathway for xenobiotic biodegradation (S5–S7 Figs). Treponemes are often major contributors to xylene degradation and, to a lesser extent, nitrogen fixation in the gut microbial community [41, 63]. Bacteroidetes are related to diazotrophic bacteria such as *Azobacteroides pseudotrichonympha*, which provide amino acids and cofactors for the nutrition of the protist and termite hosts [64]. We observed that general detoxifying pathways were reduced after antibiotic treatment and this could be related with diminished relative abundances of Synergistetes. Fluoroacetate is a toxic compound synthesized by plants as a defense mechanism against herbivore grazing. The Synergistetes phylum can degrade fluoroacetate to fluoride ions and acetate, a detoxifying process that can protect the host [65].

A random event (dietary change or antibiotic treatment) may alter the environment with possible repercussions on the community composition. Understanding the drivers of microbial community stability is important for predicting the community response to disturbance, which may include resistance (where microbial composition remains unchanged by a disturbance) or resilience (where the community returns to the previous state after the disturbance, either in terms of composition (species) or function (genes)) [66, 67]. Although we did not determine both microbiota composition and functionality after the restitution of a normal diet or non-antibiotic treatment to ascertain the microbial recovery, it can be presumed that microbiota returns to the original state. This dynamic state of composition and function during a given treatment may be due to a functional redundancy, in which the remaining microbiota compensates for the changes in other members.

The 7-day period of dietary shift and antibiotic treatment was not long enough to cause a significant change in microbiota and general function, but a dynamic equilibrium in the bacterial gut community of *R*. *grassei* was observed. A multiplicity of treatments over time can erode the resilience of the community [68]. A disturbance of the environment close to a turning point can generate an abrupt change in the microbial composition, which may not be able to return to the previous stable state. In the case of the gut microbiota, this new situation could be associated with a state of health or disease.

The work carried out provides insights into how antibiotic treatment and dietary changes may disturb the structure and function of a gut bacterial community and the relationship between its members (bacteria and protists). To further assess the impact of diet/antibiotics on the microbiome and its subsequent re-establishment, and understand which conditions lead to an irreversible change affecting host survival, tests need to be performed over a longer period. Establishing which factors cause permanent effects could result in gut microbiota modification becoming a strategy for pest control.

## Supporting information

S1 Table Comparison KO functions between Tax4Fun and PICRUST (contribution OTU by sample > 0.0001). (PDF)

S1 Fig Heatmap and dendogram of the relative abundance of OTU identified in the *Reticulitermes* species. Several representative OTUs could be classified at genus level.
(TIF)

S2 Fig Extended error bar plot identifying significant differences between mean proportions of bacterial taxa in pairwise *Reticulitermes* species. Corrected *P* values are shown at right.
(TIF)

S3 Fig Extended error bar plot and principal component analysis of carbohydrate pathways. (A) Extended error bar plot identifying significant differences between mean proportions of carbohydrate pathways in pairwise *Reticulitermes* species. (B) PCA of carbohydrate pathways from *Reticulitermes* species.
(TIF)

S4 Fig Extended error bar plot. Extended error bar plot identifying significant differences between mean proportions of bacterial taxa in pairwise *Reticulitermes grassei* treatments, wood-diet, cellulose-diet and ciprofloxacin treatment.
(TIF)

S5 Fig Extended error bar plot. Extended error bar plot identifying significant differences between mean proportions of carbohydrate pathways in pairwise *Reticulitermes grassei* treatments, wood-diet, cellulose-diet and ciprofloxacin treatment.
(TIF)

S6 Fig Extended error bar plot. Extended error bar plot identifying significant differences between mean proportions of xenobiotic pathways in pairwise *Reticulitermes grassei* treatments, wood-diet, cellulose-diet and ciprofloxacin treatment.
(TIF)

S7 Fig Extended error bar plot. Extended error bar plot identifying significant differences between mean proportions of energy pathways in pairwise *Reticulitermes grassei* treatments, wood-diet, cellulose-diet and ciprofloxacin treatment.
(TIF)

S8 Fig Extended error bar plot. Extended error bar plot identifying significant differences between mean proportions of amino acid, cofactors and vitamins metabolism in pairwise *Reticulitermes grassei* treatments, wood-diet, cellulose-diet and ciprofloxacin treatment.
(TIF)

S9 Fig Phyla contribution by KO genes. (A) Phyla contribution by percent of sample respect to K01784 (galactose pathway). (B) Phyla contribution by percent of sample respect to K00446 (xylene degradation).
(TIF)
