# Supplementary material for: Gut microbiota dynamics and functionality in Reticulitermes grassei after a 7-day dietary shift and ciprofloxacin treatment
Source: PLoS One. 2018 Dec 27;13(12):e0209789. doi: 10.1371/journal.pone.0209789 (PMC6307977; doi:10.1371/journal.pone.0209789)
Supplement: S1 Table — (PDF) [file pone.0209789.s001.pdf]

Comparison KO functions between Tax4Fun and PICRUST (contribution OTU by sample > 0.0001)

Tax4Fun				PICRUSt		
	Rg_3	Rg_2	Rg_1		Rg_3	Rg_2
K00001	0.00029068	0.00034822	0.000285660294773554	K00003	0,000747	0,000378
K00003	0.00025798	0.00025671	0.000244924429489988	K00008	0,000179	0,000395
K00012	0.00064861	0.00055111	0.000856158651504259	K00010	0,000341	0,000773
K00013	0.00031252	0.00036564	0.000385877189050248	K00012	0,000406	0,000625
K00014	0.00027787	0.00040978	0.000424735704689585	K00013	0,000845	0,000411
K00018	0.00013790	0.00010140	0.000138572887838308	K00014	0,000877	0,000378
K00024	0.00104173	0.00090389	0.000891891773024247	K00020	0,000374	0,000263
K00029	0.00222639	0.00184128	0.00162759568148493	K00024	0,000162	0,000164
K00031	0.00128528	0.00115744	0.00106026741769467	K00027	0,00078	0,000805
K00033	0.00032288	0.00043861	0.000736314459522511	K00029	0,000179	0,000181
K00036	0.00033089	0.00053828	0.000723889725467858	K00031	0,000276	0,00023
K00052	0.00039010	0.00047118	0.000605432869573038	K00036	0,000309	0,000132
K00053	0.00029834	0.00034200	0.000397152005863184	K00040	0,000309	0,00051
K00057	0.00101554	0.00086228	0.00093229262184779	K00042	0,000276	0,000427
K00058	0.00041480	0.00059619	0.000563291591700803	K00050	0,000211	0,00046
K00059	0.00173111	0.00227710	0.00177482812227446	K00052	0,00078	0,000362
K00067	0.00040800	0.00050380	0.000655943877608633	K00053	0,000894	0,000658
K00075	0.00080928	0.00072964	0.000640233036280392	K00057	0,000975	0,000904
K00088	0.00064957	0.00071642	0.000899357576673162	K00058	0,00065	0,000838
K00097	0.00030937	0.00051350	0.000549515283170661	K00059	0,002242	0,001447
K00099	0.00137283	0.00115291	0.00122119684702715	K00067	0,000292	0,000592
K00100	0.00029722	0.00058599	0.000477947608849131	K00074	0,000292	0,000279
K00102	0.00026171	0.00026507	0.000163905059505042	K00075	0,000959	0,000904
K00104	0.00054938	0.00040102	0.000327901595654569	K00077	0,000276	0,00051
K00111	0.00042925	0.00048500	0.00020749848156611	K00088	0,001007	0,000575
K00123	0.00183409	0.00073194	0.0008998915222772	K00091	0,000211	0,000378
K00124	0.00039439	0.00014621	0.000196477807004524	K00097	0,000666	0,000263
K00133	0.00114645	0.00095200	0.00104787205057929	K00099	0,000959	0,000904
K00134	0.00161884	0.00119547	0.00136703575987499	K00102	0,000536	0,000148
K00135	0.00016924	0.00050364	0.000232657980065475	K00111	0,000357	0,000362
K00145	0.00043447	0.00041495	0.000602385105685392	K00123	0,000455	0,000575
K00147	0.00032820	0.00036507	0.000403022933624446	K00128	0,00065	0,000444
K00161	0.00093853	0.00069472	0.000554397416606552	K00133	0,000764	0,000427
K00162	0.00094919	0.00070280	0.000561282031803408	K00134	0,001072	0,00097
K00164	0.00236140	0.00218937	0.00166414851526577	K00135	0,000422	0,000164
K00174	0.00117723	0.00061052	0.00144335914734842	K00140	0,000471	0,000263
K00175	0.00073717	0.00037695	0.000866854375242819	K00145	0,001365	0,001381
K00177	0.00032393	0.00015603	0.000322684367992312	K00147	0,000325	0,000247
K00179	0.00064712	0.00019541	0.000600908714637122	K00150	0,000179	0,000345
K00208	0.00102010	0.00076288	0.000900170535136897	K00161	0,000357	0,000181
K00215	0.00080104	0.00060893	0.000714964002199909	K00162	0,000357	0,000181
K00226	0.00051100	0.00056112	0.000805410586865844	K00169	0,000487	0,000197
K00228	0.00070425	0.00060130	0.000421311501745188	K00170	0,000487	0,000197
K00239	0.00195652	0.00183511	0.00187653760258874	K00171	0,000487	0,000197
K00240	0.00084660	0.00074750	0.00077039729462846	K00172	0,000487	0,000197
K00241	0.00029099	0.00030588	0.000387369679399641	K00174	0,000715	0,000789
K00243	0.00010531	0.00017479	0.000216813899424127	K00175	0,000715	0,000789

K00244	0.0004425	0.0002552	0.000215800146327654
K00248	0.0001097	0.0004944	0.000258496633932435
K00257	0.0002326	0.0009428	0.000663344100408111
K00259	0.0003448	0.0001926	0.000392062495610843
K00262	0.0002914	0.0004075	0.00065310597869302
K00265	0.0007217	0.0009864	0.000827860655266938
K00266	0.0010275	0.0008129	0.00111474859046651
K00274	0.0002876	0.0002241	0.000169455860060609
K00278	0.0006912	0.0006713	0.000940500527292008
K00281	0.0004454	0.0009518	0.00131118000346841
K00286	0.0002452	0.0002344	0.000264305075333453
K00290	0.0003717	0.0002031	0.000535663400506202
K00294	0.0001730	0.0001695	0.000406014558771071
K00297	0.0003626	0.0003486	0.000533069120241236
K00311	0.0001808	0.0003845	0.000361766182587875
K00318	0.0001557	0.0001102	0.000289814191087605
K00325	0.0001171	0.0002681	0.00018316769697811
K00330	0.0003442	0.0002801	0.000255326268646697
K00331	0.0005431	0.0004287	0.000411720072439471
K00332	0.0004809	0.0003936	0.000330068332020533
K00333	0.0012329	0.0009309	0.000836266140662199
K00334	0.0004986	0.0004589	0.000339754316624191
K00335	0.0013510	0.0013087	0.000916146079403479
K00336	0.0018978	0.0018019	0.00130337861434172
K00337	0.0010835	0.0008389	0.000806590830549886
K00338	0.0005294	0.0004118	0.000386722008424007
K00339	0.0004992	0.0004386	0.000390716828091948
K00340	0.0002389	0.0002024	0.000189652447190415
K00341	0.0016336	0.0015112	0.00131876328322448
K00342	0.0016156	0.0013055	0.00122236617867741
K00343	0.0012452	0.0010359	0.000968760309899676
K00344	0.0004776	0.0006643	0.000284680189796982
K00346	0.0001371	0.0002166	0.000415732174292951
K00347	0.0001308	0.0002320	0.000393310419055961
K00351	0.0001371	0.0002430	0.000404559040552797
K00359	0.0004635	0.0003771	0.000402573652384231
K00375	0.0002162	0.0005019	0.000130533533539224
K00378	0.0007903	0.0005551	0.000826187439970507
K00382	0.0030995	0.0028232	0.00259922133409587
K00384	0.0026083	0.0020069	0.00184664279263501
K00390	0.0001864	0.0002913	0.000219907317966123
K00404	0.0012648	0.0010772	0.000879171901437625
K00405	0.0005153	0.0004421	0.000358468797014985
K00406	0.0006096	0.0005719	0.000481424548337431
K00411	0.0003776	0.0003647	0.000276080065055227
K00412	0.0010800	0.0009639	0.000760393061844497
K00413	0.0006090	0.0005388	0.000423672115845061
K00425	0.0017690	0.0013062	0.0014397446082364
K00426	0.0013196	0.0009683	0.00104443229290199
K00432	0.0001049	0.0002711	0.000284085763926101

K00176	0,000682	0,000674
K00177	0,000682	0,000723
K00179	0,000179	0,00046
K00180	0,000179	0,00046
K00194	0,000146	0,000345
K00197	0,000146	0,000345
K00198	0,000146	0,000345
K00208	0,000227	0,000197
K00215	0,001056	0,001118
K00226	0,001056	0,000805
K00239	0,000244	0,000181
K00240	0,000244	0,000181
K00241	0,000195	0,000181
K00244	0,000179	0,000214
K00248	0,000634	0,000362
K00257	0,000585	0,000575
K00259	0,000357	0,00046
K00262	0,000146	0,000181
K00265	0,000682	0,000937
K00266	0,002112	0,002038
K00275	0,000309	0,000148
K00278	0,000699	0,000247
K00282	0,00065	0,000608
K00283	0,00065	0,000608
K00286	0,000504	0,00074
K00287	0,000179	0,000181
K00288	0,00013	0,000345
K00297	0,000845	0,000658
K00324	0,000276	0,000164
K00330	0,00013	0,00023
K00331	0,00013	0,000214
K00334	0,001024	0,00143
K00335	0,001072	0,001479
K00336	0,001024	0,001364
K00337	0,000146	0,00023
K00338	0,000114	0,000181
K00339	0,00013	0,00023
K00340	0,00013	0,000214
K00341	0,00013	0,00023
K00342	0,00013	0,00023
K00343	0,00013	0,00023
K00344	0,000325	0,000214
K00359	0,00026	0,000493
K00375	0,000325	0,000329
K00378	0,000309	0,000658
K00382	0,001186	0,000559
K00384	0,001121	0,0012
K00385	0,00013	0,000345
K00390	0,000244	0,000395
K00425	0,000276	0,000132

K00459	0.0001893	0.0004073	0.000245477633570632
K00507	0.0006558	0.0005746	0.00039094995748352
K00520	0.0002077	0.0001894	0.000000000000000000
K00525	0.0029409	0.0026623	0.00302179106798265
K00526	0.0010501	0.0010402	0.00091161011455323
K00527	0.0009250	0.0005212	0.000860189122482181
K00528	0.0002886	0.0003994	0.000527892333706428
K00540	0.0003640	0.0013556	0.000780387115421517
K00548	0.0010101	0.0011575	0.00151686299026372
K00549	0.0002269	0.0004170	0.000313499343118744
K00554	0.0010294	0.0007731	0.000835786001535456
K00558	0.0003069	0.0006403	0.000903567893556953
K00560	0.0001272	0.0003302	0.000371894389736926
K00566	0.0014935	0.0014096	0.00135643148220676
K00567	0.0001674	0.0002444	0.000233710769298975
K00568	0.0005852	0.0005472	0.000412156148703998
K00573	0.0004592	0.0004332	0.000311718487005167
K00575	0.0004972	0.0001517	0.00029117507593331
K00595	0.0003173	0.0001340	0.000342886042542574
K00599	0.0004971	0.0005796	0.000520389932279663
K00600	0.0016416	0.0012770	0.00142160243709878
K00602	0.0007180	0.0007464	0.000965353562672501
K00604	0.0012551	0.0010580	0.00105861399377155
K00605	0.0004411	0.0004169	0.000648385112631738
K00606	0.0002927	0.0003080	0.00044145290874519
K00609	0.0003906	0.0004617	0.000545016933520792
K00611	0.0002482	0.0002133	0.000220514600839196
K00615	0.0013107	0.0013412	0.00144745843759375
K00616	0.0008452	0.0007074	0.000795463315104618
K00620	0.0002910	0.0002212	0.000245192564497797
K00625	0.0001676	0.0003431	0.000393316858018758
K00626	0.0008541	0.0013651	0.000522887192308763
K00627	0.0012046	0.0013675	0.000874152271359279
K00639	0.0002765	0.0002630	0.000513852495301108
K00640	0.0003540	0.0003742	0.000557255465619964
K00641	0.0001171	0.0002583	0.000165958143670267
K00643	0.0010306	0.0007010	0.000610982003760419
K00645	0.0008822	0.0008138	0.000824394160521441
K00647	0.0003149	0.0003614	0.000567247154938385
K00648	0.0017062	0.0015835	0.00195859428184442
K00651	0.0001681	0.0002106	0.000356753951591485
K00652	0.0011585	0.0010160	0.000998803630437199
K00655	0.0008032	0.0007569	0.000869015470149467
K00656	0.0009110	0.0007513	0.000630911871233015
K00658	0.0010380	0.0009871	0.00072873768306392
K00661	0.0001973	0.0001163	0.000277965882896025
K00666	0.0004914	0.0006251	0.000433353337880607
K00674	0.0005201	0.0005286	0.000386471254171009
K00677	0.0008550	0.0007463	0.000777573578687672
K00680	0.0001723	0.0002565	0.00021775882748217

K00426	0,000276	0,000132
K00432	0,00039	0,000559
K00446	0,000211	0,000362
K00459	0,000195	0,000197
K00525	0,001007	0,000937
K00526	0,000244	0,000247
K00527	0,000796	0,000805
K00528	0,000796	0,000493
K00532	0,00013	0,000345
K00537	0,00013	0,000214
K00540	0,001105	0,00069
K00548	0,001186	0,001381
K00554	0,000829	0,000559
K00558	0,000585	0,000214
K00560	0,000227	0,000148
K00566	0,000975	0,000904
K00567	0,001007	0,000871
K00568	0,000309	0,000197
K00573	0,000179	0,000164
K00575	0,000195	0,000296
K00595	0,000536	0,000871
K00599	0,001316	0,001052
K00600	0,000861	0,000641
K00602	0,001901	0,001611
K00604	0,000975	0,000904
K00605	0,000991	0,000805
K00606	0,000764	0,000427
K00609	0,00078	0,000411
K00610	0,000162	0,000411
K00611	0,000877	0,00074
K00615	0,002128	0,002696
K00616	0,000292	0,000247
K00619	0,000666	0,000181
K00620	0,001024	0,00046
K00625	0,000796	0,000773
K00626	0,00065	0,000395
K00627	0,000406	0,000197
K00633	0,000292	0,000773
K00638	0,000179	0,00023
K00639	0,000195	0,000493
K00640	0,000536	0,000658
K00645	0,000959	0,000904
K00648	0,001154	0,001069
K00651	0,000211	0,000444
K00652	0,000601	0,000247
K00655	0,001154	0,001052
K00656	0,000211	0,000263
K00658	0,000179	0,000164
K00661	0,000227	0,000164
K00666	0,000292	0,000181

K00681	0.0001968(	0.0009406	0.000432865914852304
K00684	0.0001690!	0.0001391!	0.000143959507798807
K00688	0.0009750	0.0009161	0.00116564268641908
K00700	0.0008830(	0.0009180!	0.00142879891007186
K00703	0.0003068	0.0003688(	0.000382485710566576
K00705	0.0006167(	0.0006917:	0.00109054170324603
K00721	0.0010094!	0.0007708!	0.00129810326038853
K00748	0.0011321!	0.0009739	0.00101619893592138
K00754	0.0001206:	0.0001822!	0.000133886224488062
K00757	0.0001433:	0.0001447(	0.000314201980289397
K00760	0.0001751(	0.0002253:	0.000268916806765942
K00761	0.0002300!	0.0002738	0.000355984233900581
K00762	0.0002975:	0.0003318!	0.000397684890357352
K00763	0.0001477:	0.0005081:	0.00040672516228233
K00764	0.0006427!	0.0007186	0.000961309931540081
K00765	0.0002290	0.0002207	0.000267480016271869
K00766	0.0002684(	0.0002884	0.000329610714280281
K00767	0.0003913(	0.0004322(	0.000526496122315766
K00768	0.0003704!	0.0003305!	0.000483629699886695
K00773	0.0014553	0.0011952(	0.00124457468310039
K00782	0.0002275!	0.0001340(	0.000246407558899704
K00783	0.0001736:	0.0002122:	0.000258623955967928
K00784	0.0001475(	0.0001530:	0.000321762886992866
K00788	0.0002419!	0.0002389(	0.000170700672961569
K00789	0.0015480	0.0013168!	0.00134846086483758
K00790	0.0013074!	0.0011229:	0.00100890089736558
K00791	0.0009109:	0.0008162(	0.000993798234384241
K00793	0.0002587(	0.0003002(	0.000369525244169223
K00794	0.0002008:	0.0002250	0.000284872002660652
K00795	0.0003755!	0.0004339!	0.000300985345501915
K00796	0.0007697(	0.0006760!	0.000735767366133784
K00798	0.0001553:	0.0002806:	0.000283613274307401
K00799	0.0003823:	0.0007784	0.000243625112679487
K00800	0.0004665:	0.0006002:	0.000692549341796256
K00806	0.0008131(	0.0006925:	0.000738510739302865
K00812	0.0018457	0.0013569	0.00176806939392 9
K00817	0.0003911!	0.0004770:	0.00040452067102054
K00818	0.0004356:	0.0002185	0.000506467726303684
K00820	0.0022212(	0.0017999:	0.00159282299487148
K00826	0.0015624!	0.0010772:	0.00125414434699682
K00831	0.0001745:	0.0004474(	0.000516877501478128
K00833	0.0012887:	0.0010577!	0.00085110785669856
K00837	0.0002344!	0.0003327!	0.000361046903401758
K00845	0.0003686	0.0005045:	0.000606347322273408
K00847	0.0002202!	0.0003008	0.000395519742039959
K00848	0.0001310(	0.0001569!	0.000307686203945152
K00849	0.0002226	0.0003009!	0.000448394052402651
K00850	0.0002998:	0.0005189!	0.000653197055306963
K00854	0.0002456(	0.0004030!	0.000567844910022618
K00856	0.0002210!	0.0001012(	0.000118482642583218

K00674	0,000162	0,000181
K00675	0,000146	0,000362
K00677	0,000162	0,00023
K00681	0,000374	0,00023
K00684	0,000276	0,000395
K00688	0,000471	0,000707
K00690	0,000227	0,000395
K00697	0,000146	0,000164
K00700	0,000292	0,000197
K00703	0,000471	0,000822
K00705	0,000406	0,000674
K00721	0,00039	0,000164
K00748	0,000162	0,000279
K00754	0,000439	0,000674
K00756	0,000146	0,000444
K00757	0,000146	0,000214
K00759	0,000471	0,000723
K00760	0,000195	0,000214
K00761	0,000455	0,00069
K00762	0,000959	0,000904
K00763	0,000325	0,000378
K00764	0,000942	0,000756
K00765	0,000764	0,000362
K00766	0,00091	0,000641
K00767	0,000715	0,000247
K00768	0,000227	0,000477
K00772	0,000715	0,00051
K00773	0,00026	0,000427
K00783	0,000114	0,000214
K00784	0,000325	0,000608
K00788	0,000747	0,000329
K00789	0,000975	0,000904
K00790	0,000975	0,000921
K00791	0,001056	0,001085
K00793	0,000747	0,000378
K00794	0,00078	0,000378
K00796	0,000764	0,000345
K00798	0,000845	0,000658
K00799	0,000715	0,000362
K00800	0,000959	0,000838
K00806	0,000829	0,000559
K00812	0,000829	0,000477
K00817	0,001625	0,001101
K00820	0,000926	0,000789
K00821	0,000926	0,001069
K00826	0,001024	0,000773
K00830	0,00039	0,000181
K00831	0,000341	0,000542
K00833	0,000666	0,000542
K00844	0,000227	0,000542

K00858	0.0008329	0.0006829	0.000736719954277799
K00859	0.0004071	0.0004847	0.000430717761195283
K00864	0.0002023	0.0004732	0.000103354496216331
K00873	0.0006080	0.0007225	0.000830235138770827
K00874	0.0002602	0.0003662	0.000615036642424279
K00876	0.0006331	0.0005826	0.00083141477651771
K00878	0.0003312	0.0001544	0.000180722997990548
K00891	0.0004109	0.0003517	0.000409741725378118
K00895	0.0006297	0.0002400	0.000775407633168572
K00912	0.0009545	0.0008499	0.000882313127958628
K00919	0.0009738	0.0008213	0.000855522780000016
K00925	0.0005648	0.0006641	0.000990109665504159
K00927	0.0015716	0.0012802	0.00135232092356245
K00928	0.0022997	0.0017701	0.00190575626043089
K00929	0.0002003	0.0001278	0.000200762637299291
K00930	0.0003525	0.0003349	0.000479485919364103
K00931	0.0002784	0.0003204	0.00034846080333633
K00936	0.0045126	0.0015283	0.00284945680032259
K00937	0.0002888	0.0007751	0.000887656915385328
K00939	0.0006826	0.0005739	0.000596968963804744
K00940	0.0005106	0.0003803	0.000374238246019755
K00941	0.0002224	0.0002554	0.000177991631329546
K00942	0.0008547	0.0006905	0.000711857562573116
K00943	0.0006857	0.0005444	0.000450443848980641
K00945	0.0007886	0.0006637	0.000726666373856876
K00946	0.0005707	0.0005091	0.000687084433511708
K00948	0.0004158	0.0004672	0.000564467593104432
K00950	0.0004165	0.0003778	0.000434716404887324
K00951	0.0027637	0.0025048	0.00286046147398808
K00954	0.0006164	0.0005085	0.000525971726980784
K00962	0.0025672	0.0021136	0.00226070330197069
K00963	0.0010408	0.0008045	0.000655166123530787
K00969	0.0005886	0.0005076	0.000537287588519506
K00970	0.0010667	0.0008892	0.000988868158871662
K00971	0.0003074	0.0002516	0.000462683680376732
K00973	0.0004837	0.0004767	0.000750697058690567
K00974	0.0007269	0.0004531	0.000553327760771376
K00978	0.0001466	0.0001060	0.000155912339856741
K00979	0.0008754	0.0007127	0.000785450206400873
K00981	0.0006714	0.0006528	0.00060049229383274
K00983	0.0002079	0.0001073	0.000281844047125428
K00989	0.0005621	0.0006020	0.000397352262410604
K00990	0.0003314	0.0004783	0.000386376803296693
K00991	0.0002597	0.0003993	0.000404666290806854
K00995	0.0006054	0.0005499	0.000498068014771795
K00997	0.0004597	0.0003621	0.000295760450710826
K00998	0.0007932	0.0006828	0.000670322414133632
K01000	0.0014305	0.0011666	0.00127062438234123
K01006	0.0024070	0.0020458	0.00219383858256588
K01007	0.0024231	0.0005709	0.0013871586136168

K00845	0,000552	0,000378
K00847	0,00039	0,000575
K00849	0,00052	0,001069
K00850	0,000747	0,000378
K00852	0,000227	0,00023
K00854	0,000601	0,000904
K00858	0,001007	0,000904
K00859	0,000942	0,000888
K00864	0,000471	0,000707
K00868	0,000341	0,000592
K00872	0,000634	0,000214
K00873	0,001007	0,000756
K00874	0,001154	0,001151
K00876	0,000374	0,000674
K00878	0,00013	0,000148
K00882	0,000227	0,00046
K00891	0,000796	0,000329
K00895	0,000682	0,000477
K00912	0,000114	0,00023
K00919	0,000959	0,000871
K00925	0,000812	0,000493
K00927	0,000975	0,000904
K00928	0,000812	0,000477
K00930	0,001024	0,000526
K00931	0,000487	0,00074
K00936	0,001024	0,000559
K00939	0,000991	0,000904
K00940	0,000877	0,000658
K00941	0,00052	0,000444
K00942	0,000764	0,000411
K00943	0,000942	0,000855
K00945	0,000309	0,000427
K00946	0,000699	0,000247
K00948	0,000959	0,000904
K00949	0,000211	0,00051
K00950	0,000796	0,000411
K00951	0,000552	0,000855
K00954	0,001316	0,000674
K00956	0,000162	0,000395
K00957	0,000195	0,000444
K00962	0,000829	0,000559
K00963	0,00026	0,000181
K00969	0,000975	0,000921
K00970	0,001072	0,001003
K00973	0,000374	0,000542
K00974	0,000276	0,000411
K00975	0,00039	0,000559
K00978	0,000211	0,000378
K00979	0,00013	0,00023
K00981	0,000861	0,000559

K01011	0.0001029	0.0002541	0.000000000000000000
K01012	0.0012161	0.0009703	0.000794019893340201
K01042	0.0003395	0.0001287	0.00016530140637603
K01056	0.0006925	0.0005859	0.000604993865534031
K01057	0.0001809	0.0001682	0.000295354264470683
K01067	0.0001642	0.0002967	0.000391456232933022
K01069	0.0001953	0.0003462	0.000267516805744864
K01077	0.0002634	0.0002795	0.00040674930539027
K01079	0.0002196	0.0002258	0.000322393501646307
K01081	0.0001749	0.0001961	0.000201360499396704
K01085	0.0001103	0.0001288	0.000258479264984367
K01091	0.0005748	0.0006886	0.000692565984849095
K01092	0.0008269	0.0007160	0.000659899567939717
K01095	0.0004110	0.0003564	0.000315039482901433
K01126	0.0003128	0.0005148	0.000411460355772839
K01127	0.0004216	0.0002854	0.000249432419034621
K01129	0.0009784	0.0008382	0.000937257375208297
K01130	0.0003870	0.0003470	0.000672026123276292
K01142	0.0009614	0.0009292	0.000776535971497848
K01144	0.0001661	0.0001725	0.000390841840917947
K01147	0.0004018	0.0003475	0.000359710834698586
K01151	0.0002337	0.0001330	0.000267759390073092
K01153	0.0013432	0.0015082	0.00204826550864317
K01154	0.0005399	0.0005842	0.000846971973405894
K01159	0.0004944	0.0004387	0.000484830234216961
K01163	0.0002843	0.0001248	0.000291745703394846
K01181	0.0001793	0.0001828	0.000416359882829536
K01186	0.0001325	0.0001242	0.000308269992058993
K01187	0.0003716	0.0005142	0.000878698935046369
K01190	0.0014198	0.0014417	0.00337211641122523
K01200	0.0001089	0.0001063	0.000257930793048053
K01201	0.0003940	0.0003831	0.000932618457242375
K01206	0.0003456	0.0003768	0.000818009588769529
K01207	0.0009975	0.0008178	0.00073178247757461
K01209	0.0001898	0.0001925	0.000446768054320844
K01235	0.0001914	0.0001912	0.000453105985252273
K01241	0.0001220	0.0002386	0.000290861860510602
K01246	0.0001052	0.0001398	0.000152323186131563
K01251	0.0006044	0.0003915	0.000724623055635773
K01255	0.0012503	0.0011776	0.000847464685230475
K01258	0.0001958	0.0003766	0.000460809857347667
K01262	0.0012887	0.0012872	0.00129779613744161
K01265	0.0010625	0.0009222	0.000971877670713522
K01270	0.0004496	0.0007767	0.00107152012521518
K01277	0.0002957	0.0002806	0.000699909524705327
K01278	0.0005201	0.0005121	0.0012185960482303
K01284	0.0002369	0.0003354	0.00056352775015974
K01286	0.0003919	0.0002615	0.000229929559582675
K01297	0.0003735	0.0004274	0.000394894417118019
K01322	0.0002187	0.0003987	0.000692102130223012

K00989	0,000211	0,000181
K00991	0,000666	0,000411
K00995	0,000926	0,000707
K00997	0,000894	0,000805
K00998	0,000617	0,000362
K01000	0,000959	0,000904
K01002	0,00013	0,000362
K01006	0,000341	0,000855
K01007	0,000292	0,000148
K01008	0,000341	0,000608
K01011	0,000715	0,000279
K01012	0,000926	0,000986
K01042	0,000179	0,000493
K01056	0,000829	0,000559
K01061	0,000309	0,000395
K01069	0,000715	0,000247
K01077	0,00026	0,000641
K01081	0,000357	0,00046
K01089	0,000552	0,000247
K01091	0,000959	0,001332
K01092	0,001105	0,000773
K01095	0,000617	0,000329
K01104	0,000585	0,00069
K01119	0,000244	0,000526
K01126	0,000536	0,000674
K01129	0,000309	0,000197
K01142	0,001137	0,00097
K01147	0,000162	0,000164
K01153	0,001267	0,001611
K01154	0,001332	0,001693
K01155	0,000114	0,000148
K01159	0,000959	0,000871
K01163	0,000244	0,000575
K01179	0,00091	0,000888
K01183	0,000162	0,000197
K01187	0,000471	0,000395
K01190	0,000309	0,000773
K01191	0,000309	0,000444
K01192	0,000357	0,000477
K01198	0,000211	0,000378
K01201	0,000114	0,000247
K01206	0,000162	0,000214
K01209	0,000325	0,000559
K01222	0,000146	0,000378
K01223	0,000422	0,000904
K01238	0,000162	0,000427
K01243	0,000292	0,000542
K01246	0,000341	0,000526
K01247	0,000276	0,000444
K01251	0,000747	0,000329

K01338	0.0038902	0.0025193	0.00300984895811803
K01354	0.0014568	0.0011464	0.00120256079314481
K01358	0.0008360	0.0007041	0.000728418188722736
K01362	0.0022294	0.0018270	0.00166024002918874
K01372	0.0001461	0.0001385	0.000345844006818397
K01409	0.0013227	0.0011195	0.00115098947634227
K01412	0.0001503	0.0001050	0.000103193309321912
K01417	0.0001828	0.0003156	0.000194187330128141
K01419	0.0006086	0.0004071	0.000346627900616067
K01420	0.0001654	0.0001827	0.000150129257004901
K01422	0.0001520	0.0002545	0.000185057460074486
K01423	0.0001012	0.0001679	0.000000000000000000
K01424	0.0002800	0.0004455	0.000524845997788853
K01425	0.0001111	0.0002294	0.000222147936812438
K01433	0.0001687	0.0001802	0.000176886456378646
K01438	0.0001574	0.0002882	0.000374575827086763
K01439	0.0011763	0.0009934	0.00078873325119767
K01448	0.0004970	0.0006150	0.000699190936634638
K01451	0.0001237	0.0003505	0.000105342241075372
K01462	0.0005843	0.0005334	0.000521021877165158
K01465	0.0005822	0.0006923	0.00076510027978486
K01468	0.0001539	0.0003572	0.000312051879978318
K01485	0.0002328	0.0002508	0.000204812952389954
K01486	0.0002573	0.0001388	0.000265536487129135
K01491	0.0011148	0.0009108	0.000956647901705676
K01493	0.0003632	0.0002469	0.000317263713744631
K01495	0.0005586	0.0005451	0.000491178453916624
K01507	0.0005750	0.0005368	0.000552764717918634
K01520	0.0006056	0.0004929	0.000510422155513745
K01524	0.0002203	0.0006448	0.00036674947799339
K01531	0.0001322	0.0002126	0.000000000000000000
K01533	0.0023779	0.0023464	0.00199931590787645
K01534	0.0002975	0.0005397	0.000162474382013304
K01537	0.0003128	0.0003325	0.000155672287224078
K01546	0.0002370	0.0002470	0.000297827814061528
K01547	0.0002878	0.0003003	0.000367150097560098
K01552	0.0001513	0.0001097	0.000111963923771172
K01572	0.0001981	0.0003686	0.000447925079915365
K01579	0.0001021	0.0001300	0.000191430183318979
K01585	0.0004115	0.0003118	0.000510108865982461
K01586	0.0009358	0.0007234	0.000937770569734796
K01588	0.0002088	0.0002265	0.000285428580729138
K01591	0.0003093	0.0003572	0.000459482071786599
K01599	0.0007023	0.0007064	0.000584530032341936
K01609	0.0002201	0.0002038	0.000255106781331592
K01610	0.0002956	0.0004694	0.000627121227576013
K01613	0.0008997	0.0007603	0.000742860841743272
K01619	0.0001880	0.0001717	0.000305664065293609
K01620	0.0002002	0.0002906	0.000211451198912606
K01624	0.0004346	0.0005013	0.000723293483088567

K01255	0,000309	0,000214
K01258	0,000179	0,00051
K01262	0,00091	0,000592
K01265	0,001186	0,000937
K01269	0,000796	0,000871
K01270	0,000114	0,000263
K01271	0,000341	0,000773
K01273	0,000276	0,000493
K01304	0,000325	0,000493
K01338	0,000926	0,000937
K01356	0,000357	0,000477
K01358	0,001397	0,001414
K01362	0,000682	0,000953
K01392	0,000569	0,000132
K01409	0,001462	0,001052
K01417	0,000406	0,000542
K01419	0,000162	0,000296
K01424	0,000195	0,000247
K01436	0,000276	0,000674
K01439	0,000585	0,001184
K01443	0,000374	0,000559
K01448	0,000861	0,000575
K01462	0,00117	0,00097
K01464	0,000292	0,000444
K01465	0,001072	0,000937
K01470	0,000374	0,000477
K01486	0,000146	0,000247
K01488	0,00026	0,000181
K01489	0,00039	0,000658
K01491	0,001982	0,001825
K01493	0,000796	0,000789
K01494	0,00065	0,000247
K01495	0,000829	0,000312
K01496	0,000682	0,000214
K01497	0,000601	0,000197
K01506	0,00013	0,000345
K01507	0,001284	0,001743
K01512	0,000374	0,000592
K01515	0,00026	0,000197
K01516	0,000829	0,000526
K01520	0,000406	0,000822
K01524	0,000617	0,000526
K01529	0,000114	0,000214
K01533	0,00078	0,000838
K01534	0,000585	0,001184
K01537	0,000195	0,000181
K01572	0,000309	0,000888
K01579	0,000601	0,000214
K01581	0,000244	0,000575
K01586	0,000975	0,000773

K01627	0.0010522	0.0008952	0.000908012431248044
K01633	0.0001945	0.0002495	0.000241472053759456
K01638	0.0003587	0.0002156	0.000188728226665792
K01647	0.0014410	0.0012791	0.00108274809711583
K01649	0.0008762	0.0007425	0.00106422009626209
K01652	0.0009027	0.0010115	0.000772519258979329
K01653	0.0002256	0.0001373	0.000201400957915604
K01654	0.0004628	0.0002318	0.000466442453036429
K01657	0.0003613	0.0004644	0.000480999211706607
K01658	0.0002008	0.0002333	0.000317723512639713
K01661	0.0002582	0.0002306	0.000525942010885427
K01662	0.0023198	0.0020206	0.00203034046079296
K01665	0.0002320	0.0002721	0.000346469587436443
K01667	0.0001205	0.0002181	0.000206093041069844
K01669	0.0001940	0.0002915	0.000209440821690661
K01673	0.0005976	0.0005042	0.000449212443961402
K01676	0.0002662	0.0005854	0.000769444392223896
K01679	0.0015291	0.0011466	0.00100422080463468
K01681	0.0028675	0.0026983	0.00212271502126889
K01685	0.0001223	0.0001781	0.000000000000000000
K01686	0.0001367	0.0001659	0.000321240254430241
K01687	0.0004646	0.0008084	0.000636413699490534
K01689	0.0016057	0.0013338	0.0013995776687608
K01693	0.0001178	0.0001255	0.000136246730195194
K01695	0.0002258	0.0002279	0.000267047405119226
K01696	0.0005188	0.0004391	0.000483341236766086
K01698	0.0010714	0.0008918	0.000802762412353862
K01703	0.0004880	0.0006431	0.00079350779038053
K01704	0.0001871	0.0002640	0.000320766904594152
K01709	0.0002245	0.0001398	0.000238299151448775
K01710	0.0009640	0.0009545	0.00139620362717793
K01711	0.0004836	0.0003518	0.000559520700861544
K01712	0.0002095	0.0005651	0.000496928444483734
K01714	0.0010752	0.0010001	0.000969640336065773
K01719	0.0003071	0.0003635	0.000459746155127158
K01733	0.0004138	0.0004094	0.000471499328374103
K01735	0.0001585	0.0003948	0.000427542663281469
K01736	0.0003962	0.0004868	0.000616244627464861
K01737	0.0004568	0.0003299	0.000321165763682726
K01738	0.0004702	0.0007149	0.000805627965831743
K01740	0.0005267	0.0005919	0.00089932655102651
K01744	0.0004725	0.0002574	0.000349683889957223
K01745	0.0002966	0.0006622	0.000704572558007514
K01749	0.0009883	0.0008032	0.0007182816262872
K01752	0.0005193	0.0005961	0.000748321997441289
K01755	0.0005629	0.0005772	0.000786144737213445
K01756	0.0005523	0.0006561	0.00079523324698148
K01768	0.0002182	0.0001095	0.000167210006087105
K01770	0.0002703	0.0003464	0.000332572164539157
K01772	0.0008735	0.0008135	0.000668295825012489

K01588	0,000959	0,000871
K01591	0,000894	0,000756
K01596	0,000796	0,000641
K01598	0,000617	0,000493
K01599	0,00065	0,001332
K01607	0,000487	0,000838
K01609	0,000894	0,000658
K01613	0,000617	0,000362
K01619	0,000487	0,000658
K01620	0,000422	0,000723
K01623	0,00013	0,000164
K01624	0,000959	0,000904
K01625	0,001755	0,00194
K01626	0,000211	0,000148
K01627	0,00013	0,00023
K01628	0,000211	0,000247
K01633	0,000244	0,000148
K01643	0,00052	0,001348
K01644	0,001056	0,001463
K01646	0,000292	0,000789
K01647	0,000699	0,000723
K01649	0,001284	0,001299
K01652	0,00143	0,001036
K01653	0,000894	0,000658
K01657	0,000894	0,000658
K01658	0,000764	0,000608
K01662	0,001007	0,000921
K01666	0,000146	0,000427
K01669	0,000341	0,000247
K01673	0,000341	0,000197
K01677	0,00065	0,000575
K01678	0,00065	0,00051
K01679	0,000195	0,000197
K01681	0,00052	0,000921
K01684	0,000146	0,000411
K01686	0,000731	0,000773
K01687	0,001137	0,001036
K01689	0,000991	0,000904
K01692	0,001267	0,000444
K01693	0,000731	0,000296
K01695	0,000894	0,000658
K01696	0,000894	0,000707
K01698	0,000374	0,000477
K01703	0,001868	0,001644
K01704	0,001933	0,001644
K01709	0,000211	0,000378
K01710	0,000942	0,001512
K01711	0,000487	0,00051
K01714	0,000942	0,000592
K01733	0,001121	0,001134

K01775	0.0013172	0.0012272	0.00148103357343666
K01776	0.0003286	0.0003679	0.000492087031825683
K01778	0.0002712	0.0002595	0.000311862672147459
K01783	0.0006688	0.0005848	0.000624116359278931
K01784	0.0010756	0.0008473	0.00107665673144813
K01785	0.0004244	0.0005243	0.000987919812705925
K01787	0.0001141	0.0001081	0.00027011276262362
K01790	0.0003310	0.0003214	0.000445590336416014
K01791	0.0003912	0.0004107	0.000780076042867944
K01802	0.0003320	0.0002501	0.00024202309430141
K01803	0.0008783	0.0007373	0.00078050625385978
K01805	0.0002114	0.0002568	0.000500462732779804
K01808	0.0005972	0.0004142	0.000455825844799651
K01809	0.0002271	0.0003049	0.000537201095594983
K01810	0.0010058	0.0011164	0.00123690324556021
K01811	0.0004330	0.0004826	0.00102472688952111
K01812	0.0002158	0.0002477	0.000508080438575879
K01813	0.0001245	0.0001469	0.000294661277071354
K01814	0.0001929	0.0002231	0.000237266276984277
K01816	0.0001048	0.0001869	0.000210314954779467
K01817	0.0004267	0.0002844	0.00034089072476284
K01818	0.0001867	0.0001932	0.000441978731657274
K01834	0.0001091	0.0002188	0.000110039757123695
K01835	0.0002943	0.0002663	0.000174193691484637
K01840	0.0012836	0.0010590	0.001563196880687
K01843	0.0003619	0.0003314	0.00038739516930036
K01845	0.0003992	0.0005691	0.000447962282193711
K01847	0.0006019	0.0006613	0.00142434998525871
K01866	0.0016286	0.0013245	0.00141965253134515
K01867	0.0012301	0.0010550	0.00106782246080007
K01868	0.0024552	0.0020203	0.00211784244863663
K01869	0.0033985	0.0028243	0.00297861880860543
K01870	0.0038513	0.0031736	0.00341095728884594
K01872	0.0029974	0.0025264	0.00264950248448093
K01873	0.0027056	0.0023762	0.00253820761895057
K01874	0.0020855	0.0018123	0.00195114438373723
K01875	0.0015599	0.0012902	0.00135465874587169
K01876	0.0024087	0.0019564	0.00204680999926574
K01878	0.0009753	0.0007967	0.000630781162911797
K01879	0.0016774	0.0014112	0.00112009549388933
K01880	0.0002462	0.0002338	0.000582821902286797
K01881	0.0018641	0.0015693	0.00163463919729268
K01883	0.0018295	0.0013744	0.00156615286993928
K01885	0.0028273	0.0021047	0.00218215403620658
K01886	0.0007205	0.0006179	0.00102042492780614
K01887	0.0020637	0.0017236	0.00184159575467491
K01889	0.0013585	0.0011051	0.00114451490212826
K01890	0.0025132	0.0021767	0.00232698791605711
K01892	0.0014140	0.0012063	0.00130305175775353
K01893	0.0005814	0.0004418	0.000706326384575629

K01735	0,000991	0,000822
K01736	0,000829	0,00046
K01737	0,000666	0,000296
K01738	0,00052	0,000641
K01739	0,00039	0,000723
K01740	0,00052	0,000427
K01744	0,000276	0,000395
K01745	0,000146	0,000181
K01749	0,000374	0,000477
K01750	0,000244	0,000197
K01752	0,000276	0,000477
K01753	0,000162	0,000378
K01754	0,00039	0,000181
K01755	0,00078	0,000378
K01756	0,000829	0,000559
K01759	0,000309	0,000493
K01760	0,000211	0,000411
K01761	0,000162	0,000427
K01768	0,001137	0,001743
K01770	0,000666	0,000395
K01772	0,000276	0,000164
K01775	0,001072	0,000921
K01776	0,000926	0,000773
K01778	0,000764	0,000411
K01783	0,000975	0,000904
K01784	0,00182	0,001693
K01785	0,000487	0,000723
K01790	0,000439	0,000559
K01791	0,000211	0,00023
K01802	0,00078	0,000723
K01803	0,001056	0,000921
K01805	0,000292	0,000427
K01807	0,000195	0,000214
K01808	0,000959	0,000838
K01809	0,000455	0,000625
K01810	0,000926	0,000542
K01811	0,000162	0,000214
K01812	0,000715	0,000707
K01814	0,000666	0,000362
K01817	0,000764	0,000296
K01834	0,001706	0,001677
K01835	0,000357	0,000559
K01838	0,000601	0,000279
K01840	0,000959	0,000608
K01841	0,000211	0,000493
K01846	0,000276	0,000707
K01847	0,000114	0,000164
K01854	0,000244	0,000411
K01866	0,000959	0,000904
K01867	0,00104	0,000904

K01895	0.0013263	0.0007430	0.00116994140052255
K01897	0.0016914	0.0020319	0.00283072182591419
K01902	0.0011325	0.0007695	0.000799751084963603
K01903	0.0011515	0.0010342	0.000895914979761128
K01911	0.0002737	0.0002544	0.000555736533378946
K01912	0.0015218	0.0003525	0.00106080072384957
K01914	0.0001574	0.0002514	0.000372688286383836
K01915	0.0015017	0.0010479	0.00153995825747914
K01916	0.0005740	0.0005424	0.000407412869078803
K01918	0.0002734	0.0003032	0.000429137719975815
K01919	0.0004733	0.0007284	0.000571396439898558
K01920	0.0006571	0.0006244	0.00047455763564001
K01921	0.0013248	0.0010015	0.00107491236730693
K01923	0.0003830	0.0004007	0.000549880690872115
K01924	0.0017146	0.0014229	0.00160190490802575
K01925	0.0014975	0.0012776	0.00134858185623585
K01926	0.0002623	0.0001135	0.000318782725045333
K01928	0.0016032	0.0013459	0.00144511749907921
K01929	0.0013571	0.0011678	0.00123661636265306
K01933	0.0004632	0.0005088	0.000673760255324464
K01934	0.0004638	0.0003805	0.000437108635501556
K01935	0.0005862	0.0004957	0.000390367510549589
K01937	0.0021928	0.0016627	0.00184920434388882
K01938	0.0001896	0.0002920	0.000236008017746219
K01939	0.0005387	0.0006178	0.000759193953377935
K01940	0.0005216	0.0005079	0.000753785747326605
K01945	0.0005448	0.0006084	0.000765884481035666
K01950	0.0010789	0.0008166	0.00113549200945876
K01951	0.0007142	0.0008136	0.000983692414503837
K01952	0.0015370	0.0017860	0.00221486617980596
K01953	0.0009894	0.0006014	0.000924767056387677
K01955	0.0014794	0.0017601	0.00218927928014951
K01956	0.0003482	0.0004497	0.000376054606647103
K01958	0.0008132	0.0001524	0.000401262154000492
K01960	0.0002753	0.0002647	0.00062188016169921
K01961	0.0004908	0.0006703	0.000521728689970316
K01965	0.0008910	0.0006788	0.000527163239091087
K01966	0.0017761	0.0014140	0.00170965227303637
K01968	0.0001044	0.0002207	0.000000000000000000
K01972	0.0025895	0.0022059	0.00228075156744591
K01989	0.0005193	0.0004284	0.000293309052885334
K01990	0.0008262	0.0007815	0.00142684328072321
K01991	0.0002433	0.0003342	0.000453617026494508
K01992	0.0002930	0.0004176	0.000385538353543077
K01993	0.0004859	0.0003818	0.000691312566466299
K01995	0.0008197	0.0006766	0.000405485915588797
K01996	0.0007449	0.0006736	0.00037604985452541
K01997	0.0009713	0.0008742	0.000481855798600065
K01998	0.0010271	0.0008167	0.000504357520817162
K01999	0.0013965	0.0015234	0.000752165985357644

K01868	0,000959	0,000904
K01869	0,000959	0,000904
K01870	0,000959	0,000904
K01872	0,000959	0,000904
K01873	0,000942	0,000871
K01874	0,000959	0,000904
K01875	0,000959	0,000904
K01876	0,000959	0,000904
K01878	0,00065	0,000312
K01879	0,00065	0,000279
K01880	0,000325	0,000592
K01881	0,000975	0,00097
K01883	0,000845	0,000559
K01885	0,00104	0,00097
K01886	0,000211	0,000477
K01887	0,000959	0,000904
K01889	0,000829	0,000559
K01890	0,000959	0,000904
K01892	0,000959	0,000904
K01893	0,000309	0,000658
K01895	0,00078	0,001052
K01897	0,001121	0,001677
K01902	0,000276	0,00023
K01903	0,00026	0,00023
K01906	0,000162	0,000411
K01910	0,000211	0,00051
K01912	0,000666	0,001036
K01915	0,000959	0,001019
K01918	0,00078	0,000345
K01921	0,000991	0,000986
K01922	0,000617	0,000493
K01923	0,000975	0,000904
K01924	0,000959	0,000904
K01925	0,000959	0,000904
K01926	0,000244	0,000625
K01928	0,000812	0,000559
K01929	0,000959	0,000904
K01933	0,000959	0,000871
K01934	0,000942	0,000789
K01935	0,000682	0,000526
K01937	0,000959	0,000888
K01938	0,000325	0,000756
K01939	0,000845	0,000411
K01940	0,000764	0,000362
K01945	0,000764	0,000378
K01950	0,00091	0,000723
K01951	0,001121	0,000937
K01952	0,001836	0,001233
K01953	0,000357	0,000575
K01955	0,001056	0,001101

K02002	0.0002182	0.0001208	0.000119897910006932
K02003	0.0009542	0.0007608	0.000992633219953793
K02004	0.0024485	0.0022119	0.00329338382606702
K02005	0.0006362	0.0005222	0.00110750426545351
K02006	0.0003779	0.0002513	0.000264885107378977
K02008	0.0002515	0.0001245	0.000180744027083107
K02010	0.0001065	0.0004245	0.000167688917154054
K02011	0.0001725	0.0007096	0.000231464252595309
K02012	0.0001272	0.0004431	0.000151517152653228
K02013	0.0005161	0.0007898	0.000669219213832324
K02014	0.0058230	0.0080586	0.0133977527905723
K02015	0.0008060	0.0012670	0.00119342803432113
K02016	0.0007353	0.0009860	0.00115424705508757
K02017	0.0001619	0.0001416	0.000123944465741756
K02018	0.0002236	0.0001428	0.000176267902588348
K02019	0.0003284	0.0001069	0.000214335438931255
K02020	0.0003020	0.0002074	0.000213507727323569
K02022	0.0004414	0.0003192	0.000288350337956941
K02023	0.0001287	0.0001704	0.000000000000000000
K02025	0.0002525	0.0002516	0.000169798740221626
K02026	0.0001321	0.0001648	0.000100270527250613
K02027	0.0002132	0.0003088	0.0001430367941353
K02028	0.0013235	0.0007771	0.000779592737985154
K02029	0.0018566	0.0008674	0.00103673418941394
K02030	0.0015370	0.0009763	0.000893807240256559
K02031	0.0005172	0.0006510	0.000260261140273813
K02032	0.0004820	0.0006825	0.000246397416928491
K02033	0.0005243	0.0008612	0.000271105652132536
K02034	0.0004418	0.0007478	0.000218373134987355
K02035	0.0011834	0.0018690	0.000620652351222963
K02036	0.0003271	0.0002329	0.000315299177499016
K02037	0.0003761	0.0002942	0.000372034351792904
K02038	0.0003851	0.0002672	0.000377124355276304
K02039	0.0001737	0.0001363	0.000130446897775538
K02040	0.0004745	0.0004257	0.000549991772520884
K02042	0.0002133	0.0001447	0.000102736116548667
K02044	0.0003328	0.0001485	0.00015981434528218
K02049	0.0002525	0.0003959	0.000174746443008667
K02050	0.0002529	0.0003848	0.000151441036855772
K02051	0.0001406	0.0003637	0.000000000000000000
K02052	0.0001603	0.0001994	0.000000000000000000
K02056	0.0005192	0.0004889	0.000248727742689927
K02057	0.0007092	0.0005478	0.00035770334560251
K02065	0.0011674	0.0007984	0.000920959364516244
K02066	0.0011327	0.0007753	0.000889101469620279
K02067	0.0002288	0.0002161	0.000161248882803285
K02078	0.0003495	0.0003158	0.0003618608845305
K02108	0.0010112	0.0008288	0.00089858572469471
K02109	0.0004767	0.0003533	0.000423410767595056
K02110	0.0003110	0.0002408	0.000256393263599033

K01956	0,000747	0,000329
K01961	0,001641	0,001397
K01962	0,000894	0,000921
K01963	0,00091	0,000953
K01972	0,000829	0,000575
K01989	0,000244	0,000526
K01990	0,001592	0,0012
K01991	0,000309	0,00023
K01992	0,001316	0,00166
K01993	0,000601	0,000296
K01995	0,001267	0,001479
K01996	0,001332	0,001644
K01997	0,001024	0,000805
K01998	0,001154	0,001151
K01999	0,001413	0,001249
K02000	0,000211	0,000148
K02001	0,000211	0,000181
K02002	0,00026	0,000247
K02005	0,000325	0,000296
K02006	0,001202	0,00189
K02007	0,000699	0,000559
K02008	0,000991	0,001414
K02010	0,000715	0,001512
K02011	0,001121	0,002564
K02012	0,001154	0,002564
K02013	0,002226	0,003123
K02014	0,001121	0,001907
K02015	0,002453	0,003025
K02016	0,002258	0,00309
K02018	0,000325	0,000279
K02020	0,000406	0,000493
K02023	0,000406	0,000181
K02025	0,003379	0,004422
K02026	0,003623	0,004932
K02027	0,002778	0,003271
K02028	0,000959	0,001743
K02029	0,001641	0,003107
K02030	0,001316	0,002055
K02031	0,002356	0,004997
K02032	0,003266	0,00508
K02033	0,003623	0,00577
K02034	0,003314	0,005293
K02035	0,004094	0,006723
K02036	0,000829	0,000378
K02037	0,00091	0,000427
K02038	0,00091	0,000427
K02039	0,000764	0,000427
K02040	0,001137	0,00069
K02042	0,000325	0,000263
K02045	0,000211	0,000395

K02111	0.0019772	0.0015993	0.0016946163409807
K02112	0.0020029	0.0015904	0.00169451028373095
K02113	0.0003432	0.0002987	0.00030540263855866
K02114	0.0001948	0.0001827	0.000192229299183244
K02115	0.0008315	0.0006978	0.000738798491332931
K02117	0.0001793	0.0003110	0.000424662143206629
K02118	0.0001297	0.0002401	0.000309209937019669
K02123	0.0001089	0.0001735	0.000257822684660044
K02169	0.0002363	0.0003663	0.000205670865308113
K02188	0.0003947	0.0003442	0.000593766983853823
K02189	0.0001898	0.0001810	0.000198138849524974
K02190	0.0003364	0.0001491	0.000286904166721866
K02193	0.0005005	0.0003471	0.000290349469975399
K02195	0.0006099	0.0003932	0.000345509984133192
K02197	0.0004726	0.0002994	0.000268989744147387
K02198	0.0014029	0.0008820	0.000795212859313816
K02200	0.0001873	0.0002329	0.000111630940682424
K02224	0.0007593	0.0004240	0.000752329377912016
K02227	0.0002708	0.0003226	0.000350432998392696
K02230	0.0004211	0.0005113	0.000996264840085407
K02231	0.0001612	0.0001678	0.000227377910951693
K02232	0.0005152	0.0006456	0.000698983676618767
K02233	0.0002059	0.0002045	0.000285887237039549
K02238	0.0005692	0.0006524	0.000712433407912493
K02274	0.0002602	0.0003221	0.000295650147153886
K02275	0.0001396	0.0002052	0.000129125107296257
K02313	0.0015380	0.0010315	0.00135292565740591
K02314	0.0018120	0.0014755	0.00154433507538056
K02316	0.0014592	0.0012920	0.00139980115903102
K02334	0.0005011	0.0005581	0.000363044571051525
K02335	0.0030085	0.0025510	0.00272318929256543
K02337	0.0041110	0.0033972	0.00364215217634804
K02338	0.0012337	0.0010466	0.00110675955315544
K02340	0.0006152	0.0005722	0.000661323680244391
K02341	0.0005452	0.0005397	0.000663417468984513
K02342	0.0008967	0.0009387	0.001098014698375
K02343	0.0015328	0.0013512	0.00136872788863143
K02346	0.0002957	0.0003518	0.000354909618260141
K02355	0.0040072	0.0030048	0.00365001127285915
K02356	0.0007032	0.0006088	0.000607950369658502
K02357	0.0011310	0.0009496	0.000964610799373285
K02358	0.0018897	0.0017249	0.00161724510601112
K02361	0.0002198	0.0002310	0.000521738441566584
K02372	0.0005323	0.0004359	0.000463916461986357
K02377	0.0004335	0.0002739	0.000514616015116104
K02386	0.0002101	0.0001349	0.000112549365784992
K02388	0.0003918	0.0002059	0.000216319966057005
K02390	0.0011261	0.0003428	0.000585763099183364
K02391	0.0002342	0.0002443	0.000144298000492798
K02392	0.0008938	0.0004418	0.00049026259989448

K02046	0,000211	0,000395
K02047	0,000211	0,000395
K02048	0,000211	0,000395
K02049	0,001495	0,001825
K02050	0,001673	0,002236
K02051	0,001089	0,001249
K02052	0,00065	0,000822
K02053	0,00052	0,000477
K02054	0,000406	0,00046
K02055	0,000341	0,000148
K02056	0,001024	0,001315
K02057	0,001592	0,002433
K02058	0,00052	0,000625
K02063	0,000227	0,000493
K02064	0,000227	0,000493
K02065	0,000211	0,000214
K02066	0,000357	0,000559
K02067	0,000292	0,000493
K02071	0,000666	0,001348
K02072	0,000617	0,001348
K02073	0,000536	0,001019
K02078	0,001072	0,001118
K02108	0,00091	0,000707
K02109	0,00104	0,00074
K02110	0,00091	0,000707
K02111	0,00091	0,000707
K02112	0,00091	0,00069
K02113	0,000276	0,000214
K02114	0,00091	0,00069
K02115	0,00091	0,000707
K02117	0,000292	0,000805
K02118	0,000292	0,000805
K02120	0,000211	0,000608
K02121	0,000292	0,000805
K02124	0,000227	0,000658
K02160	0,001251	0,000723
K02188	0,000276	0,000444
K02189	0,000179	0,000395
K02190	0,000211	0,000444
K02203	0,000357	0,000921
K02221	0,000487	0,000592
K02224	0,000747	0,001332
K02227	0,000292	0,000493
K02231	0,000569	0,00097
K02232	0,000406	0,000822
K02233	0,000276	0,000477
K02237	0,00013	0,000148
K02238	0,000715	0,000329
K02241	0,000276	0,000707
K02274	0,000292	0,000132

K02393	0.0005505	0.0003616	0.000315326040868552
K02394	0.0011721	0.0007344	0.00066832709820304
K02396	0.0012875	0.0006707	0.000702324623588378
K02397	0.0004540	0.0001421	0.000226121383505544
K02400	0.0021388	0.0013312	0.00122279005216384
K02401	0.0009816	0.0006349	0.000561097246310615
K02406	0.0012862	0.0005356	0.000678570661061623
K02407	0.0006894	0.0003387	0.000368251164575473
K02409	0.0013241	0.0008238	0.000750086068460525
K02410	0.0008710	0.0005286	0.000492351378645237
K02411	0.0002001	0.0001408	0.000119856060178493
K02412	0.0014593	0.0009284	0.000834479672161276
K02415	0.0002996	0.0001141	0.000155242274325341
K02416	0.0003932	0.0002038	0.000207987298954679
K02417	0.0003236	0.0001730	0.000178451474830839
K02419	0.0006926	0.0004361	0.000394537240701824
K02421	0.0004144	0.0002433	0.000228896613603475
K02427	0.0005628	0.0004141	0.000371629217587377
K02428	0.0003665	0.0004032	0.000633636543825474
K02429	0.0005588	0.0006664	0.00142327086046262
K02433	0.0016517	0.0014790	0.0010688951113867
K02434	0.0016317	0.0012436	0.00105343600943909
K02435	0.0001541	0.0001372	0.000109698254994434
K02437	0.0001672	0.0001383	0.000236552032447865
K02446	0.0010302	0.0006550	0.000576349809069801
K02453	0.0001352	0.0003716	0.000000000000000000
K02454	0.0001328	0.0003570	0.000000000000000000
K02469	0.0034056	0.0028111	0.00292435162304441
K02470	0.0027513	0.0022934	0.00236527470565633
K02471	0.0006378	0.0006231	0.000506686397259969
K02472	0.0001284	0.0001655	0.000298198513733792
K02474	0.0005063	0.0002870	0.000618327072293177
K02477	0.0001813	0.0001808	0.000251564726013014
K02481	0.0015559	0.0001827	0.000848816870864912
K02483	0.0002419	0.0004960	0.000302377426448006
K02488	0.0005259	0.0002567	0.00030320584367963
K02492	0.0003580	0.0003761	0.000357218568008397
K02493	0.0008360	0.0007261	0.000749873906048334
K02495	0.0013907	0.0015944	0.00143287282736186
K02500	0.0002258	0.0002255	0.000259626792651771
K02501	0.0001847	0.0001815	0.000211847769042772
K02503	0.0002767	0.0002582	0.000286398505340915
K02517	0.0002837	0.0005217	0.000507843796775853
K02518	0.0002935	0.0002429	0.000242533136229007
K02519	0.0043823	0.0030981	0.00344549306431214
K02520	0.0005937	0.0004919	0.000531611010440736
K02523	0.0009091	0.0007244	0.000797135671516658
K02527	0.0010303	0.0009056	0.000967665870608551
K02528	0.0010730	0.0008845	0.000908104089611681
K02529	0.0003092	0.0008200	0.00047536551996808

K02275	0,000292	0,000132
K02304	0,000325	0,000773
K02313	0,000975	0,000904
K02314	0,000991	0,000953
K02316	0,000959	0,000904
K02334	0,000796	0,000871
K02335	0,000926	0,000559
K02336	0,00013	0,000378
K02337	0,0013	0,001595
K02338	0,001024	0,000904
K02340	0,001007	0,000888
K02341	0,000747	0,000329
K02342	0,001137	0,000953
K02343	0,001024	0,001036
K02346	0,000422	0,000625
K02355	0,001527	0,002055
K02356	0,000959	0,000904
K02357	0,000959	0,000904
K02358	0,001527	0,001134
K02372	0,000877	0,000888
K02377	0,000341	0,000427
K02379	0,000325	0,000444
K02383	0,000195	0,000493
K02385	0,000211	0,00051
K02387	0,000292	0,000542
K02388	0,000325	0,000592
K02389	0,000292	0,000542
K02390	0,000325	0,000592
K02392	0,000536	0,001167
K02396	0,000292	0,000542
K02397	0,000292	0,000542
K02398	0,00026	0,000542
K02400	0,000422	0,000904
K02401	0,000292	0,000559
K02404	0,00026	0,000542
K02405	0,00026	0,000542
K02406	0,000487	0,000904
K02407	0,000114	0,000181
K02408	0,000292	0,000542
K02409	0,000292	0,000542
K02410	0,000227	0,000345
K02411	0,000244	0,000526
K02412	0,000162	0,000197
K02413	0,00026	0,000542
K02415	0,000179	0,000247
K02416	0,000162	0,000197
K02417	0,000292	0,000559
K02419	0,000292	0,000559
K02420	0,000162	0,000197
K02421	0,000292	0,000559

K02533	0.0005099	0.0003807	0.000357449038440829
K02535	0.0008756	0.0007616	0.000656305744522127
K02536	0.0008370	0.0007752	0.000840338825082333
K02548	0.0001784	0.0001963	0.000292796499676099
K02551	0.0003899	0.0004146	0.00092184174772033
K02556	0.0014774	0.0007290	0.000813484645416755
K02557	0.0012168	0.0006162	0.000743816255048395
K02563	0.0011548	0.0009586	0.00101500594338397
K02564	0.0003326	0.0004045	0.000771209456862329
K02584	0.0012925	0.0003251	0.000994932545844379
K02585	0.0003297	0.0001131	0.000267592106653211
K02586	0.0004452	0.0002475	0.000550903438811276
K02587	0.0003117	0.0001334	0.000281514059431586
K02588	0.0002390	0.0001878	0.00029714646561959
K02591	0.0003826	0.0002179	0.000476672485721512
K02592	0.0002895	0.0001191	0.000259722520678391
K02600	0.0015920	0.0013488	0.00140216643936141
K02601	0.0006535	0.0005329	0.00057088934198063
K02619	0.0001314	0.0001482	0.000196039297555252
K02621	0.0004005	0.0008283	0.00115149069760173
K02622	0.0002713	0.0006472	0.000822740718198651
K02652	0.0002193	0.0003582	0.000252940711056378
K02653	0.0001193	0.0002610	0.000155493949135905
K02654	0.0001206	0.0001667	0.000132202828894024
K02666	0.0001140	0.0002963	0.000249188907267435
K02667	0.0003143	0.0001836	0.000262154789558786
K02669	0.0001555	0.0003028	0.000174237793847005
K02674	0.0001099	0.0006736	0.000550333217023906
K02687	0.0003118	0.0003862	0.000445376951672266
K02823	0.0003404	0.0001950	0.000376709385239919
K02824	0.0004416	0.0004715	0.000681059750167228
K02834	0.0002301	0.0002432	0.000256011457077345
K02835	0.0011603	0.0009699	0.00104573894299531
K02836	0.0012123	0.0010219	0.00108465627523899
K02837	0.0004913	0.0005495	0.000858835034087542
K02838	0.0007363	0.0005996	0.000612249771857764
K02843	0.0003672	0.0003246	0.000362468061139143
K02860	0.0003376	0.0003166	0.000313929158673968
K02863	0.0009017	0.0007322	0.000767212735325536
K02864	0.0004491	0.0003919	0.00043741474588861
K02867	0.0005990	0.0004855	0.000503832776338795
K02871	0.0006088	0.0004907	0.000514029789175786
K02874	0.0004794	0.0003901	0.00040608503935807
K02876	0.0005099	0.0004340	0.000458100520771415
K02878	0.0005470	0.0004463	0.000463532492808564
K02879	0.0005717	0.0004531	0.00050593521762879
K02881	0.0004278	0.0003558	0.000369726261582838
K02884	0.0004246	0.0003530	0.000369469630775752
K02886	0.0010995	0.0008943	0.000934526495257762
K02887	0.0004736	0.0003872	0.000401994081946256

K02422	0,00026	0,000559
K02426	0,000227	0,000148
K02427	0,000179	0,000247
K02428	0,00078	0,000427
K02433	0,002242	0,001743
K02434	0,001868	0,001644
K02435	0,001592	0,000937
K02437	0,00091	0,000674
K02438	0,000406	0,000559
K02456	0,000195	0,000477
K02469	0,001105	0,000921
K02470	0,001056	0,000921
K02474	0,000195	0,000378
K02481	0,00013	0,000247
K02483	0,000601	0,000214
K02488	0,000374	0,000395
K02493	0,000975	0,000904
K02495	0,001121	0,001003
K02499	0,000146	0,000362
K02500	0,00091	0,000707
K02501	0,000894	0,000707
K02503	0,000877	0,000707
K02510	0,000325	0,000444
K02517	0,000162	0,000312
K02518	0,000975	0,000904
K02519	0,000942	0,000888
K02520	0,000877	0,000559
K02523	0,000195	0,000312
K02527	0,000162	0,00023
K02528	0,000829	0,000559
K02529	0,0026	0,003929
K02533	0,000211	0,000247
K02535	0,000162	0,00023
K02536	0,000179	0,000312
K02548	0,00039	0,00023
K02554	0,00013	0,000362
K02556	0,000195	0,000312
K02557	0,00039	0,000756
K02563	0,001446	0,001036
K02564	0,000341	0,000658
K02584	0,000325	0,000773
K02585	0,000211	0,000411
K02586	0,000211	0,000411
K02587	0,000211	0,000411
K02588	0,000487	0,001118
K02589	0,000162	0,000411
K02590	0,000162	0,000411
K02591	0,000357	0,000756
K02592	0,000211	0,000411
K02600	0,000959	0,000904

K02888	0.0003672	0.0003048	0.000320865972088815
K02890	0.0004745	0.0003863	0.000409549819680357
K02892	0.0002555	0.0002301	0.000247752406332827
K02895	0.0004106	0.0003371	0.000347632321151604
K02897	0.0007387	0.0005554	0.000638763249748678
K02899	0.0003415	0.0002827	0.000288429185116979
K02902	0.0002727	0.0002220	0.000232264519629597
K02904	0.0002479	0.0002048	0.000196211940998671
K02906	0.0008212	0.0006756	0.000698418847975559
K02907	0.0001117	0.0001078	0.000119706103842549
K02909	0.0002345	0.0002333	0.000247997432102753
K02911	0.0002520	0.0002032	0.000203287229640013
K02913	0.0002046	0.0001679	0.000183206959206036
K02914	0.0001944	0.0001611	0.000168886480558953
K02916	0.0001684	0.0001474	0.000170650647488885
K02919	0.0001366	0.0001233	0.000125846352879288
K02926	0.0006667	0.0005658	0.000589942106336043
K02931	0.0006944	0.0005652	0.000591276075712638
K02933	0.0006507	0.0005332	0.000574380231010134
K02935	0.0005145	0.0004370	0.000438030978417299
K02939	0.0005597	0.0004487	0.000477986623603325
K02945	0.0024614	0.0016891	0.00203451767174516
K02946	0.0004098	0.0003300	0.000346139596467173
K02948	0.0004981	0.0004067	0.000432386099593555
K02950	0.0004854	0.0003957	0.000421154855062374
K02952	0.0005314	0.0004245	0.000445201836075194
K02954	0.0003395	0.0003039	0.000321399819178792
K02956	0.0003429	0.0002782	0.000290229839605612
K02959	0.0003803	0.0003188	0.000380611669556082
K02961	0.0002927	0.0002422	0.000260069847301485
K02963	0.0002947	0.0002375	0.000258771216344361
K02965	0.0003532	0.0002850	0.000306063995827189
K02967	0.0008845	0.0007457	0.000797737772745616
K02968	0.0003804	0.0003085	0.000308602591066896
K02982	0.0009282	0.0007752	0.000805211625675678
K02986	0.0007742	0.0006331	0.000667071978966206
K02988	0.0006922	0.0005571	0.000577674167397097
K02990	0.0003197	0.0002901	0.000314387499345806
K02992	0.0006168	0.0005015	0.000522422240389239
K02994	0.0004912	0.0004039	0.000423100251912107
K02996	0.0005391	0.0004389	0.000452147096469012
K03040	0.0012697	0.0010207	0.00108721942859011
K03043	0.0051622	0.0041900	0.00440149699611377
K03046	0.0051795	0.0042314	0.00454287646586141
K03060	0.0002407	0.0001871	0.000153556863484926
K03070	0.0033068	0.0027959	0.00305677034453696
K03071	0.0003747	0.0003485	0.000260846206943377
K03072	0.0015501	0.0013114	0.00106028896302987
K03073	0.0001176	0.0001404	0.000138964932706348
K03074	0.0008631	0.0006897	0.000588438062173167

K02601	0,00117	0,001397
K02621	0,000292	0,000608
K02622	0,000292	0,000608
K02652	0,000211	0,00046
K02654	0,001365	0,000526
K02655	0,003428	0,000953
K02669	0,000617	0,000181
K02687	0,000179	0,000214
K02757	0,000227	0,000411
K02759	0,00013	0,000362
K02760	0,000211	0,000378
K02761	0,00013	0,000427
K02793	0,00104	0,000296
K02794	0,000504	0,000181
K02795	0,000504	0,000164
K02796	0,000504	0,000164
K02798	0,000211	0,000362
K02800	0,000211	0,000362
K02806	0,000634	0,000312
K02823	0,000682	0,000641
K02824	0,000114	0,000263
K02825	0,000666	0,000312
K02834	0,000812	0,000542
K02835	0,000959	0,000904
K02836	0,000764	0,000411
K02838	0,000959	0,000904
K02858	0,000552	0,000197
K02860	0,000959	0,000904
K02863	0,000959	0,000904
K02864	0,000829	0,000559
K02867	0,000959	0,000904
K02871	0,000959	0,000904
K02874	0,000959	0,000904
K02876	0,000959	0,000904
K02878	0,000959	0,000904
K02879	0,000959	0,000904
K02881	0,000959	0,000904
K02884	0,000959	0,000904
K02886	0,000959	0,000904
K02887	0,000829	0,000559
K02888	0,000845	0,000542
K02890	0,000959	0,000904
K02892	0,000959	0,000904
K02895	0,000959	0,000904
K02897	0,000959	0,000871
K02899	0,000959	0,000904
K02902	0,000829	0,000559
K02904	0,000829	0,000542
K02906	0,000829	0,000559
K02907	0,000406	0,000707

K03075	0.0001637	0.0001678	0.000180989311049791	K02909	0,000975	0,000904
K03076	0.0018125	0.0014699	0.00152234273264072	K02911	0,000829	0,000559
K03086	0.0019345	0.0016609	0.00152066294842302	K02913	0,000959	0,000904
K03088	0.0014226	0.0017460	0.00295041008906143	K02914	0,000942	0,000822
K03089	0.0007350	0.0005258	0.000484523318881356	K02916	0,000829	0,000559
K03092	0.0012807	0.0009676	0.00118338171877616	K02919	0,000764	0,000542
K03100	0.0013866	0.0012236	0.00193562226218376	K02926	0,000829	0,000559
K03101	0.0005724	0.0005020	0.000506736903716603	K02931	0,000829	0,000559
K03106	0.0017868	0.0014373	0.00150852502742539	K02933	0,000959	0,000904
K03110	0.0011098	0.0009439	0.00102113422515404	K02935	0,000959	0,000904
K03111	0.0007114	0.0006065	0.000601020595745988	K02939	0,000959	0,000904
K03116	0.0002403	0.0001725	0.000208558813498488	K02945	0,000536	0,000756
K03118	0.0009348	0.0006914	0.000812896763272281	K02946	0,000959	0,000904
K03147	0.0004064	0.0006395	0.000602318635073642	K02948	0,000959	0,000904
K03149	0.0001834	0.0002470	0.000159516930320457	K02950	0,000829	0,000559
K03150	0.0004629	0.0001838	0.000221872327471688	K02952	0,000959	0,000904
K03152	0.0001107	0.0001838	0.000262190966679566	K02954	0,000975	0,000904
K03168	0.0030295	0.0023371	0.00261391462366545	K02956	0,000764	0,000411
K03169	0.0006837	0.0009854	0.00131584818617631	K02959	0,000959	0,000904
K03177	0.0010277	0.0008470	0.000864571720905733	K02961	0,000959	0,000904
K03179	0.0007298	0.0005606	0.000551016576566972	K02963	0,000829	0,000559
K03182	0.0004098	0.0002583	0.0003273142864003	K02965	0,000959	0,000904
K03183	0.0009228	0.0007156	0.000802490447552052	K02967	0,000829	0,000559
K03185	0.0003794	0.0004107	0.000229403778242198	K02968	0,000959	0,000904
K03186	0.0001297	0.0001144	0.000123690381532469	K02970	0,000926	0,000904
K03195	0.0004774	0.0004296	0.000285091880300791	K02982	0,000829	0,000559
K03196	0.0008050	0.0006368	0.000478883816084639	K02986	0,000959	0,000904
K03198	0.0001894	0.0001418	0.000112631688619094	K02988	0,000959	0,000904
K03199	0.0016601	0.0013390	0.000990360954093967	K02990	0,000829	0,000559
K03201	0.0008922	0.0006300	0.000527587673977718	K02992	0,000829	0,000559
K03203	0.0002342	0.0001791	0.000138573859107006	K02994	0,000959	0,000904
K03204	0.0006616	0.0005265	0.000393593557252968	K02996	0,000829	0,000559
K03205	0.0013273	0.0010343	0.000791183068371529	K03040	0,000959	0,000904
K03210	0.0003089	0.0002667	0.000283424051560366	K03043	0,000959	0,000904
K03215	0.0003415	0.0004128	0.000596419713355889	K03046	0,001007	0,000904
K03217	0.0018287	0.0014660	0.00158417376962017	K03060	0,000309	0,000296
K03218	0.0008730	0.0007380	0.000789257163306703	K03070	0,000975	0,000904
K03230	0.0002032	0.0001348	0.00010149752980068	K03072	0,000942	0,000805
K03269	0.0001079	0.0002431	0.000317889980838297	K03073	0,000959	0,000904
K03270	0.0001766	0.0002174	0.000244553323495223	K03074	0,000942	0,000805
K03271	0.0001977	0.0001625	0.000146557577796548	K03075	0,000959	0,000904
K03272	0.0002070	0.0003098	0.000218958135095589	K03076	0,000959	0,000904
K03273	0.0001281	0.0001643	0.000113085332018677	K03086	0,001316	0,001759
K03274	0.0002578	0.0002690	0.000218987131433198	K03088	0,00208	0,002449
K03281	0.0006503	0.0004645	0.000861296579364493	K03092	0,000325	0,000625
K03284	0.0007902	0.0007150	0.000766443017530957	K03100	0,001511	0,002055
K03286	0.0003737	0.0004169	0.000475336288169365	K03101	0,000991	0,000871
K03294	0.0014169	0.0011307	0.00123162542285397	K03106	0,000341	0,000427
K03296	0.0040496	0.0036085	0.00457621400646637	K03110	0,000341	0,000427
K03301	0.0013616	0.0009128	0.000799610328847435	K03111	0,001202	0,001019

K03303	0.0008698	0.0003737	0.000826591898334927
K03305	0.0002838	0.0004645	0.00068249286761477
K03306	0.0003346	0.0002636	0.0004110215833748
K03307	0.0005716	0.0003014	0.000483419076338633
K03308	0.0004432	0.0007268	0.000827390850416843
K03310	0.0010071	0.0013469	0.000949590194664132
K03313	0.0011437	0.0008030	0.000989676391269509
K03315	0.0001134	0.0004968	0.000170637372026759
K03319	0.0007039	0.0006013	0.000537185221349313
K03320	0.0009225	0.0008090	0.00101557260847427
K03321	0.0006308	0.0004364	0.000812503590782565
K03322	0.0001375	0.0001981	0.00020079493576226
K03324	0.0002005	0.0003636	0.000354662668461716
K03325	0.0002088	0.0001853	0.000225973019086349
K03327	0.0006073	0.0005880	0.000824519329020757
K03328	0.0001026	0.0002395	0.000220191382905561
K03386	0.0007972	0.0006790	0.000663310217439644
K03394	0.0002132	0.0001808	0.000258294815030654
K03406	0.0082661	0.0018729	0.00420351310907999
K03407	0.0013819	0.0004307	0.000725226118418503
K03412	0.0005792	0.0001920	0.00032020873141577
K03413	0.0005740	0.0001238	0.000321097593786783
K03423	0.0005458	0.0004549	0.000355636010493761
K03424	0.0011700	0.0011310	0.00117316318523618
K03427	0.0007630	0.0009142	0.00121727244952887
K03431	0.0014304	0.0011643	0.000927656635190346
K03437	0.0001168	0.0002938	0.000344058273447895
K03438	0.0011412	0.0009511	0.000986444956805112
K03439	0.0004478	0.0005228	0.000540031585465938
K03442	0.0001409	0.0002749	0.000150493996876638
K03446	0.0001731	0.0004464	0.00029509925546337
K03453	0.0002637	0.0002554	0.000532325691741766
K03455	0.0017727	0.0013979	0.00153674900650868
K03465	0.0009659	0.0005390	0.000551366808823718
K03466	0.0025031	0.0022946	0.00251850868628028
K03469	0.0005886	0.0004554	0.000543545008614744
K03470	0.0007762	0.0006402	0.000668781770663906
K03473	0.0001382	0.0001626	0.00032721860153154
K03474	0.0002195	0.0002560	0.000377239486091472
K03495	0.0021727	0.0019573	0.00190404105799989
K03496	0.0019622	0.0013659	0.00143494056661266
K03497	0.0009820	0.0009087	0.000878788821280358
K03498	0.0006504	0.0005368	0.000677456129675273
K03499	0.0004801	0.0004296	0.000544920507747016
K03500	0.0002733	0.0005931	0.000375928293238586
K03501	0.0003600	0.0003787	0.000421554578945101
K03502	0.0002806	0.0001506	0.000261256662530418
K03517	0.0004104	0.0004269	0.000566871742146081
K03521	0.0002833	0.0005890	0.000627680748298965
K03522	0.0003460	0.0007579	0.000787077963763011

K03116	0,000422	0,000214
K03118	0,000341	0,000181
K03147	0,000617	0,000214
K03149	0,000601	0,000197
K03150	0,000682	0,000526
K03151	0,000211	0,000559
K03152	0,000244	0,000608
K03154	0,000747	0,000559
K03168	0,00091	0,000592
K03169	0,00013	0,000279
K03177	0,000959	0,000904
K03183	0,000601	0,000411
K03210	0,00078	0,000542
K03215	0,00013	0,000181
K03217	0,00104	0,000904
K03218	0,000959	0,000904
K03270	0,000552	0,000279
K03271	0,000325	0,000575
K03272	0,000162	0,000148
K03273	0,000211	0,000148
K03282	0,000179	0,000181
K03284	0,000926	0,000838
K03287	0,000292	0,000427
K03292	0,000341	0,000526
K03293	0,00065	0,000214
K03294	0,000357	0,000575
K03296	0,000861	0,001479
K03305	0,001024	0,000395
K03307	0,000812	0,000855
K03308	0,000634	0,000592
K03310	0,000357	0,000805
K03312	0,000146	0,000214
K03315	0,000146	0,00046
K03321	0,00026	0,000493
K03324	0,00013	0,000263
K03327	0,000211	0,000214
K03328	0,000422	0,000444
K03336	0,000179	0,000411
K03337	0,000179	0,000411
K03386	0,000227	0,000395
K03394	0,000276	0,00023
K03406	0,001657	0,00286
K03407	0,000536	0,001118
K03408	0,00078	0,001299
K03409	0,000211	0,000559
K03412	0,000601	0,001134
K03413	0,000439	0,000904
K03424	0,00143	0,002006
K03426	0,000341	0,000608
K03427	0,001332	0,001759

K03524	0.0006830	0.0005396	0.000540394040502799
K03525	0.0006378	0.0004881	0.000438713264906554
K03526	0.0015867	0.0013158	0.00141422118297899
K03527	0.0011441	0.0009479	0.00097525718966791
K03530	0.0007775	0.0005766	0.000647456982557351
K03531	0.0015133	0.0012211	0.00126715900830203
K03536	0.0001635	0.0001693	0.000201885167568855
K03543	0.0001431	0.0003404	0.000262981801472812
K03544	0.0016388	0.0013408	0.00136871622189474
K03545	0.0011154	0.0009943	0.00104156069605633
K03546	0.0002682	0.0005581	0.000547069518498999
K03549	0.0002750	0.0004177	0.000349334903221723
K03550	0.0006592	0.0005455	0.000589926400743332
K03551	0.0013162	0.0010842	0.00113959966891376
K03553	0.0013753	0.0011113	0.00116557108024617
K03555	0.0030096	0.0024773	0.00272917160980615
K03558	0.0002464	0.0002326	0.000197337872498431
K03559	0.0001069	0.0002695	0.000197830915158313
K03560	0.0004538	0.0002871	0.000255665816611914
K03561	0.0003691	0.0005515	0.000631559196633435
K03562	0.0005628	0.0003547	0.000314087458168602
K03563	0.0001906	0.0001052	0.000106875996262227
K03564	0.0005423	0.0004719	0.000556723502763352
K03568	0.0001413	0.0005027	0.000465903185414557
K03569	0.0016343	0.0012218	0.00120896077486671
K03570	0.0004395	0.0003677	0.00040501044603021
K03572	0.0018567	0.0015382	0.00165826051321932
K03574	0.0002431	0.0002523	0.000248153770403459
K03575	0.0004010	0.0003475	0.000560951951904027
K03579	0.0002427	0.0001282	0.000114979737489392
K03581	0.0007593	0.0002784	0.000537182302673457
K03582	0.0002305	0.0006134	0.000524585127944237
K03584	0.0002782	0.0003002	0.000345883591588757
K03585	0.0004503	0.0004559	0.000434291623597348
K03587	0.0018793	0.0016142	0.00172736496388028
K03588	0.0010983	0.0009016	0.00101396391296525
K03589	0.0002263	0.0002781	0.000315719921213934
K03590	0.0010417	0.0008101	0.000885374046104835
K03592	0.0001369	0.0004205	0.000440035601594865
K03593	0.0009898	0.0007979	0.000958102512281722
K03594	0.0001144	0.0001838	0.000136016220711978
K03595	0.0010581	0.0008860	0.000926259891219256
K03596	0.0024643	0.0020073	0.00209590573311765
K03601	0.0012071	0.0010123	0.00106388709421598
K03602	0.0001510	0.0001508	0.000138953093616033
K03606	0.0002880	0.0002802	0.000429935588007826
K03612	0.0001715	0.0001139	0.000171113612506374
K03613	0.0002131	0.0001537	0.000211816663726014
K03614	0.0002861	0.0002590	0.00031628881216549
K03615	0.0003135	0.0004139	0.00041064216480391

K03431	0,000747	0,000345
K03437	0,000894	0,000822
K03438	0,000829	0,000559
K03439	0,000309	0,000395
K03442	0,000292	0,000197
K03455	0,000682	0,000296
K03465	0,000764	0,000773
K03466	0,000877	0,000575
K03469	0,000959	0,000855
K03470	0,000894	0,000756
K03474	0,000634	0,000263
K03488	0,000227	0,000395
K03495	0,000877	0,000888
K03496	0,0013	0,00074
K03497	0,001251	0,001397
K03498	0,000455	0,000855
K03499	0,000715	0,001134
K03500	0,000309	0,000197
K03501	0,000829	0,000559
K03517	0,000699	0,000247
K03518	0,000292	0,000608
K03521	0,000845	0,000674
K03522	0,000845	0,000674
K03523	0,000292	0,000296
K03524	0,000959	0,000904
K03525	0,000715	0,00046
K03526	0,000829	0,000542
K03527	0,000829	0,000559
K03529	0,000926	0,000805
K03530	0,001332	0,00069
K03531	0,000959	0,000904
K03536	0,000959	0,000904
K03544	0,000959	0,000904
K03545	0,000959	0,000904
K03546	0,000341	0,000559
K03547	0,000211	0,000197
K03550	0,000959	0,000904
K03551	0,000959	0,000904
K03553	0,000829	0,000559
K03555	0,000877	0,000888
K03558	0,000309	0,000625
K03559	0,000439	0,00046
K03561	0,000455	0,000756
K03563	0,000227	0,000526
K03564	0,000292	0,000181
K03565	0,000845	0,000838
K03568	0,000211	0,000181
K03569	0,00091	0,000986
K03570	0,000877	0,000855
K03571	0,000764	0,000756

K03617	0.0001999	0.0001480	0.0002049995740695
K03621	0.0009678	0.0007787	0.000640638665095844
K03624	0.0006125	0.0004513	0.000518877829464173
K03625	0.0004044	0.0003471	0.000471133814733874
K03628	0.0017366	0.0014280	0.00156155079377029
K03629	0.0005833	0.0005622	0.000618757933913905
K03630	0.0002502	0.0003044	0.000367804509178853
K03631	0.0010419	0.0009903	0.00110924003573007
K03634	0.0001218	0.0001266	0.000122054230899525
K03639	0.0002612	0.0002130	0.00014113884567275
K03640	0.0004847	0.0003466	0.000322610944215396
K03641	0.0013015	0.0008921	0.000871211662238789
K03642	0.0003578	0.0003165	0.000288478918227654
K03644	0.0011290	0.0009097	0.000979301450832334
K03648	0.0001516	0.0002967	0.000330945248719456
K03650	0.0013806	0.0011977	0.00125810719084073
K03652	0.0001722	0.0001427	0.000153898928585693
K03654	0.0008773	0.0012213	0.00176127948343952
K03655	0.0022834	0.0024551	0.00271544384036125
K03657	0.0031680	0.0026580	0.00262930018867965
K03664	0.0006604	0.0005348	0.000552014835569867
K03665	0.0005284	0.0006002	0.000742770987369615
K03667	0.0014795	0.0009746	0.000840400086406489
K03671	0.0005582	0.0004587	0.000576833423718667
K03684	0.0005300	0.0004036	0.000311830389825299
K03685	0.0008398	0.0007003	0.000725181883320041
K03686	0.0017434	0.0013157	0.00140118318433126
K03687	0.0005721	0.0004838	0.000499796680595644
K03688	0.0009697	0.0010174	0.000713053544888482
K03694	0.0005560	0.0004629	0.000468479546530213
K03695	0.0032818	0.0026908	0.00282074996315086
K03696	0.0004582	0.0004185	0.000901035528133269
K03699	0.0013221	0.0010574	0.00117050339271714
K03701	0.0044438	0.0040604	0.00440778757091298
K03702	0.0026418	0.0021758	0.00228215302677759
K03703	0.0020008	0.0016834	0.00179804979991028
K03711	0.0006693	0.0004385	0.000531801836580704
K03718	0.0001195	0.0001018	0.000234190124792438
K03723	0.0024600	0.0022877	0.00260952198561476
K03733	0.0010801	0.0008049	0.000883855424600704
K03734	0.0002935	0.0004432	0.000526113207978372
K03735	0.0001860	0.0002527	0.000235105463357084
K03736	0.0001017	0.0001333	0.000127403008150614
K03737	0.0017822	0.0014229	0.00192419745182741
K03742	0.0005033	0.0005167	0.000599802231201181
K03744	0.0001964	0.0002332	0.000313840353823348
K03750	0.0003426	0.0002801	0.000168385094337087
K03762	0.0007323	0.0007978	0.000435064049882247
K03768	0.0003569	0.0002648	0.000422438576321681
K03770	0.0007712	0.0007109	0.000842325874018197

K03572	0,000877	0,000888
K03574	0,000861	0,001414
K03575	0,000471	0,000723
K03584	0,000341	0,000411
K03585	0,000146	0,000214
K03587	0,000959	0,000838
K03588	0,000959	0,000904
K03589	0,000942	0,000822
K03590	0,000812	0,000871
K03592	0,000146	0,000181
K03593	0,000276	0,000181
K03595	0,000471	0,000773
K03596	0,000959	0,000904
K03601	0,000959	0,000904
K03602	0,000959	0,000904
K03612	0,000179	0,000493
K03613	0,000244	0,000625
K03614	0,000406	0,001036
K03615	0,000406	0,001036
K03617	0,000244	0,000625
K03621	0,00065	0,000362
K03624	0,000715	0,000411
K03625	0,000959	0,000904
K03628	0,00078	0,000542
K03629	0,000422	0,000756
K03630	0,000894	0,000904
K03631	0,000959	0,000904
K03637	0,000374	0,000493
K03639	0,00039	0,000526
K03640	0,000617	0,000296
K03642	0,000341	0,000641
K03644	0,000764	0,000312
K03648	0,000146	0,000148
K03650	0,000877	0,000888
K03653	0,000764	0,000838
K03654	0,000796	0,001216
K03655	0,00117	0,001512
K03657	0,001316	0,001151
K03664	0,000959	0,000904
K03665	0,00026	0,000197
K03667	0,000292	0,000658
K03671	0,001154	0,001101
K03684	0,000422	0,000493
K03685	0,001007	0,000904
K03686	0,001251	0,001266
K03687	0,000959	0,000904
K03694	0,000309	0,000608
K03695	0,001007	0,000904
K03696	0,00052	0,000674
K03699	0,000227	0,00046

K03771	0.0007124	0.0007324	0.00105666257772327
K03773	0.0001746	0.0001848	0.000415515537455552
K03775	0.0001126	0.0002329	0.000267148596280067
K03781	0.0003764	0.0003530	0.000312611582932221
K03782	0.0001665	0.0002612	0.000000000000000000
K03783	0.0002780	0.0001455	0.000362567570410874
K03786	0.0001602	0.0001831	0.000207502721084472
K03787	0.0003175	0.0002951	0.000441777486761136
K03789	0.0001114	0.0001347	0.000127281972221561
K03797	0.0019695	0.0018299	0.00249143999224983
K03798	0.0025165	0.0020777	0.00212243207031513
K03799	0.0003473	0.0003486	0.000393875389409452
K03801	0.0006041	0.0005014	0.000565061286793802
K03814	0.0001377	0.0002249	0.000302125996884095
K03820	0.0012027	0.0008776	0.000816487466145871
K03821	0.0009868	0.0009018	0.000596336746367167
K03832	0.0006310	0.0007407	0.000767804692299194
K03833	0.0004249	0.0001179	0.000206833890474611
K03834	0.0005385	0.0003439	0.000310366271324772
K03841	0.0002662	0.0002081	0.000213650312692614
K03885	0.0001417	0.0002362	0.00018790375816584
K03892	0.0002926	0.0001487	0.000176684947242956
K03924	0.0003418	0.0004745	0.000738363632678843
K03925	0.0003455	0.0002604	0.000253717636823489
K03926	0.0002145	0.0001302	0.000119581065762473
K03929	0.0002014	0.0002477	0.000390964667802116
K03977	0.0017606	0.0014643	0.00152881645145478
K03978	0.0006400	0.0005669	0.000578414219240079
K03979	0.0011571	0.0010228	0.00108764962088777
K03980	0.0014519	0.0011215	0.000952376511846624
K04042	0.0014051	0.0011526	0.000933418772299034
K04043	0.0027247	0.0022150	0.00227596408881131
K04044	0.0008901	0.0009406	0.00071672780442946
K04066	0.0020867	0.0017660	0.00193977599842635
K04069	0.0006519	0.0003287	0.000451360944465289
K04072	0.0002186	0.0003849	0.000352970530191284
K04075	0.0009862	0.0009068	0.000969125141717227
K04077	0.0023954	0.0019053	0.00197896857811534
K04078	0.0004203	0.0003315	0.000353818823911523
K04079	0.0019736	0.0016331	0.0017403622034677
K04087	0.0008624	0.0006003	0.000490863248478888
K04088	0.0011238	0.0007858	0.00063915240624855
K04094	0.0005703	0.0002999	0.000297778293202173
K04096	0.0006752	0.0006426	0.000708893193607591
K04485	0.0016202	0.0013798	0.00145132265347881
K04486	0.0001445	0.0001088	0.000135348803707849
K04487	0.0023891	0.0018679	0.00149520803374391
K04488	0.0003368	0.0003463	0.000233221045162137
K04516	0.0001658	0.0001492	0.000376665697501144
K04517	0.0001286	0.0001576	0.000116005579797314

K03701	0,001007	0,000986
K03702	0,000959	0,000904
K03703	0,000959	0,000904
K03704	0,001056	0,000641
K03705	0,000715	0,000296
K03709	0,000325	0,000427
K03710	0,000406	0,000148
K03711	0,000634	0,000608
K03712	0,000682	0,000247
K03716	0,000487	0,000132
K03719	0,000309	0,000132
K03720	0,00013	0,000345
K03723	0,000959	0,000904
K03733	0,000747	0,000378
K03734	0,00052	0,000723
K03737	0,000325	0,000674
K03741	0,000179	0,000181
K03742	0,000634	0,000296
K03743	0,000374	0,000575
K03744	0,000747	0,00046
K03750	0,000455	0,000526
K03752	0,000292	0,000427
K03768	0,000292	0,000164
K03769	0,000244	0,000493
K03770	0,000601	0,00023
K03771	0,000617	0,000263
K03773	0,000162	0,000444
K03775	0,000244	0,000608
K03778	0,000146	0,00023
K03782	0,000195	0,000148
K03783	0,000634	0,000296
K03784	0,000244	0,000526
K03786	0,00078	0,000312
K03787	0,00091	0,000822
K03789	0,000715	0,000247
K03797	0,000325	0,000411
K03798	0,001738	0,00143
K03799	0,000569	0,000181
K03800	0,000812	0,000674
K03801	0,000227	0,000164
K03820	0,001024	0,000904
K03823	0,000455	0,000608
K03832	0,000455	0,000953
K03833	0,000162	0,000427
K03837	0,000504	0,000132
K03856	0,000731	0,000526
K03885	0,000292	0,000132
K03892	0,000471	0,000493
K03924	0,001137	0,000855
K03925	0,000422	0,00074

K04518	0.0001285:	0.0001218‹	0.000301623314654895
K04564	0.0007916ℓ	0.0006615(	0.000742664111048318
K04566	0.0012648!	0.0008581!	0.000748544897824126
K04567	0.0006472:	0.0007188:	0.000936826901715656
K04656	0.0005793:	0.0001009	0.000279200967299111
K04720	0.0001831ℓ	0.0002387(	0.000330412113770849
K04744	0.0005613(	0.0006483(	0.000512875687032322
K04751	0.0001526(	0.0001435	0.000163904293427709
K04754	0.0006858:	0.0005660:	0.000454013629685965
K04755	0.0002342‹	0.0002268(	0.00017017702502436
K04756	0.0001873!	0.0001434(	0.000110858559927615
K04759	0.0023663!	0.0014180	0.00194326124227602
K04761	0.0001487:	0.0003550:	0.000370502622651821
K04763	0.0012082:	0.0009352‹	0.00110347725439931
K04764	0.0001666‹	0.0001347!	0.000115451860220932
K04773	0.0004614	0.0006020‹	0.000732295730826314
K05349	0.0017208	0.0019108ℓ	0.004055400214919
K05366	0.0033351:	0.0028268(	0.00294637409229504
K05367	0.0003901(	0.0005029!	0.00058621295902605
K05515	0.0017841(	0.0014721(	0.00148963758071971
K05524	0.0002840:	0.0002406(	0.000175836866717669
K05540	0.0002119‹	0.0002869(	0.00034598231146066
K05592	0.0010129:	0.0007434:	0.00117925586490265
K05595	0.0002454!	0.0002629:	0.000375470656654543
K05606	0.0001142(	0.0001088!	0.000182810276976141
K05776	0.0002281(	0.0001055:	0.000180446000934092
K05788	0.0001818:	0.0001288!	0.000123174859767688
K05794	0.0008022(	0.0005941(	0.000621383495200762
K05799	0.0001225ℓ	0.0001642(	0.000126595699809716
K05807	0.0007380:	0.0005900!	0.000691877448435319
K05808	0.0002056(	0.0001597(	0.000181456026588162
K05810	0.0008268:	0.0006525:	0.000614692124231045
K05832	0.0005882:	0.0004506!	0.000337057382235894
K05833	0.0005252:	0.0003982(	0.000301502882663608
K05837	0.0012729:	0.0009932ℓ	0.00106605686494187
K05844	0.0004619ℓ	0.0002594(	0.000257446236355136
K05934	0.0002174!	0.0001832:	0.000243897287108524
K05936	0.0002504:	0.0002135‹	0.000302811402603977
K05970	0.0004188(	0.0004130‹	0.000991102458364905
K05989	0.0002127:	0.0002063ℓ	0.000503363201726367
K06001	0.0003104!	0.0002296‹	0.00056015421279616
K06013	0.0001573‹	0.0001633:	0.000191837192050695
K06020	0.0002713(	0.0003938	0.00043940064465033
K06041	0.0010750	0.0009144:	0.000893934240090672
K06042	0.0001837:	0.0001646ℓ	0.000206218162365534
K06076	0.0003232(	0.0002496ℓ	0.000295180715559124
K06131	0.0005445(	0.0005399:	0.000656043495660907
K06134	0.0003747ℓ	0.0003190!	0.000225117703101615
K06137	0.0003279:	0.0002564ℓ	0.000195110656770159
K06142	0.0003463ℓ	0.0003051!	0.00058743494711806

K03927	0,000601	0,001085
K03969	0,000292	0,000362
K03976	0,000244	0,000592
K03977	0,000829	0,000542
K03978	0,000699	0,000542
K03979	0,000764	0,000411
K03980	0,000812	0,00046
K04042	0,001478	0,000625
K04043	0,001202	0,001315
K04061	0,000244	0,000542
K04066	0,000959	0,000904
K04068	0,00026	0,00046
K04069	0,001089	0,001364
K04070	0,000211	0,000575
K04072	0,001836	0,001216
K04073	0,000146	0,000362
K04075	0,000959	0,000904
K04077	0,001024	0,000921
K04078	0,000975	0,000921
K04079	0,000439	0,00069
K04083	0,000179	0,000214
K04087	0,000309	0,000608
K04088	0,000162	0,000247
K04095	0,000146	0,000362
K04096	0,000959	0,000871
K04477	0,000227	0,000395
K04485	0,000959	0,000904
K04486	0,000244	0,000526
K04487	0,00156	0,000888
K04488	0,000845	0,000789
K04517	0,000764	0,000542
K04562	0,000325	0,00069
K04564	0,00026	0,000197
K04566	0,000244	0,000559
K04567	0,000715	0,000345
K04568	0,000227	0,000493
K04720	0,000179	0,000427
K04744	0,001056	0,000395
K04750	0,000276	0,000164
K04751	0,000357	0,000477
K04758	0,000699	0,000559
K04759	0,000829	0,000871
K04763	0,001121	0,001069
K04773	0,000227	0,000263
K04835	0,000162	0,000362
K05349	0,00052	0,000526
K05350	0,000471	0,00074
K05351	0,00013	0,000345
K05366	0,001397	0,000855
K05367	0,000276	0,000625

K06147	0.0057187	0.0057576	0.00776003192008518
K06148	0.0010022	0.0009481	0.000605690552182125
K06153	0.0003413	0.0004122	0.000488857767156011
K06158	0.0009291	0.0010755	0.00150503288304763
K06167	0.0006478	0.0004736	0.000455197611359391
K06168	0.0019194	0.0015274	0.00163398741561645
K06173	0.0009237	0.0007497	0.000801940701336191
K06177	0.0001022	0.0001722	0.000177351983653474
K06178	0.0007997	0.0007951	0.000857109762699903
K06179	0.0009780	0.0008149	0.000644966590371736
K06180	0.0015618	0.0012190	0.00164408186723108
K06187	0.0007676	0.0006323	0.000663123074741426
K06189	0.0002284	0.0002112	0.000200463353118503
K06190	0.0001873	0.0002259	0.000159627868791996
K06192	0.0001821	0.0002226	0.000206498180597656
K06194	0.0001053	0.0002943	0.000183430657516522
K06200	0.0001376	0.0003547	0.000252998413198767
K06204	0.0005036	0.0003354	0.00032380564875806
K06207	0.0024699	0.0020128	0.00210211843377495
K06213	0.0003115	0.0003499	0.000311651213378575
K06217	0.0003278	0.0003435	0.000512328048758146
K06223	0.0016571	0.0012501	0.00101707408053616
K06287	0.0007107	0.0006742	0.00065206635823372
K06861	0.0008880	0.0007574	0.000785107028927788
K06871	0.0008958	0.0008543	0.00194717534894483
K06872	0.0001373	0.0001546	0.000179253827475817
K06879	0.0006473	0.0005492	0.000453197229171217
K06881	0.0005758	0.0002215	0.000554027745345589
K06901	0.0002935	0.0008779	0.000652912421002388
K06919	0.0001733	0.0002772	0.000139133846477222
K06920	0.0008215	0.0006780	0.000731319542852079
K06925	0.0004730	0.0004200	0.00041118294519915
K06941	0.0006766	0.0007137	0.000853832945633379
K06949	0.0003361	0.0004265	0.000528792603444578
K06950	0.0007615	0.0005120	0.00100785675115924
K06958	0.0002263	0.0002755	0.000209152088445637
K06959	0.0006798	0.0010115	0.00116790403467046
K06969	0.0003445	0.0005087	0.000625311642489923
K06999	0.0004275	0.0003806	0.000258679637605541
K07001	0.0006293	0.0010073	0.00133311715536439
K07010	0.0005652	0.0005683	0.000382809597359098
K07025	0.0003581	0.0004706	0.000692727130118307
K07042	0.0003638	0.0003435	0.000275863812590742
K07056	0.0009941	0.0009547	0.00110671769363485
K07058	0.0007289	0.0008250	0.000830568546651538
K07082	0.0009252	0.0007711	0.000823151531422981
K07085	0.0001151	0.0003353	0.000142881665400627
K07106	0.0001278	0.0001469	0.000301313167617756
K07107	0.0002221	0.0002587	0.000328637631984502
K07114	0.0004554	0.0005918	0.000966462141409621

K05515	0,000877	0,000888
K05520	0,000747	0,000329
K05521	0,000244	0,000395
K05540	0,000569	0,000214
K05568	0,000146	0,000197
K05592	0,000731	0,000329
K05595	0,000682	0,000395
K05685	0,000504	0,000132
K05777	0,000146	0,000362
K05788	0,000244	0,000427
K05796	0,000276	0,000707
K05799	0,000504	0,000937
K05807	0,00013	0,000164
K05808	0,000276	0,000559
K05810	0,000325	0,000592
K05813	0,000569	0,001085
K05814	0,000292	0,000378
K05815	0,000309	0,000378
K05820	0,000146	0,000378
K05832	0,000227	0,000526
K05833	0,000227	0,000526
K05837	0,000747	0,000542
K05846	0,000276	0,000148
K05896	0,000926	0,000805
K05903	0,00052	0,000164
K05919	9,75E-05	0,000247
K05934	0,000179	0,000395
K05936	0,00026	0,000411
K05939	0,000487	0,000132
K05989	0,000341	0,000148
K05993	0,000276	0,000395
K06001	0,000227	0,000427
K06013	0,000536	0,000197
K06015	0,000195	0,000493
K06016	0,000309	0,000444
K06023	0,000942	0,001069
K06024	0,000926	0,000805
K06041	0,00013	0,00023
K06042	0,000309	0,000411
K06076	0,000552	0,000197
K06131	0,000926	0,000855
K06142	0,000244	0,000493
K06143	0,000114	0,000197
K06147	0,003786	0,005343
K06148	0,000439	0,000345
K06153	0,000487	0,000756
K06158	0,00039	0,000723
K06167	0,000276	0,000641
K06168	0,000877	0,000888
K06173	0,000487	0,000789

K07146	0.0003780:	0.0003730(	0.00031251058718251
K07154	0.0004597(	0.0005919:	0.000900598177869056
K07175	0.0004484!	0.0004560!	0.000615422368087043
K07192	0.0001471:	0.0001410!	0.000344634625328358
K07214	0.0002621!	0.0002860:	0.000621964236105415
K07238	0.0003038!	0.0002079	0.000340128422667417
K07240	0.0002618!	0.0004073.	0.00032370610170256
K07258	0.0010022!	0.0009639!	0.00068304831630137
K07259	0.0005127:	0.0006319!	0.000676462576777049
K07263	0.0011646!	0.0007270:	0.00172884273767326
K07277	0.0022468!	0.0018428:	0.00214409342814207
K07278	0.0001219!	0.0001552(	0.000146760130898013
K07282	0.0001249:	0.0001473:	0.000307050821481073
K07301	0.0003477.	0.0002006!	0.000335526936222799
K07319	0.0001567(	0.0001570:	0.00021495473999846
K07323	0.0001395(	0.0001516:	0.000123699766177051
K07386	0.0003737:	0.0004519	0.000887031148769569
K07390	0.0002810!	0.0002873!	0.000213900968156193
K07391	0.0012736:	0.0011022:	0.00128777930334745
K07402	0.0001692!	0.0001844!	0.000000000000000000
K07405	0.0002941(	0.0001572(	0.000451433759794661
K07407	0.0001748:	0.0001967.	0.000410443852105224
K07444	0.0002982(	0.0004603	0.000570238407794566
K07447	0.0003985:	0.0003526:	0.000374835216720554
K07456	0.0007465	0.0005703	0.00102400111713339
K07460	0.0002732(	0.0002393!	0.000273868332483073
K07462	0.0019364	0.0017956(	0.00173457531478222
K07478	0.0005366	0.0006111:	0.000791961472712982
K07486	0.0001746!	0.0007842(	0.000386868346230379
K07487	0.0002359.	0.0007474:	0.00011166527894336
K07488	0.0002101(	0.0007489!	0.00119011765150426
K07497	0.0014264(	0.0019815!	0.000982611409774878
K07501	0.0002754!	0.0002941!	0.000306267734280149
K07552	0.0007190!	0.0007380!	0.000539936450369399
K07560	0.0001845:	0.0001882:	0.000268289164690646
K07566	0.0007313(	0.0007675!	0.000844751559838172
K07567	0.0001954:	0.0001957.	0.000246300262630381
K07568	0.0013279(	0.0011504!	0.00148810974453325
K07576	0.0004619!	0.0001842!	0.000365622491506039
K07588	0.0001652!	0.0002271	0.000387514360428203
K07636	0.0007150(	0.0005380!	0.000907732267147735
K07638	0.0006369!	0.0006057	0.000376301526258143
K07646	0.0003371(	0.0004125(	0.000448871781009364
K07657	0.0006319!	0.0004810(	0.000478868907673262
K07659	0.0002991(	0.0002951	0.000202594568432586
K07667	0.0001133.	0.0001636!	0.000180476282308472
K07678	0.0002330	0.0001069	0.000116526252313554
K07712	0.0005311(	0.0003620:	0.000307016463770585
K07715	0.0004279(	0.0001259!	0.000220140037440986
K07735	0.0001902.	0.0002554	0.000245362928927318

K06177	0,000211	0,000197
K06178	0,000471	0,000756
K06179	0,000861	0,00097
K06180	0,001202	0,001512
K06187	0,000829	0,000559
K06196	0,000422	0,000575
K06199	0,000504	0,00051
K06200	0,000227	0,000427
K06201	0,000179	0,000214
K06204	0,000634	0,000279
K06206	0,000244	0,00046
K06207	0,000276	0,000279
K06208	0,000244	0,000493
K06213	0,00039	0,000805
K06217	0,000471	0,000773
K06218	0,000211	0,000148
K06223	0,000325	0,00051
K06287	0,000829	0,000427
K06381	0,000552	0,000214
K06442	0,000666	0,000247
K06518	0,00026	0,000641
K06603	0,000227	0,000526
K06606	0,00013	0,000345
K06861	0,000845	0,000855
K06864	0,000699	0,000559
K06867	0,000357	0,000674
K06871	0,000341	0,000608
K06877	0,000211	0,000362
K06878	0,000114	0,000197
K06881	0,001154	0,001627
K06889	0,000536	0,00051
K06891	0,000471	0,000542
K06896	0,000325	0,000312
K06898	0,000699	0,000559
K06901	0,000292	0,000444
K06911	0,00052	0,000707
K06915	0,000325	0,000329
K06925	0,000845	0,000559
K06926	0,000552	0,000164
K06929	0,000552	0,000214
K06940	0,000455	0,000838
K06941	0,000812	0,00051
K06942	0,000959	0,000904
K06949	0,000325	0,000345
K06950	0,000975	0,001282
K06958	0,000812	0,000625
K06959	0,000325	0,00069
K06960	0,000861	0,00074
K06968	0,000195	0,000526
K06969	0,000309	0,00074

K07736	0.0005150:	0.0002855:	0.000293634642648
K07737	0.0001026(	0.0001003:	0.000242942400034189
K07738	0.0003747!	0.0003913:	0.000258304473269704
K07787	0.0005030'	0.0004650'	0.000690419725145952
K07788	0.0001240!	0.0003471!	0.000000000000000000
K07789	0.0001151!	0.0002965!	0.000000000000000000
K07791	0.0001037(	0.0001424(	0.000246575753761125
K07798	0.0001678:	0.0001768	0.000213410717770688
K07799	0.0002078:	0.0001543:	0.000119201019148308
K07814	0.0006830'	0.0002119!	0.000367989631596472
K08138	0.0002529!	0.0002466	0.000598683158470255
K08191	0.0001908(	0.0002846:	0.000453722217346533
K08218	0.0009031'	0.0009378!	0.00100102637435475
K08282	0.0005561:	0.0001379!	0.000264941090225229
K08289	0.0002298'	0.0001759:	0.0002454282477715
K08300	0.0024324!	0.0024968(	0.00164388548870701
K08301	0.0002857(	0.0006517(	0.000711243106152575
K08303	0.0010270!	0.0010437(	0.00152748281948341
K08304	0.0011000!	0.0007976!	0.000747501595733886
K08305	0.0001108!	0.0002311(	0.000150862756585145
K08307	0.0005294!	0.0004970	0.000794467068931545
K08309	0.0006682(	0.0007915!	0.000566753942278933
K08311	0.0002342	0.0002747	0.000192491764030533
K08316	0.0004724(	0.0003125!	0.000299714919840845
K08344	0.0004381!	0.0003124'	0.000259771429921408
K08483	0.0004068	0.0006046:	0.000354783741941019
K08590	0.0001193:	0.0001628:	0.000262300702735159
K08591	0.0005662'	0.0004620!	0.000427690113283804
K08641	0.0007494(	0.0005088!	0.000563843396697472
K08676	0.0002986'	0.0002834!	0.000706775022594841
K08738	0.0002362(	0.0001703!	0.000139505405640797
K08884	0.0003968(	0.0002728!	0.000269031873479641
K08973	0.0003747!	0.0003276:	0.000259062663386021
K08974	0.0001230!	0.0001014!	0.000195745729119746
K08998	0.0001903(	0.0001679!	0.000181154132646202
K09001	0.0007495!	0.0007249:	0.000544433404114235
K09002	0.0001348!	0.0001287(	0.000319209791519089
K09007	0.0001976'	0.0001387!	0.000167822320301628
K09011	0.0002398:	0.0002252!	0.000560057630581677
K09013	0.0002920!	0.0001713	0.000370325013233999
K09014	0.0002560:	0.0003303!	0.000564371853654179
K09015	0.0001928:	0.0002695:	0.000457206025700164
K09117	0.0001820:	0.0001563:	0.000277586656510576
K09125	0.0002245(	0.0002506(	0.00037504064729483
K09134	0.0003628(	0.0001705:	0.000199937014196193
K09136	0.0003616	0.0001735	0.000245032104932606
K09155	0.0001696:	0.0001638(	0.000247246691221631
K09157	0.0001574'	0.0001257:	0.000190119405921292
K09181	0.0010120!	0.0004724!	0.00082841435550956
K09458	0.0017243!	0.0015289:	0.00162949217787293

K06973	9,75E-05	0,000296
K06989	0,000162	0,000378
K06997	0,000829	0,000526
K07001	0,000422	0,000855
K07003	0,000455	0,001085
K07005	0,000374	0,000723
K07007	0,000699	0,000362
K07010	0,000357	0,000427
K07011	0,000471	0,00074
K07012	0,000569	0,000247
K07023	0,000617	0,000378
K07024	0,00078	0,001496
K07025	0,001706	0,002597
K07029	0,000325	0,000164
K07033	9,75E-05	0,000214
K07034	0,000146	0,000345
K07035	0,000211	0,000575
K07037	0,000796	0,000773
K07042	0,000942	0,000838
K07043	0,000146	0,000164
K07045	0,000325	0,000444
K07047	0,000504	0,000148
K07052	0,000796	0,000756
K07053	0,001186	0,001611
K07056	0,00039	0,000477
K07058	0,000455	0,000296
K07062	0,001446	0,00268
K07075	0,00065	0,000707
K07079	0,000796	0,001841
K07082	0,000422	0,00074
K07088	0,000536	0,000625
K07089	0,000439	0,000674
K07090	0,00156	0,001184
K07096	9,75E-05	0,000148
K07098	0,00065	0,000378
K07101	0,000374	0,000559
K07107	0,001251	0,001463
K07133	0,001024	0,002548
K07138	0,000894	0,001085
K07146	0,000227	0,000164
K07164	0,000829	0,00069
K07166	0,000146	0,000395
K07171	0,00078	0,001595
K07172	0,000341	0,000855
K07173	0,000227	0,000378
K07213	0,000146	0,000148
K07216	0,000504	0,001233
K07219	0,000309	0,000773
K07238	0,000731	0,000674
K07239	9,75E-05	0,000164

K09686	0.0008314	0.0008304	0.00155264409439761
K09687	0.0003184	0.0004131	0.000552921421166195
K09691	0.0002018	0.0001424	0.000201776101980767
K09704	0.0002097	0.0002167	0.000494053709986439
K09710	0.0003630	0.0003003	0.000330842587403629
K09747	0.0003567	0.0002937	0.000232158468251714
K09748	0.0004722	0.0004111	0.000422307303922844
K09760	0.0008162	0.0008407	0.000755935069529366
K09761	0.0006827	0.0006053	0.000647628913063739
K09765	0.0005698	0.0004452	0.000482152740580546
K09773	0.0005165	0.0005336	0.000379864991077392
K09792	0.0002350	0.0002342	0.000198352534340968
K09797	0.0002789	0.0001842	0.000209931809522417
K09800	0.0004443	0.0006558	0.000551447640775309
K09808	0.0015444	0.0013230	0.00151687211856657
K09810	0.0007130	0.0006224	0.000633763415407505
K09811	0.0002014	0.0002391	0.000354903525588134
K09812	0.0002194	0.0001944	0.000353943762396168
K09815	0.0002457	0.0001599	0.000185074335042771
K09816	0.0002320	0.0001880	0.000257132834195529
K09817	0.0002014	0.0001492	0.000223118006495802
K09903	0.0009582	0.0007835	0.00081394361254452
K09949	0.0005848	0.0003412	0.000326686981227693
K09955	0.0001219	0.0001481	0.000286367392322715
K09973	0.0003122	0.0003459	0.000725453119732471
K09985	0.0002810	0.0001904	0.000166289158286398
K09987	0.0004684	0.0003170	0.000277152991793915
K09991	0.0001906	0.0001285	0.000112532620085341
K10126	0.0002804	0.0001403	0.00017260942287555
K10206	0.0004382	0.0001257	0.000455691171907357
K10536	0.0001678	0.0001062	0.000240309747156102
K10563	0.0008675	0.0006286	0.000566502962610602
K10716	0.0003179	0.0001835	0.000277151487293815
K10773	0.0008141	0.0006719	0.000755582278981372
K10778	0.0001184	0.0001242	0.000100101879003266
K10943	0.0003464	0.0001694	0.000202805032444407
K11068	0.0001321	0.0002193	0.000187237502018921
K11072	0.0002513	0.0001066	0.00012283084291697
K11085	0.0015521	0.0012666	0.00150430687340793
K11102	0.0004387	0.0003574	0.000259586719455863
K11103	0.0001687	0.0002677	0.000102786230829956
K11175	0.0002652	0.0002817	0.00037730440632953
K11473	0.0003630	0.0001620	0.000171806016466567
K11537	0.0001611	0.0001703	0.000381322894544758
K11645	0.0010142	0.0004670	0.000557598556152083
K11690	0.0004051	0.0002711	0.000201730890075394
K11717	0.0002698	0.0003892	0.000637137321073099
K11719	0.0001412	0.0001987	0.000133936082981352
K11741	0.0002631	0.0001862	0.000152624752956181
K11749	0.0011342	0.0009241	0.00110201064665517

K07240	0,000666	0,001282
K07243	0,000309	0,000542
K07246	0,000195	0,000197
K07258	0,000439	0,000707
K07259	0,001089	0,000312
K07260	0,000211	0,000181
K07263	0,000211	0,000411
K07277	0,000731	0,00051
K07282	0,000585	0,000378
K07301	0,000244	0,000214
K07304	0,000309	0,000197
K07305	0,000341	0,000148
K07306	0,000276	0,000707
K07315	0,000195	0,000148
K07319	0,001186	0,000427
K07321	0,000422	0,001052
K07334	0,000244	0,000378
K07335	0,000682	0,001496
K07337	0,000195	0,000493
K07391	0,000471	0,000723
K07399	0,000292	0,000362
K07402	0,000292	0,000247
K07403	0,000146	0,000427
K07406	0,000227	0,00051
K07407	0,000374	0,000526
K07443	0,000179	0,000444
K07447	0,000764	0,000411
K07456	0,000162	0,000312
K07460	0,000341	0,000378
K07462	0,000439	0,000756
K07464	0,00052	0,00023
K07473	0,00052	0,001364
K07478	0,00091	0,000838
K07483	0,000325	0,000164
K07491	0,000504	0,000477
K07497	0,000471	0,00023
K07502	0,000146	0,000362
K07507	0,000292	0,000608
K07552	0,000244	0,000132
K07560	0,000162	0,000214
K07566	0,001137	0,001348
K07567	0,00039	0,000756
K07568	0,000406	0,000756
K07576	0,000747	0,000756
K07584	0,000211	0,000575
K07636	0,000179	0,000214
K07665	9,75E-05	0,000197

K11752	0.0003954	0.0004687	0.000554474542235661
K11753	0.0007452	0.0006818	0.00074382347472919
K11754	0.0013466	0.0011367	0.00118092781429265
K11927	0.0004501	0.0005754	0.000678739186819604
K12251	0.0001964	0.0001722	0.000356837938811539
K12257	0.0003385	0.0003202	0.000799796550074026
K12267	0.0002228	0.0004157	0.000481736608976261
K12308	0.0001136	0.0001767	0.000248976519827459
K12340	0.0006883	0.0007374	0.000937368401072365
K12373	0.0003098	0.0004053	0.000736093003466733
K12410	0.0002113	0.0002416	0.000316014261243967
K12524	0.0003475	0.0004517	0.000822379628649018
K12541	0.0004037	0.0001075	0.000207726722552785
K12573	0.0008120	0.0010038	0.00122621653870811
K12574	0.0008825	0.0004952	0.000473132817443287
K12960	0.0004143	0.0001466	0.000424476378356056
K13017	0.0001984	0.0001013	0.000220079013050375
K13019	0.0001836	0.0001639	0.000395880689506396
K13038	0.0015234	0.0012563	0.00130495736986568
K13043	0.0001464	0.0001387	0.0003464680471069
K13292	0.0009884	0.0007910	0.000742987007711875
K13542	0.0004314	0.0001752	0.000260211470229176
K13590	0.0002872	0.0001905	0.00014799111049004
K13598	0.0009633	0.0004811	0.000562899802422956
K13599	0.0010120	0.0005230	0.000671850945764033
K13628	0.0002810	0.0002579	0.000199054522334715
K13747	0.0003565	0.0001856	0.000507508476023176
K13788	0.0005160	0.0001582	0.000381948958899147
K13789	0.0005021	0.0003173	0.000465586510945055
K13821	0.0011533	0.0011012	0.00125248756461294
K13888	0.0001344	0.0001939	0.000184022240745143
K13924	0.0005885	0.0002423	0.000289342526747742
K13963	0.0001220	0.0001156	0.000288723226100753
K13993	0.0002889	0.0001500	0.000261067517192982
K14155	0.0002430	0.0004114	0.000452480487705504
K14170	0.0002207	0.0002520	0.000206132858525227
K14392	0.0001889	0.0001970	0.000000000000000000
K14393	0.0001935	0.0002790	0.000104798009072059
K14441	0.0005445	0.0004191	0.000660859856622424
K14445	0.0003292	0.0001191	0.00027113568708502
K14534	0.0001504	0.0001431	0.000356045747140447
K14652	0.0005210	0.0005387	0.000731402350370087
K14742	0.0001125	0.0002109	0.000206527416524933
K15125	0.0003604	0.0033976	0.00209983343428467
K15270	0.0011616	0.0009783	0.000706091984018024
K15342	0.0002316	0.0002353	0.00032198275814055
K15371	0.0026909	0.0018275	0.00157887960575415
K15633	0.0019614	0.0012373	0.00155334842855914
K15634	0.0002174	0.0002050	0.000149393626346947
K15635	0.0004048	0.0001681	0.000526123844333662

K15724	0.00014054	0.00017136	0.000119085923728128
K15726	0.00047929	0.00093005	0.00101648443695076
K15738	0.00071800	0.00076173	0.00118949556397973
K15778	0.00044554	0.00034007	0.000325588272418957
K15923	0.00020084	0.00020149	0.000475267325863987
K15987	0.00047927	0.00068337	0.000915667247281645
K16011	0.00035322	0.00024843	0.000176342853066733
K16052	0.00029473	0.00020678	0.000342412775019703
K16089	0.00051502	0.00067755	0.00121853906771948
K16264	0.00072550	0.00064912	0.00069882970993517

Rg_1
0,00057
0,000356
0,000726
0,000527
0,000641
0,000598
0,000128
0,000157
0,000954
0,000128
0,000157
0,000114
0,000441
0,000356
0,000384
0,000598
0,000869
0,001025
0,000698
0,00168
0,00047
0,000214
0,001011
0,00037
0,00074
0,000328
0,000527
0,001011
0,000413
0,000313
0,000456
0,000242
0,000598
0,001054
0,000114
0,000114
0,001452
0,000199
0,000313
0,000114
0,000114
0,000441
0,000441
0,000441
0,000441
0,001054
0,001054

0,000897
0,00094
0,000399
0,000399
0,000313
0,000313
0,000313
0,000157
0,001225
0,000983
0,000142
0,000142
0,000142
0,000114
0,000527
0,000427
0,000456
0,000157
0,000883
0,002364
0,000128
0,000527
0,000812
0,000812
0,000641
0,000157
0,000313
0,000869
0,000114
0,000214
0,000214
0,001509
0,001538
0,001467
0,000228
0,000142
0,000214
0,000214
0,000214
0,000228
0,000214
5,7E-05
0,000427
0,000214
0,000555
0,000769
0,00121
0,000313
0,000342
0,000128

0,000128
0,00047
0,000313
0,000142
0,001054
0,000157
0,000968
0,000683
0,000313
0,000128
0,000812
0,001481
0,000726
0,000199
0,000157
0,001039
0,001025
8,54E-05
9,97E-05
0,000171
0,000783
0,001267
0,000783
0,001951
0,001025
0,000968
0,000641
0,000598
0,000399
0,000897
0,002848
0,000185
0,000427
0,000612
0,000954
0,000142
0,000128
0,000669
0,000157
0,000456
0,000541
0,001011
0,001168
0,000399
0,000498
0,001153
0,000242
0,000128
0,000228
5,7E-05

0,000114
0,000328
0,000171
0,000128
0,000342
0,000598
0,000328
0,000157
0,000171
0,000669
0,000584
0,000214
0,000214
0,000612
0,000356
0,000199
0,000612
0,000185
0,000598
0,001011
0,000299
0,00094
0,000598
0,000854
0,000527
0,000427
0,000712
0,000313
0,000171
0,000498
0,00057
0,001025
0,001025
0,001196
0,000598
0,000598
0,000612
0,000812
9,97E-05
0,000968
0,000712
0,000669
0,001495
0,000926
0,001111
0,00094
9,97E-05
0,00047
0,00074
0,000484

0,000384
0,000427
0,00094
0,000598
0,000157
0,000755
0,001025
0,001011
0,000598
0,000513
0,00047
0,000926
0,001282
0,000584
0,000128
0,000384
0,00057
0,000712
0,000171
0,001025
0,000698
0,001025
0,000683
0,000726
0,000612
0,000313
0,001025
0,00084
0,000384
0,000612
0,000954
0,000313
0,000527
0,001025
0,000441
0,000669
0,000712
0,001111
0,000342
0,000384
0,000712
0,000128
0,001054
0,001111
0,000484
0,000242
0,000456
0,000328
0,000185
0,000726

9,97E-05
0,000598
0,000812
0,000968
0,000584
0,001011
0,000313
0,000683
0,000157
0,000498
0,000185
0,001111
0,000427
0,000726
0,000356
0,000456
0,00057
0,00037
0,000555
0,000983
0,000911
0,000513
0,000627
0,000413
0,000527
0,000171
0,001068
0,000128
0,001652
0,001766
9,97E-05
0,001011
0,000513
0,001025
0,000185
0,000484
0,000755
0,000413
0,000498
0,000313
0,000256
0,000285
0,000584
0,000328
0,000726
0,000427
0,000441
0,00047
0,00037
0,000598

0,000128
0,000441
0,000755
0,001082
0,000968
0,000228
0,000641
0,000399
0,000413
0,001054
0,00037
0,001467
0,000755
0,000399
0,001438
0,000427
0,000171
0,000185
0,00057
0,00094
0,000456
0,000812
0,001054
0,00037
0,001039
0,000456
0,000171
9,97E-05
0,00057
0,002065
0,000954
0,000456
0,000555
0,00047
0,000427
0,000313
0,001823
0,000484
0,000157
0,000712
0,000641
0,000498
0,000185
0,00074
0,000983
0,000171
0,000683
0,000498
0,000456
0,000926

0,001011
0,000911
0,000826
0,000712
0,001096
0,000683
0,000869
0,000584
0,00057
0,000598
0,000171
0,001054
0,001908
8,54E-05
0,000185
0,000171
0,000142
0,001139
0,001239
0,000669
0,000598
0,001353
0,001082
0,000869
0,000869
0,000812
0,001025
0,00037
0,000142
0,000171
0,000769
0,000726
0,000128
0,000769
0,000356
0,000983
0,001196
0,001039
0,000128
0,000513
0,000869
0,000911
0,000384
0,001937
0,001951
0,000328
0,001324
0,00047
0,00074
0,001239

0,000968
0,000655
0,000555
0,000513
0,000669
0,000356
0,000342
0,000157
0,00037
0,000128
0,00037
0,000313
0,000114
0,000598
0,000712
0,000399
0,000342
0,00037
0,001524
0,000641
0,000128
0,001025
0,00094
0,000598
0,001054
0,001723
0,000655
0,000541
0,000157
0,000698
0,001054
0,000413
0,000128
0,001011
0,000541
0,000726
0,000256
0,000926
0,000584
0,000555
0,001751
0,000456
0,000498
0,000812
0,000413
0,000612
0,000228
0,000342
0,001025
0,001025

0,001025
0,001025
0,001025
0,001025
0,001011
0,001025
0,001025
0,001025
0,000498
0,000498
0,000541
0,001082
0,00074
0,001082
0,000413
0,001025
0,000726
0,001025
0,001025
0,00057
0,000869
0,001381
0,000185
0,000185
0,000356
0,000413
0,00094
0,00074
0,000598
0,001082
0,000712
0,001039
0,001011
0,001011
0,000498
0,000712
0,001011
0,001011
0,000926
0,000726
0,001025
0,000655
0,000612
0,000598
0,000598
0,00094
0,001039
0,001581
0,000484
0,00121

0,000555
0,00168
0,001054
0,001068
0,000726
0,000427
0,00131
0,000242
0,001452
0,000584
0,001168
0,001282
0,00057
0,000869
0,000911
9,97E-05
9,97E-05
9,97E-05
0,000328
0,001509
0,000769
0,001125
0,001225
0,00215
0,00215
0,002933
0,001737
0,002905
0,002948
0,000228
0,000427
5,7E-05
0,003503
0,00393
0,002577
0,00141
0,002492
0,001566
0,004258
0,004329
0,004927
0,004528
0,005667
0,000627
0,000655
0,000669
0,000669
0,000883
0,000128
0,000342

0,000342
0,000342
0,000342
0,001481
0,001794
0,000911
0,000669
0,000356
0,000356
4,27E-05
0,001054
0,00188
0,00047
0,000413
0,000413
0,000157
0,000456
0,00037
0,001096
0,001096
0,000797
0,001196
0,000883
0,000911
0,000883
0,000883
0,000883
0,000171
0,000883
0,000883
0,000627
0,000627
0,00047
0,000627
0,000527
0,001153
0,000413
0,000328
0,000413
0,000726
0,000484
0,001168
0,000441
0,00084
0,00074
0,000427
8,54E-05
0,000555
0,000612
5,7E-05

5,7E-05
0,000698
0,001039
0,001111
0,001025
0,000954
0,000726
0,000313
0,001652
0,001025
0,001025
0,00057
0,001025
0,001139
0,00057
0,002008
0,001025
0,001025
0,001481
0,000983
0,000413
0,00037
0,000413
0,000413
0,000441
0,00047
0,000441
0,00047
0,00094
0,000441
0,000441
0,000441
0,00074
0,000441
0,000441
0,000441
0,000683
0,000128
0,000441
0,000441
0,000228
0,000427
0,000128
0,000441
0,000157
0,000128
0,000441
0,000441
0,000128
0,000441

0,000441
0,000128
0,000157
0,000669
0,001922
0,001894
0,001253
0,000897
0,000456
0,00037
0,001054
0,001054
0,000342
0,000214
9,97E-05
0,000313
0,001054
0,001125
0,000313
0,000911
0,000911
0,000897
0,000384
0,000242
0,001039
0,001025
0,000726
0,000242
0,000171
0,00074
0,003417
0,000157
0,000171
0,000228
0,000313
0,000313
0,000171
0,000555
0,00141
0,000612
0,000712
0,000399
0,000399
0,000399
0,001011
0,000384
0,000384
0,000698
0,000399
0,001025

0,001438
0,000527
0,000527
0,00037
0,000983
0,002805
0,000413
0,000157
0,000313
0,000313
0,000313
0,000313
0,000812
0,000413
0,000413
0,000413
0,000313
0,000313
0,000513
0,000854
0,000199
0,000527
0,000712
0,001025
0,000598
0,001025
0,000427
0,001011
0,001025
0,000726
0,001025
0,001025
0,001025
0,001025
0,001025
0,001025
0,001025
0,001025
0,001025
0,000726
0,000726
0,001025
0,001025
0,001025
0,001011
0,001025
0,00074
0,000726
0,000726
0,000584

0,001054
0,00074
0,001054
0,000968
0,000726
0,000726
0,000726
0,000726
0,001025
0,001025
0,001011
0,000627
0,001025
0,001025
0,000726
0,001025
0,001054
0,000612
0,001025
0,001025
0,000726
0,001025
0,000726
0,001025
0,001011
0,000726
0,001039
0,001025
0,000726
0,000726
0,001025
0,000726
0,001025
0,001025
0,001025
0,000171
0,001025
0,000926
0,001025
0,000926
0,001025
0,001025
0,001766
0,002406
0,000513
0,001994
0,000997
0,000328
0,000328
0,001153

0,000185
0,000157
0,000427
0,000441
0,00074
0,000427
0,000513
0,00074
0,000726
0,000271
0,001025
0,000299
0,000712
0,000114
0,001025
0,001025
0,000541
0,000498
9,97E-05
0,000142
0,000171
0,000997
0,000427
0,000484
0,000441
0,000513
0,001282
0,000983
0,000997
0,000669
0,00057
0,000157
0,00037
0,000456
0,000171
0,000157
0,000399
0,00037
0,00037
0,000271
0,000199
0,00205
0,000911
0,001039
0,000456
0,000911
0,000669
0,001951
0,000484
0,001766

0,000513
0,001011
0,000726
0,000328
0,000157
0,000527
0,000897
0,000769
0,000968
0,000911
0,000527
0,000313
0,001011
0,000812
0,001381
0,000655
0,00094
0,000128
0,000726
0,000527
0,000484
0,000783
0,000783
0,000214
0,001011
0,000612
0,000712
0,000712
0,00094
0,001153
0,001011
0,001025
0,001011
0,001025
0,000441
0,000142
0,001025
0,001025
0,000726
0,000997
0,000513
0,000313
0,000598
0,000427
0,000157
0,001011
0,000185
0,001054
0,000997
0,000869

0,000997
0,001182
0,000612
0,000313
0,000214
0,001011
0,001011
0,000968
0,000997
0,000157
0,000142
0,000627
0,001025
0,001011
0,001011
0,000399
0,000498
0,000883
0,000883
0,000498
0,000527
0,000612
0,001025
0,000698
0,000598
0,001011
0,001011
0,000427
0,000427
0,000527
0,000541
0,000541
0,000128
0,001011
0,001011
0,001025
0,001495
0,001225
0,001025
0,000171
0,000484
0,001267
0,000384
0,001025
0,001367
0,001025
0,000456
0,001025
0,00057
0,000427

0,001139
0,001025
0,001025
0,000655
0,000527
0,000399
0,000142
0,000598
0,000441
0,000399
2,85E-05
0,000313
0,001011
0,000598
0,000612
0,000584
0,000171
0,000541
0,00047
0,000698
0,000441
0,000356
0,000128
0,00037
0,000498
0,000527
0,000399
0,000513
0,000199
7,12E-05
0,000541
0,000427
0,000555
0,000983
0,000456
0,00037
0,001737
0,000427
0,000897
0,000142
0,001011
0,000527
0,000826
0,000384
0,000413
0,000712
0,000114
0,00037
0,000997
0,000627

0,000954
0,000328
0,000513
0,000726
0,000698
0,000612
0,000627
0,001025
0,001381
0,000427
0,001011
0,000413
0,001467
0,00047
0,001894
0,000313
0,001025
0,001039
0,001039
0,000555
0,000114
0,000456
0,000157
0,000313
0,001011
0,000328
0,001011
0,000441
0,001239
0,00094
0,000755
0,000555
0,000142
0,000441
0,000612
0,000413
0,000384
0,000883
0,000214
0,000456
0,000683
0,001025
0,001153
0,000228
0,000313
0,000712
0,000641
0,000313
0,001196
0,000555

0,000997
0,000555
0,000313
0,000441
9,97E-05
0,00057
0,000612
0,000413
0,000313
0,00037
0,000612
0,000769
0,000128
0,00047
0,000484
0,00094
0,000313
0,000313
0,000313
0,000427
0,000427
0,000683
9,97E-05
0,00094
0,000413
0,000171
0,000328
0,000356
0,000413
0,000199
0,000313
0,000399
0,000484
0,000427
0,00037
0,001182
0,00094
0,000185
0,000356
0,000441
0,001039
0,000527
0,000185
0,004528
0,000256
0,000612
0,000584
0,000527
0,001011
0,000641

0,000128
0,000612
0,001025
0,001552
0,000726
0,000484
0,000484
0,000356
0,000214
0,000484
0,00037
0,000199
0,000413
0,000641
0,000612
0,000128
0,000384
0,000612
0,000456
0,000498
0,000555
0,000427
0,000313
0,000983
0,000783
0,000541
0,000513
0,000342
0,000142
0,001695
0,000413
0,000456
0,000328
0,000783
0,000313
0,000598
0,000299
0,000726
0,000413
0,00047
0,000683
0,000698
0,001025
0,000313
0,00141
0,000797
0,000555
0,000897
0,000427
0,000598

0,000242
0,000328
0,000712
0,000641
0,000854
0,000655
0,000484
0,000328
0,000641
0,000456
0,00057
0,001296
0,002549
0,000142
0,000157
0,000313
0,000456
0,000954
0,00094
0,000185
0,000384
9,97E-05
0,000655
0,001623
0,000413
0,000242
0,002321
0,000641
0,001595
0,000612
0,00037
0,000527
0,001253
0,000114
0,000641
0,000484
0,001524
0,002264
0,00121
9,97E-05
0,000926
0,000313
0,001367
0,000726
0,000313
0,000142
0,001068
0,000669
0,000869
0,000214

0,000968
0,000441
9,97E-05
0,000484
0,000598
0,000114
0,000399
0,000683
0,000612
0,000142
0,000114
9,97E-05
0,000612
0,000114
0,000897
0,000926
0,000313
0,001253
0,000413
0,000612
0,000328
0,000157
0,000356
0,000413
0,000527
0,000399
0,000612
0,000256
0,000313
0,000598
0,000456
0,001139
0,001025
2,85E-05
0,000342
0,000157
0,000313
0,00047
7,12E-05
0,000171
0,001438
0,000641
0,000669
0,000883
0,000484
0,000171
0,000157
