# Supplementary figures and images for: Gut microbiota dynamics and functionality in Reticulitermes grassei after a 7-day dietary shift and ciprofloxacin treatment
Source: PLoS One. 2018 Dec 27;13(12):e0209789. doi: 10.1371/journal.pone.0209789 (PMC6307977; doi:10.1371/journal.pone.0209789)

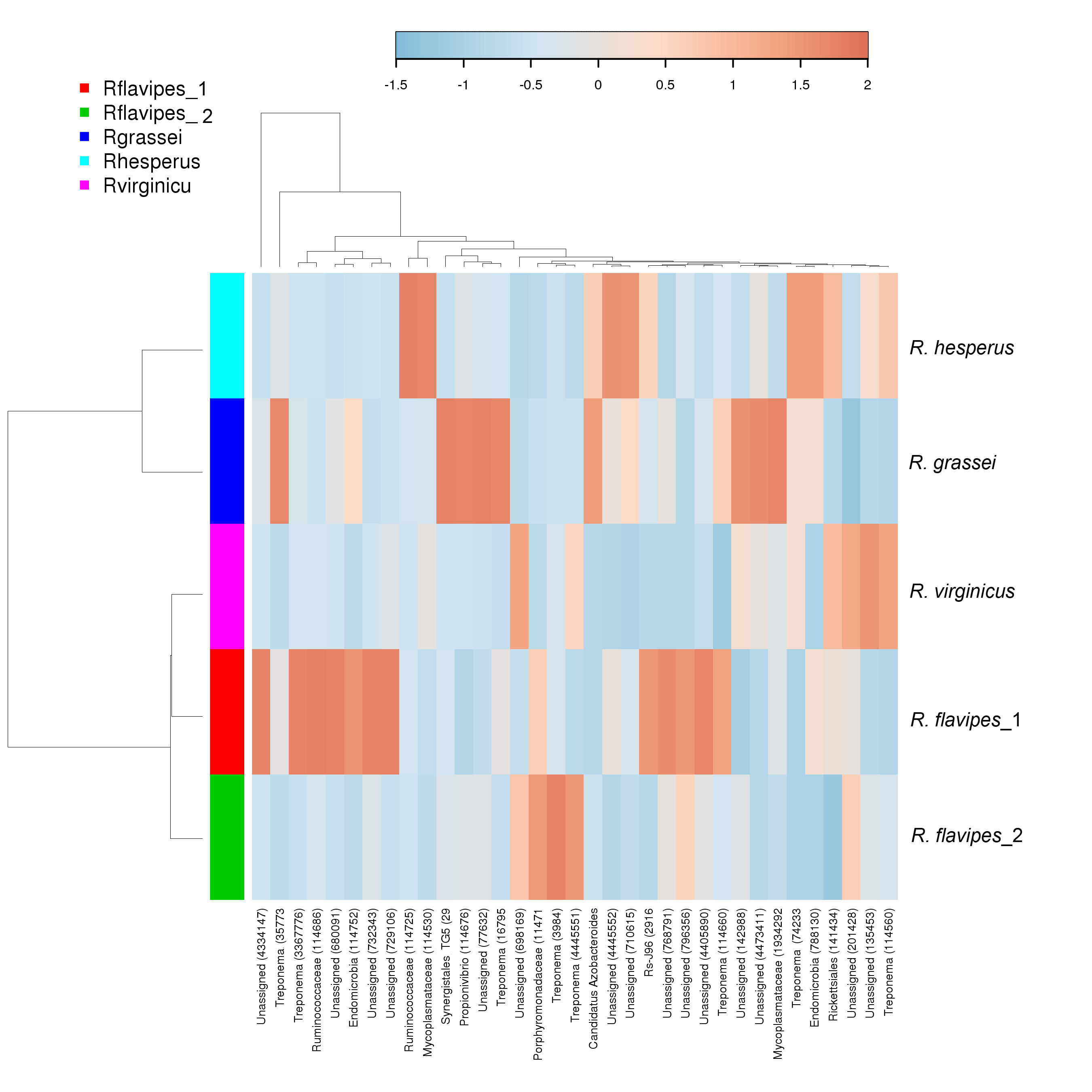

Supplement: S1 Fig — Several representative OTUs could be classified at genus level. (TIF) [file pone.0209789.s002.tif]

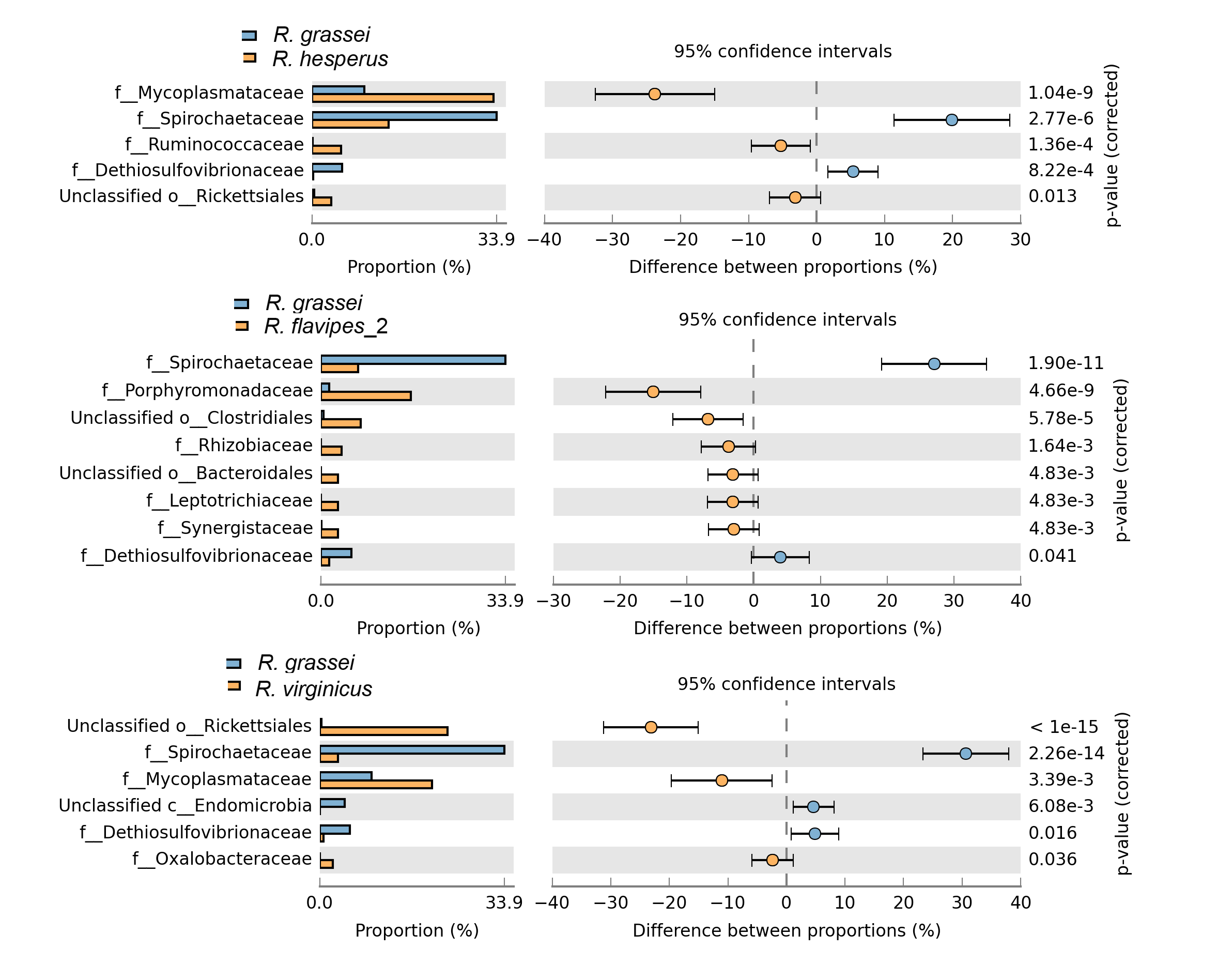

Supplement: S2 Fig — Corrected P values are shown at right. (TIF) [file pone.0209789.s003.tif]

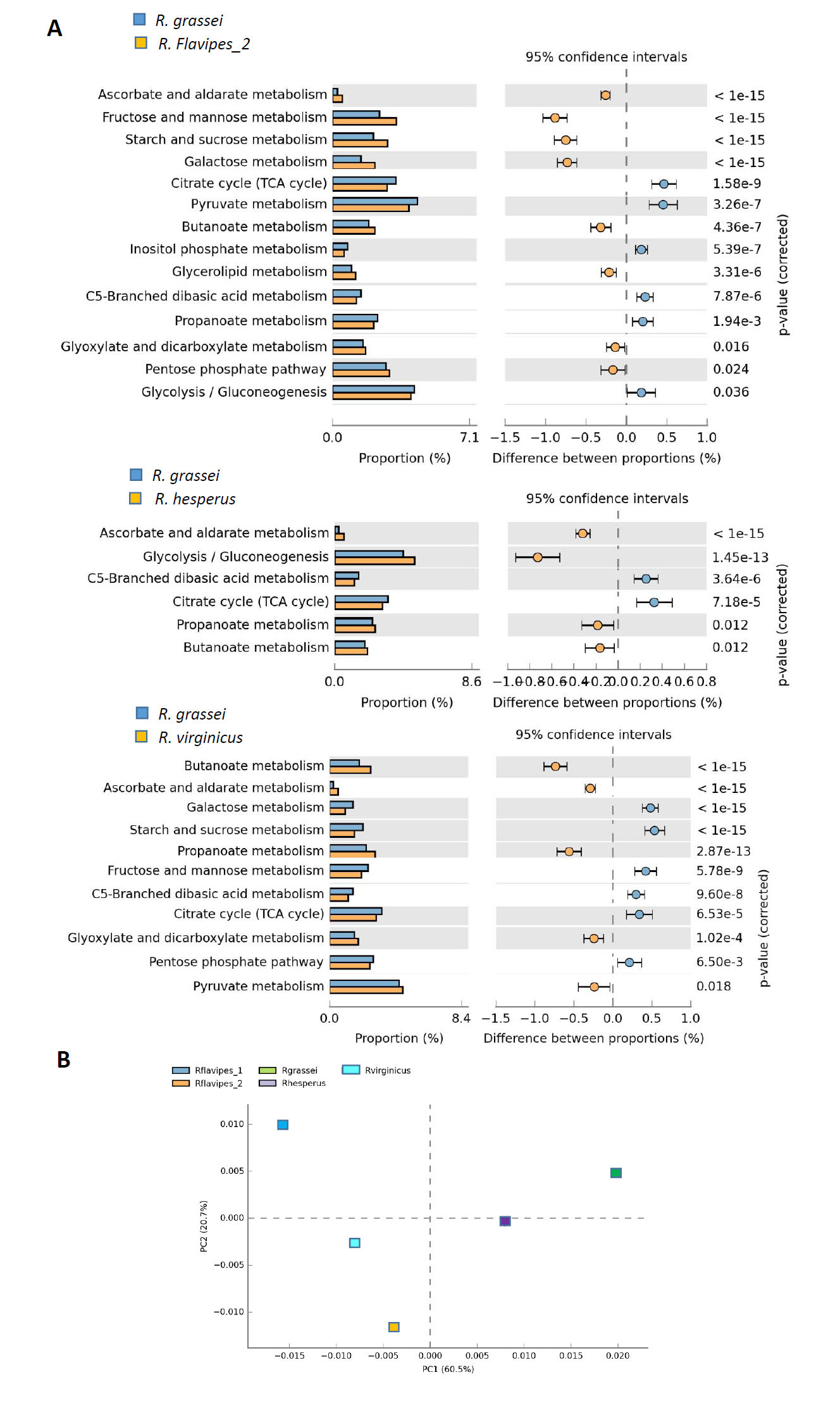

Supplement: S3 Fig — (A) Extended error bar plot identifying significant differences between mean proportions of carbohydrate pathways in pairwise Reticulitermes species. (B) PCA of carbohydrate pathways from Reticulitermes species. (TIF) [file pone.0209789.s004.tif]

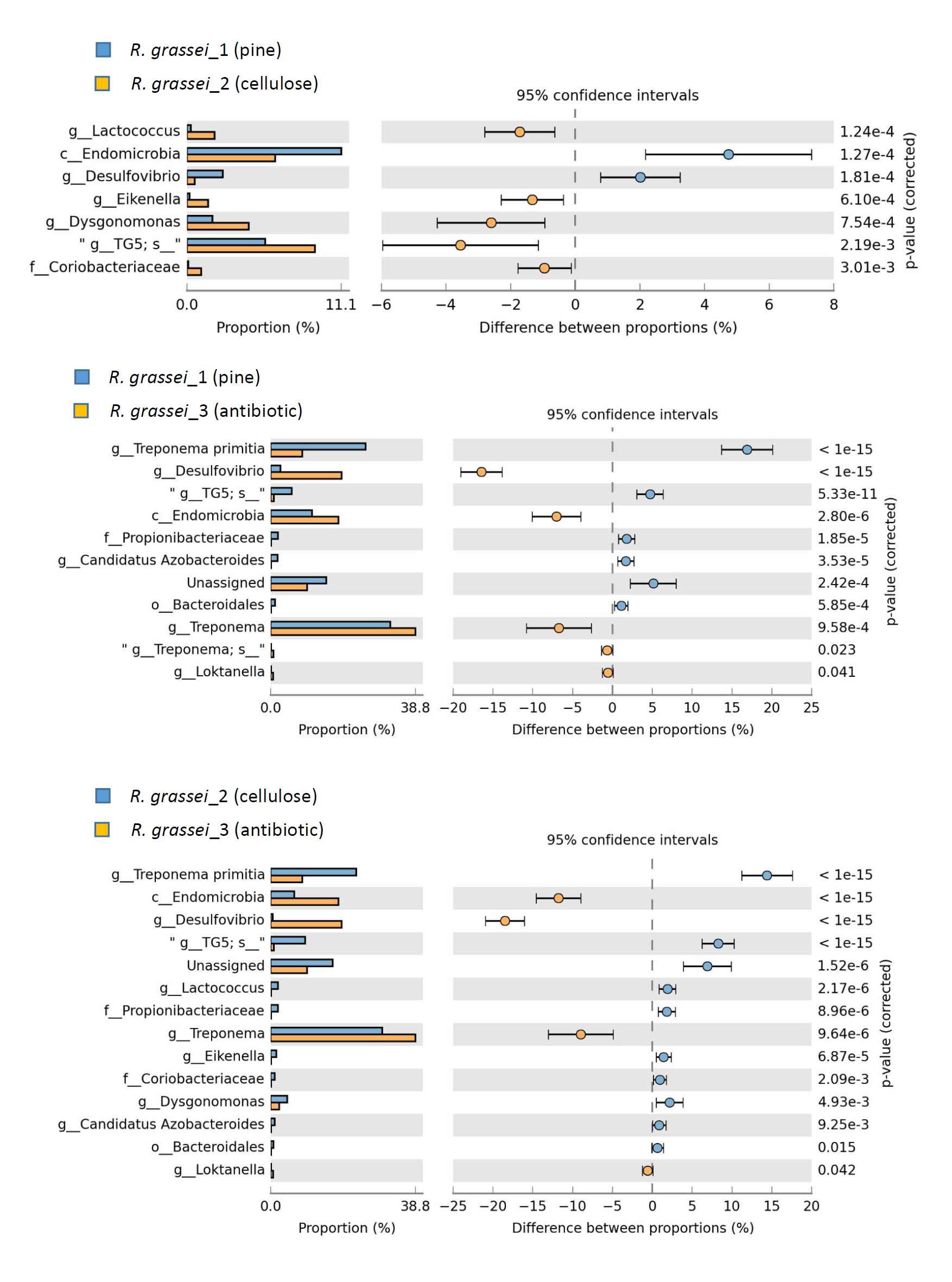

Supplement: S4 Fig — Extended error bar plot identifying significant differences between mean proportions of bacterial taxa in pairwise Reticulitermes grassei treatments, wood-diet, cellulose-diet and ciprofloxacin treatment. (TIF) [file pone.0209789.s005.tif]

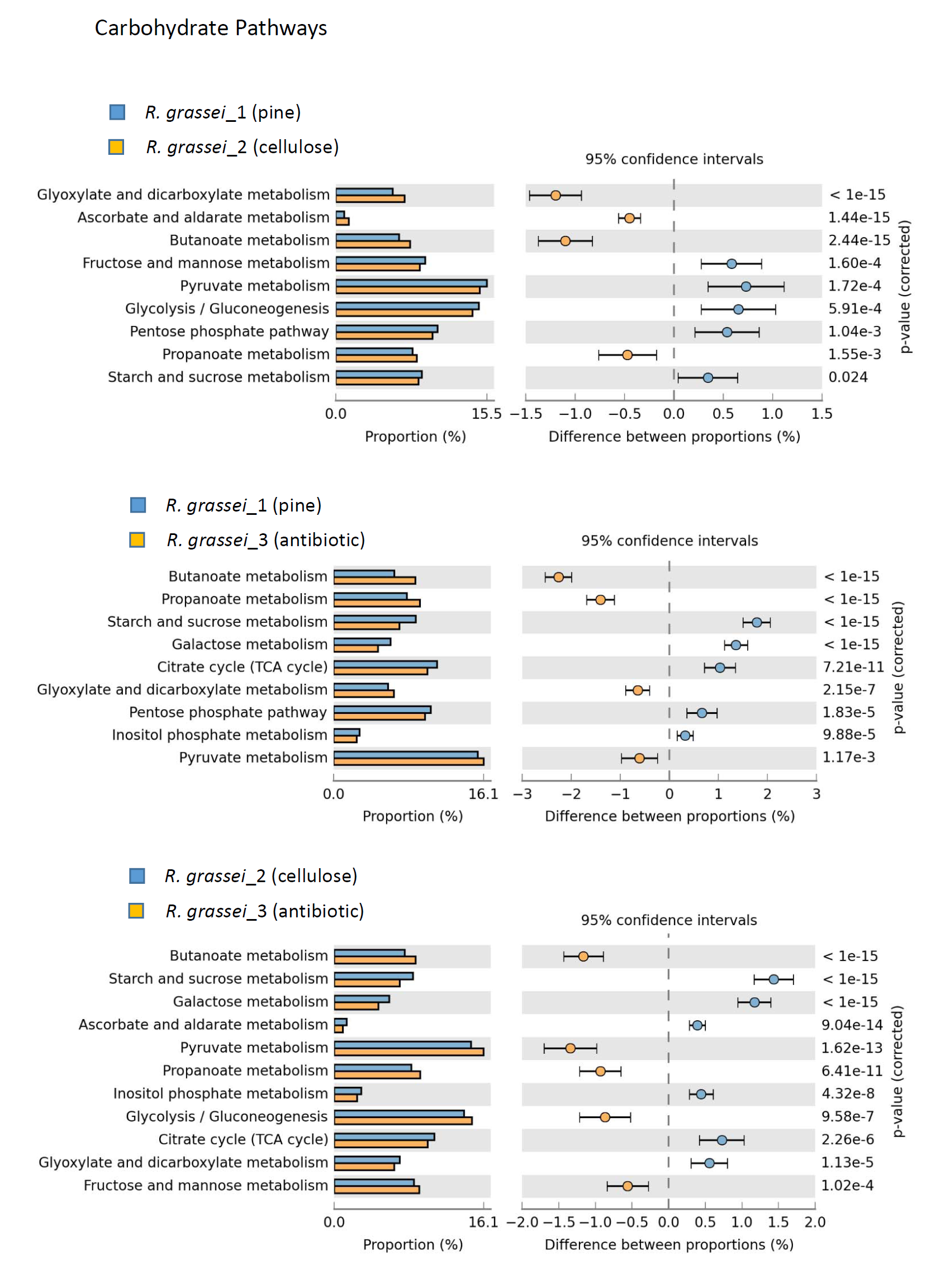

Supplement: S5 Fig — Extended error bar plot identifying significant differences between mean proportions of carbohydrate pathways in pairwise Reticulitermes grassei treatments, wood-diet, cellulose-diet and ciprofloxacin treatment. (TIF) [file pone.0209789.s006.tif]

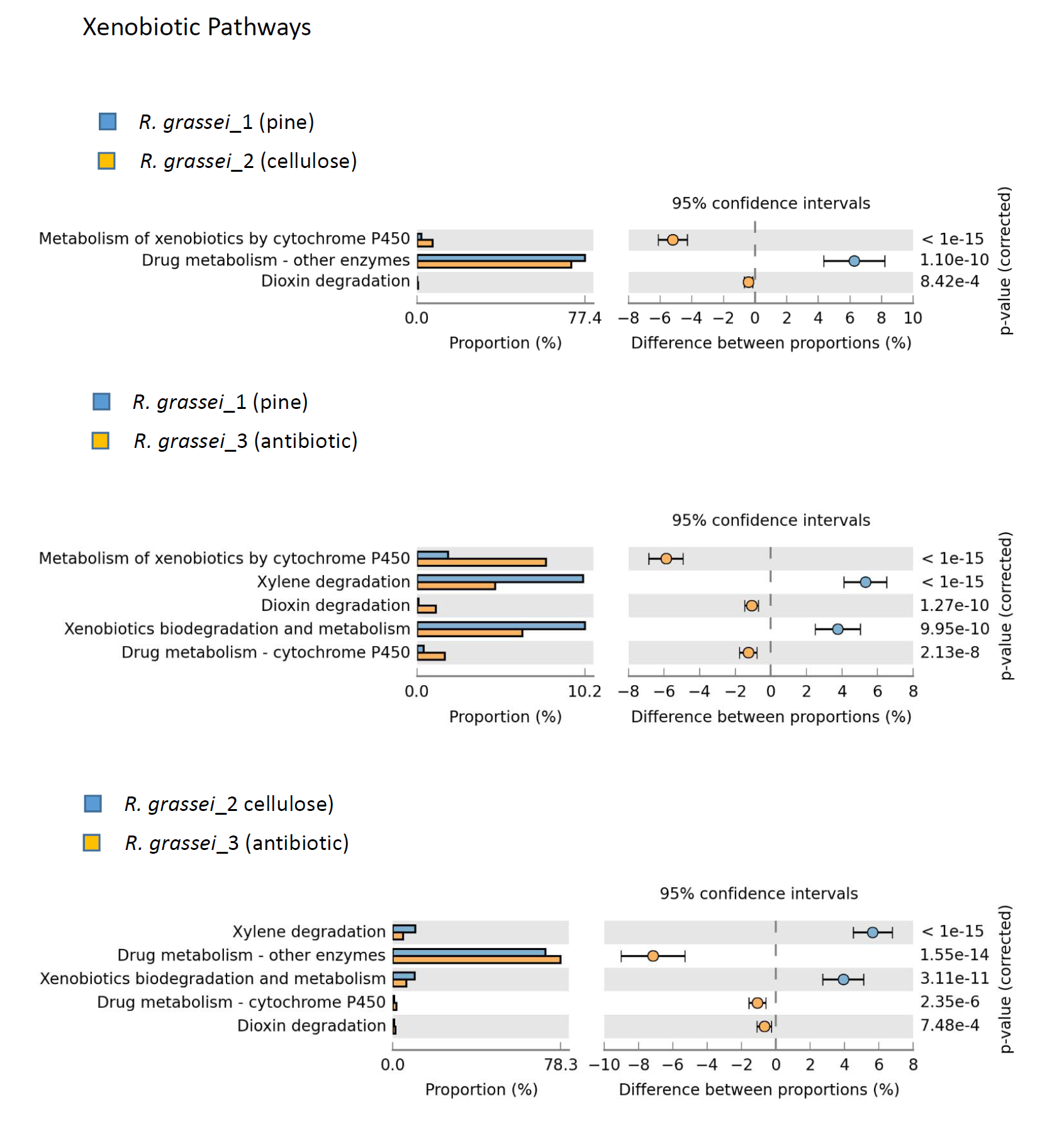

Supplement: S6 Fig — Extended error bar plot identifying significant differences between mean proportions of xenobiotic pathways in pairwise Reticulitermes grassei treatments, wood-diet, cellulose-diet and ciprofloxacin treatment. (TIF) [file pone.0209789.s007.tif]

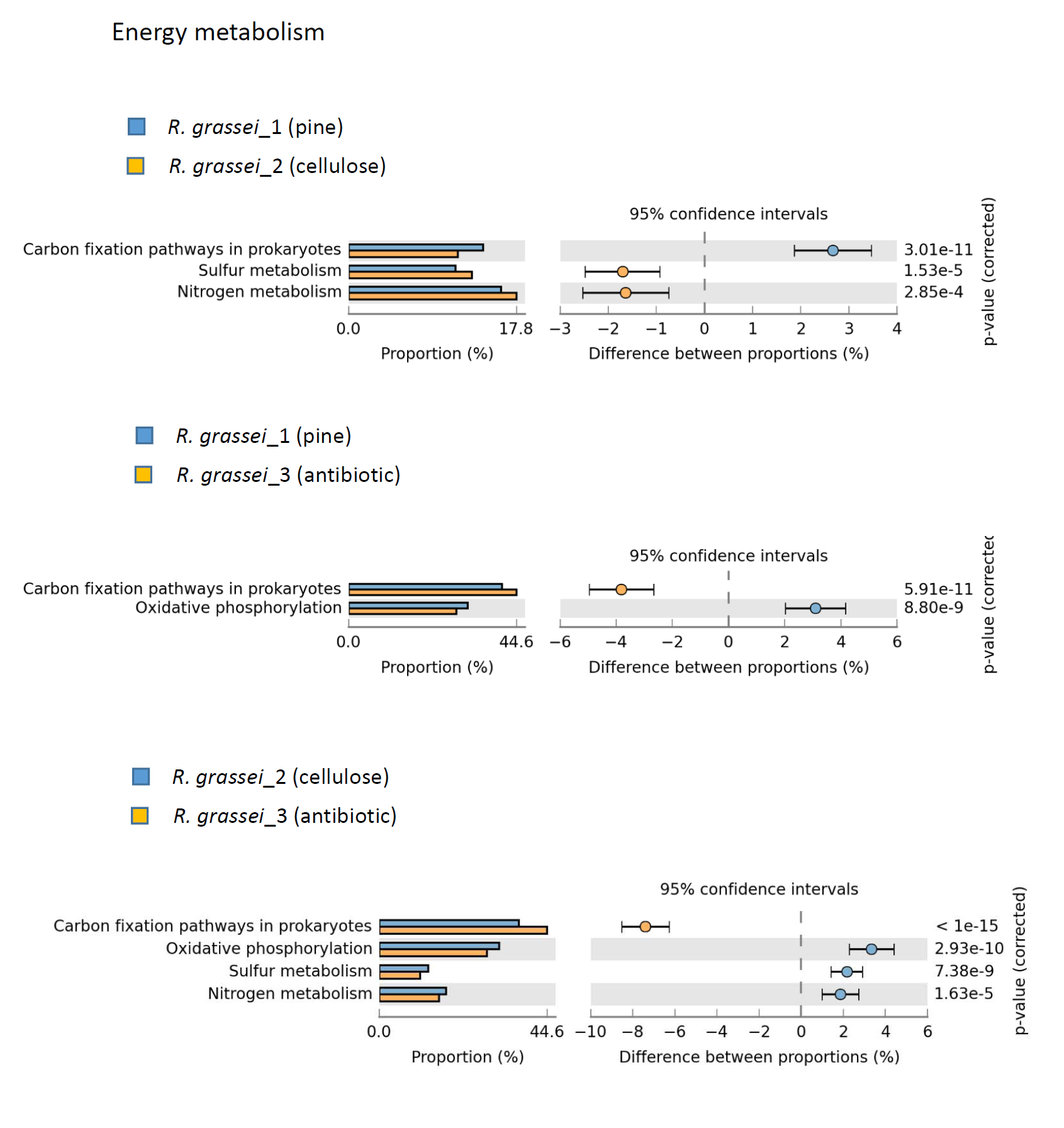

Supplement: S7 Fig — Extended error bar plot identifying significant differences between mean proportions of energy pathways in pairwise Reticulitermes grassei treatments, wood-diet, cellulose-diet and ciprofloxacin treatment. (TIF) [file pone.0209789.s008.tif]

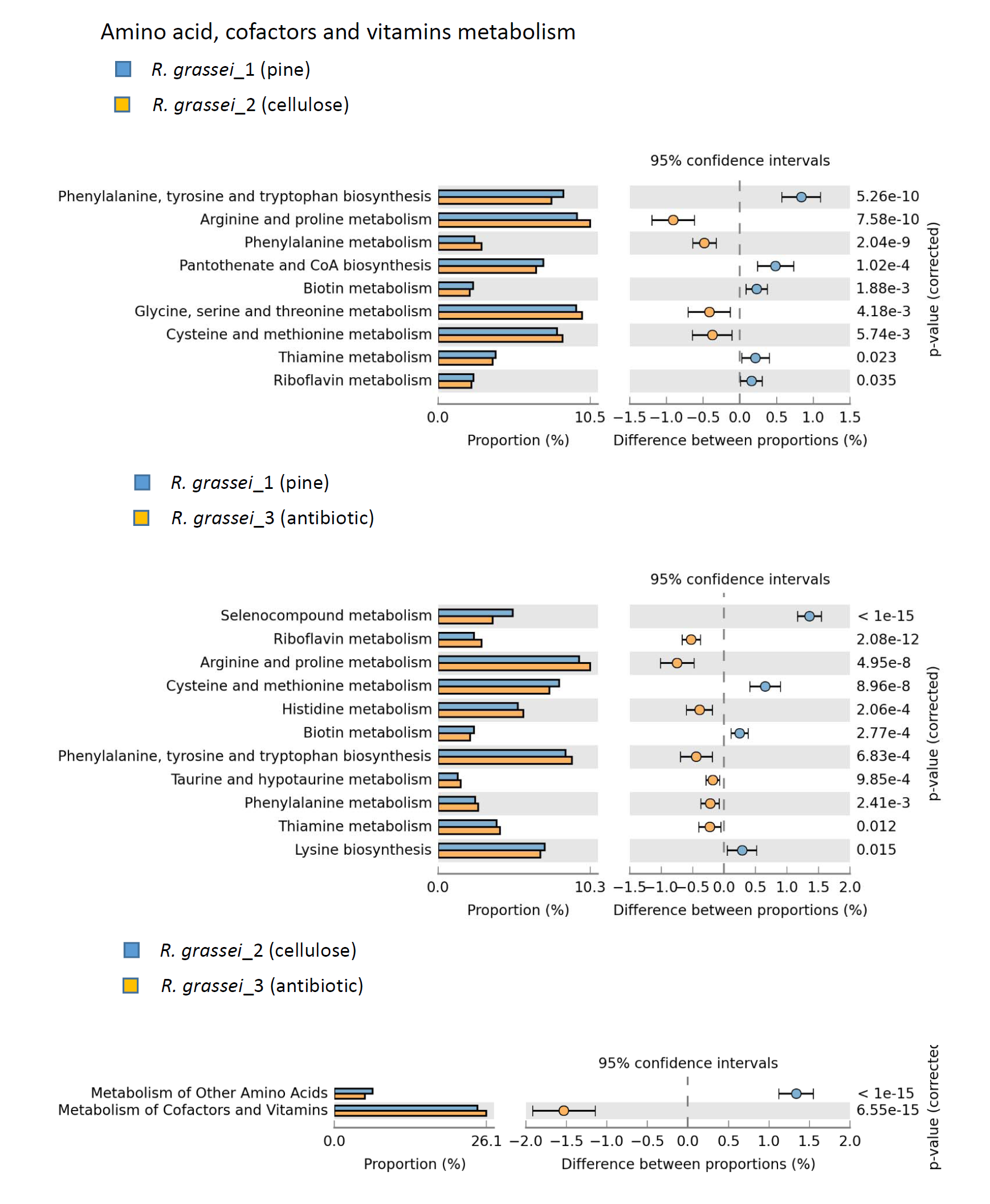

Supplement: S8 Fig — Extended error bar plot identifying significant differences between mean proportions of amino acid, cofactors and vitamins metabolism in pairwise Reticulitermes grassei treatments, wood-diet, cellulose-diet and ciprofloxacin treatment. (TIF) [file pone.0209789.s009.tif]

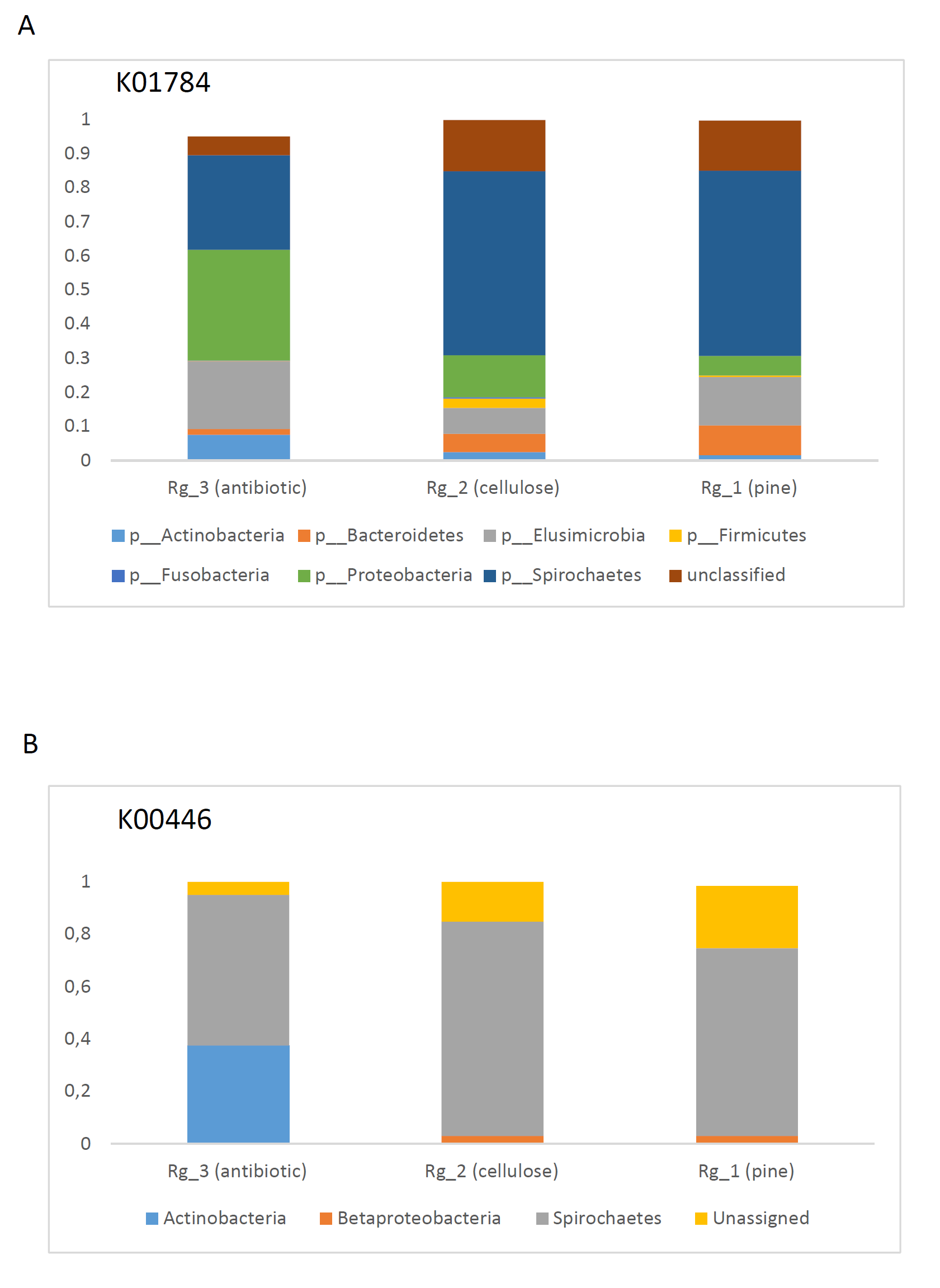

Supplement: S9 Fig — (A) Phyla contribution by percent of sample respect to K01784 (galactose pathway). (B) Phyla contribution by percent of sample respect to K00446 (xylene degradation). (TIF) [file pone.0209789.s010.tif]
